# Microwave-Assisted P–C Coupling of the Less Reactive Chlorobenzene and >P(O)H Reagents in the Absence of the Usual Mono- and Bidental P-Ligands

**DOI:** 10.3390/molecules30051045

**Published:** 2025-02-25

**Authors:** Bianka Huszár, Zoltán Mucsi, György Keglevich

**Affiliations:** 1 Department of Organic Chemistry and Technology, Faculty of Chemical Technology and Biotechnology, Budapest University of Technology and Economics, Műegyetem rkp. 3., 1111 Budapest, Hungary; huszar.bianka@vbk.bme.hu; 2 Faculty of Materials and Chemical Sciences, University of Miskolc, 3515 Miskolc, Hungary; zoltan.mucsi@uni-miskolc.hu

**Keywords:** chloroarenes, decreased reactivity, secondary phosphine oxides, diethyl phosphite, ethyl phenyl H-phosphinate, C–P coupling, Hirao reaction, microwave, theoretical calculations

## Abstract

The so far unattended version of the Hirao reaction involving the coupling of the less reactive chloroarenes with >P(O)H reagents, such as diarylphosphine oxides, diethyl phosphite, and ethyl phenyl-*H*-phosphinate, was investigated in detail using Pd(OAc)_2_ as the catalyst precursor, and applying some excess of the P-reagent to provide the ligand via its trivalent tautomeric (>P-OH) form. In the presence of triethylamine, no P–C coupling took place, meaning that there was a need for a stronger base, an alkali carbonate. The solvent had a significant effect on the efficiency of the Hirao reaction. The optimum conditions (10% of the Pd(OAc)_2_, 1.3 equiv. of the P-reagent, 1.1 equiv. of the alkali carbonate, 135–150 °C) explored herein were applied in the synthesis of diaryl-phenylphosphine oxides, aryl-diphenylphosphine oxides, diethyl arylphosphonates, and ethyl diphenylphosphinate. Theoretical calculations performed at the M06-2X/6-31G(d,p)[PCM(MeCN)] level also justified coupling with the chloroarenes under appropriate conditions, and were in accord with the experimental results revealing the unsuitability of triethylamine as a base and the need for an alkali carbonate. The new protocol elaborated herein is more practical and “greener” than the version with bromoarenes, and embraces a wide substrate scope.

## 1. Introduction

A heteroatomic variation in the C–C cross-coupling reactions is C–P coupling, discovered by Hirao in 1980 [1,2,3]. In C–P coupling, the original Pd(PPh_3_)_4_ catalyst is replaced by different Pd(II) salts that are used together with a variety of mono- and bidental P-ligands [4,5]. The mechanism of the Hirao reaction comprises four steps [4,6]. P–C couplings have also been carried out under MW conditions [7,8]. Later on, Ni- and Cu-catalyzed reactions were also elaborated [3,9], and arylboronic acids were also applied as starting materials as a halogene-free approach [10]. Since then, these methods have often been used to provide dialkyl arylphosphonates or tertiary phosphine oxides through the reaction of aryl bromides/aryl iodides with dialkyl phosphites or secondary phosphine oxides, respectively [3].

Montchamp et al. investigated the first P–C coupling reactions of heteroaryl chlorides or a few aryl chlorides with an electron-withdrawing substituent in the aryl ring using diisopropyl phosphite as the >P(O)H species in the presence of Pd(OAc)_2_ catalyst precursor and dppf as the ligand [11].

Zakirova et al. developed an efficient method for C–P bond formation via the palladium-acetate-catalyzed reaction between dichloro-substituted five- and six-membered *N*-heterocycles and secondary phosphine oxides using dppf ligand in the presence of Cs_2_CO_3_. Mono- and bis(phosphinoyl) derivatives of pyridine, 2,2′-bipyridine, 1,10-phenantroline, quinoline, imidazole, and thiazole, were obtained in good yields using Ph_2_P(O)H and Oct_2_P(O)H as the P-reagents [12,13].

Our research group developed a MW-promoted P–C coupling reaction, in which there was no need for the usual mono- or bidentate P-ligands. Without the costly and environment-burdening P-ligands, this method represented a greener approach. The reaction of aryl halides and >P(O)H reagents, such as dialkyl phosphites and secondary phosphine oxides, was performed using Pd(OAc)_2_ or NiCl_2_ as catalyst precursors in combination with some excess of the P-reagent [14,15]. The trivalent tautomeric form (>P–OH) of the >P(O)H species acted as the ligand to Pd(0) [14]. The mechanisms of these “P-ligand-free” Hirao reactions were explored by high-level quantum chemical calculations [14]. Going further, “ligand-free” Cu-catalyzed protocols were also proposed, and the mechanism of the Cu(I)- and Cu(II)-salt-catalyzed P–C couplings was evaluated [9].

In this article, we report a Pd(OAc)_2_-catalyzed “P-ligand-free” P–C coupling reaction with the less reactive chlorobenzene and its substituted derivatives. These compounds are more advantageous as, compared with the other halogeno- (iodo- or bromo-)arenes, they are cheaper, and with them, the P–C couplings are of a higher atomic efficiency.

## 2. Results and Discussion

### 2.1. Preparative Results

In this study, the P–C coupling of cholorobenzene with diphenylphosphine oxide was chosen as the basic model of MW-assisted “P-ligand-free” Pd-catalyzed coupling. Up until now, not much has been known about the P–C coupling of chlorobenzenes with >P(O)H reagents. In most cases, 10 mol% of Pd(OAc)_2_ was applied as the catalyst precursor using different bases and acetonitrile or ethanol as the solvent. The P-reagent was applied in a 1.3 equivalent quantity (Table 1). The crude reaction mixtures were analyzed by ^31^P NMR and LC-MS.

In the presence of triethylamine in acetonitrile, there was no reaction after a 1.5 h irradiation at 150 °C (Table 1, Entry 1). The next run carried out similarly, but in ethanol also remained, without an appreciable result (Table 1, Entry 2). The Pd(PPh_3_)_4_ catalyst was also tried, and led to a conversion of 39% (Table 1, Entry 3). Substituting it for diisopropyl-ethylamine (the Hünig’s base), there was no reaction at 150 °C in acetonitrile (Table 1, Entry 4). However, in ethanol, a low useful conversion of 8% could be detected (Table 1, Entry 5). In the next experiments, we applied Cs_2_CO_3_ as the base. Irradiating the reaction components in acetonitrile at 120 °C for 1.5 h, the conversion was only 30% (Table 1, Entry 6), but, when repeating the coupling at 135 °C, no chlorobenzene remained in the mixture and the reaction provided triphenylphosphine oxide (**1**) in a complete conversion (Table 1, Entry 7). The special effect of MWs was proved by a comparative thermal reaction that was performed similarly (135 °C/1.5 h) and that led only to a 65% conversion (Table 1, Entry 8). Completion of the reaction required 3 h (Table 1, Entry 9). Carrying out the MW-assisted version in ethanol instead of acetonitrile, the selectivity was found to be somewhat lower (Table 1, Entry 10). In the case, where acetonitrile was used as the solvent, K_2_CO_3_ was as suitable as Cs_2_CO_3_ (Table 1, Entry 11), but using ethanol, the conversion was lower (Table 1, Entry 12).

After the study on coupling with diphenylphosphine oxide, the reaction of chlorobenzene with different >P(O)H reagents, such as diaryl phosphine oxides, diethyl phosphite, and ethyl phenyl-*H*-phosphinate, was investigated using different base/solvent combinations. First, we applied bis(4-methylphenyl)phosphine oxide and bis(3,5-dimethylphenyl)phosphine oxide as the reagents, together with Cs*_2_*CO_3_ as the base, in MeCN at 135 °C. These couplings were not as clear-cut as that obtained using diphenylphosphine oxide (Table 2, Entry 1), as the corresponding triarylphosphine oxides were also formed. The useful conversion for products **2a** and **2b** was 65% and 77%, respectively (Table 2, Entries 2 and 3).

Using diethyl phosphite instead, we found that the use of Cs_2_CO_3_ in acetonitrile was not as efficient as it was in the presence of the secondary phosphine oxides at 150 °C/1 h, as there was no product formed in the mixture (Table 2, Entry 4). The combination of K_2_CO_3_ in acetonitrile was not useful either (Table 2, Entry 5). However, when applying either of the alkali carbonates in ethanol, the phosphinoylation was successful (Table 2 Entries 6 and 7). Using K_2_CO_3_ in ethanol, the coupling occurred in a clear-cut manner, and phosphonate **2c** was formed in a complete conversion (Table 2, Entry 7). Repeating the previous reaction under thermal conditions, the useful conversion was only 65% (Table 2, Entry 8). This shows the beneficial effect of MWs.

The next >P(O)H reagent, ethyl phenyl-*H*-phosphinate, acted similarly to (EtO)_2_P(O)H. There was no reaction in acetonitrile when applying any of the alkali carbonates (Table 2 Entries 9 and 10). To get the ethyl diphenylphosphinate (**2d**), there was need for ethanol ethanol needed to be used as the solvent at 150 °C (Table 2 Entries 11 and 12). In this instance, ~29% of (EtO)_2_PhPO, as the by-product, also appeared in the crude mixture. Ethyl diphenylphosphinate (**2d**) was isolated in 59/55% yields.

Then, a series of substituted chlorobenzenes was reacted with diphenylphosphine oxide under the best conditions found above, applying Cs_2_CO_3_ in MeCN at 135 °C (Table 3). The coupling of the 4-methyl- and 3-methyl-chlorobenzene was complete after irradiation for 1.5 h at 135 °C. The proportion of the expected products (**3e** and **3f**) was 75/63%, while that of the triphenylphosphine oxide was 25/37%. Methylphenyl-diphenylphosphine oxides **3e** and **3f** were prepared in 63/52% yields (Table 3 Entries 2 and 3).

The reaction of 1,4-dichlorobenzene with 1.3 equivalents of Ph_2_P(O)H at 135 °C for 1.5 h afforded not just 4-chlorophenyl-diphenylphosphine oxide (**3g**), but 1,4-bis(diphenylphosphinoyl)benzene **4g** as well. The crude mixture contained 24% of bisphosphinoyl compound **4g** and 53% of triphenylphosphine oxide, which was probably formed by dechlorination (Table 3, Entry 4). With the use of the 1,3-dichlorobenzene, the outcome of the P–C coupling with diphenylphosphine oxide was somewhat similar, but more selective. After irradiation at 135 °C for 1.5 h, 65% of the 3-chlorophenyl-diphenylphosphine oxide (**3h**), 11% of the bisphosphinoyl derivative (**4h**), and 24% of the triphenylphosphine oxide were present in the mixture (Table 3, Entry 5). The major component (**3h**) was isolated in a yield of 49%.

The next derivative was 4-bromochlorobenzene. In this reaction, not just the monophosphinoyl derivative (**3g**), but also 9% of the bisphosphinoyl derivative, was formed, as well as 60% of the triphenylphosphine oxide (Table 3, Entry 6).

Starting from 3-bromo-chlorobenzene, after a 1.5 h reaction time, 90% of the 3-chlorophenyl-diphenylphosphine oxide (**3h**), 3% of the bisphosphinoyl derivative (**4h**), and 7% of the triphenylphosphine oxide were present in the reaction mixture (Table 3, Entry 7). The coupling of the bromo-chlorobenzenes was more selective than that of the dicholorobenzenes.

Additional model compounds applied were 4-chloro methylbenzoate and 4- and 3-chloroacetophenone. Their reaction with Ph_2_P(O)H took place smoothly under the conditions applied above to furnish products **3i**, **3j**, and **3k** in yields of 55%, 59%, and 50%, respectively (Table 3 Entries 8, 9 and 10). Compounds **1**, **2a**–**d**, **3e, 3f**, and **3h**–**k** were all described previously; nevertheless, they were fully characterized by us.

It can be seen that, under suitable conditions, the less reactive chloroarenes may also be suitable starting materials instead of the more expensive bromoarenes. Earlier, only hetaryl chloro-substrates were used in P–C couplings. The *N*-heteroatom activates the starting chloroarenes.

It may be concluded that, under appropriate conditions, the less reactive chloroarenes may also be involved in the Hirao cross-coupling reaction with >P(O)H reagents. The more practical and “greener” couplings may tolerate a wide substrate scope regarding the substituent of the chloroarene.

### 2.2. Theoretical Calculations on the Energetics and Mechanism

The mechanism of the P–C coupling between bromobenzene with diphenylphosphine oxide was calculated by us earlier using the B3LYP/genecp//PCM(MeCN) method [14]. The mechanisms for the reaction of PhX (X=Br or Cl) with Ph_2_P(O)H have now been recalculated at the M06-2X/6-31G(d,p)[PCM(MeCN)] level of theory [18], and the calculations were also extended to the reaction of chlorobenzene. The usual four steps of the catalytic cycle are the oxidative addition (A), the change of the ligands (B), the rearrangement (C), and, finally, the reductive elimination (D).

**Figure 1:**
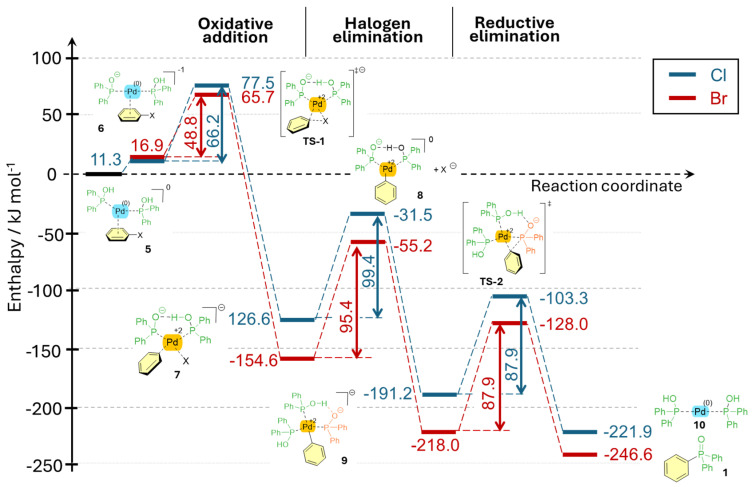
Mechanism for the PhX + Ph_2_P(O)H coupling in the presence of K_2_CO_3_ at the M06-2X/6-31G(d,p)[PCM(MeCN)] level of theory.

In the first step, the Pd(0) formed from Pd(II) on reduction with the slight excess of Ph_2_P(O)H is complexed by two units of the tautomeric form (>P-OH) of Ph_2_P(O)H. Then, Pd(0) coordinates to the Ph ring of the halobenzene, resulting in a weak π-complex (**5**) (Figure 1). Then, as NEt_3_ is a too-weak base, K_2_CO_3_ is needed to deprotonate the π-complex, and leads to species **6**. Its central Pd(0) atom is inserted into the carbon–halogen bond via TS1, resulting in a stable sigma-complex (**7**); meanwhile, the Pd(0) is oxidized to Pd(II) (oxidative addition step). In the subsequent step, an additional Ph_2_POH unit substitutes the leaving halogen anion to afford intermediate **9**, then the phenyl anion attacks the negative P atom via TS2, resulting in the formation of the final Ph_3_PO product (**1**), which leaves the complex in the reductive elimination fashion, and the regeneration of the starting bidental complex (**10**). One can see that the enthalpy profile belonging to the reaction of PhCl (blue lines in Figure 1) runs significantly higher than that for the coupling of PhBr (red lines in Figure 1). Regarding TS1, the enthalpy difference is *ca* 12 kJ mol^−1^, resulting in an almost two-order-of-magnitude difference in the reaction rates between the transformations of PhCl and PhBr.

The enthalpy, Gibbs free energy, and entropy values are listed in Table 4.

**Figure 2:**
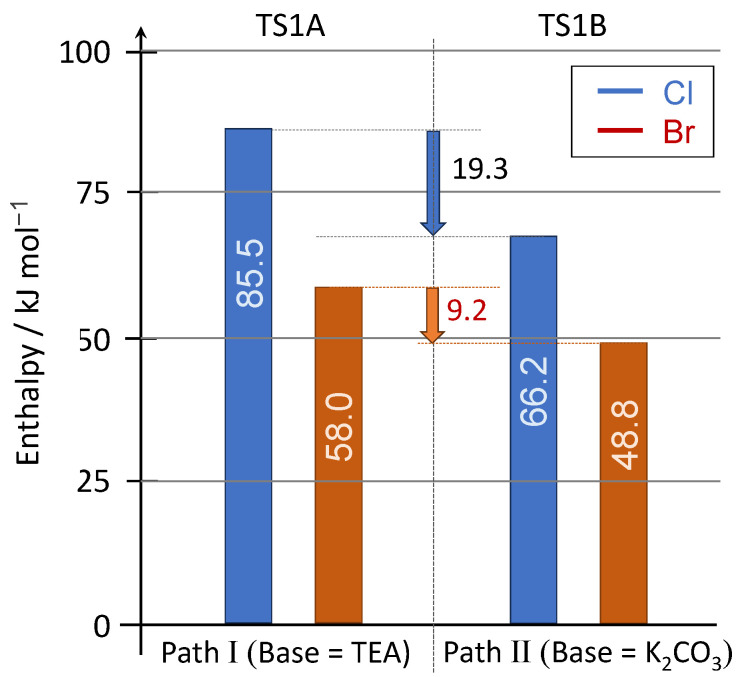
Enthalpy of activation values calculated by the M06-2X/6-31G(d,p)[PCM(MeCN)] level of theory for the coupling of Ph_2_P(O)H with PhCl and PhBr in the presence of triethylamine or K_2_CO_3_ in MeCN.

Analyzing the oxidative addition in a more detailed way, it was found that, in the NEt_3_-catalyzed coupling of PhBr and PhCl, the enthalpy of activation was 58.0 kJ mol^−1^ and 85.5 kJ mol^−1^, respectively (Figure 2). The difference of 27.5 kJ mol^−1^ clearly shows the lower reactivity of the chloroarene. Applying K_2_CO_3_ as the base, the activation barrier for the substitution of PhBr and PhCl was found to be 48.8 kJ mol^−1^ and 66.2 kJ mol^−1^, respectively. On the one hand, the tendency was the same as in the previous case, while, on the other hand, the difference between the two values of enthalpy of activation was smaller (17.4 kJ mol^−1^). Regarding the two bases that were applied, for PhCl, the difference was 19.3 kJ mol^−1^, while for PhBr, it was 9.2 kJ mol^−1^. These results are in accord with the outcome that the attempted coupling of PhCl and Ph_2_P(O)H did not take place when NEt_3_ was applied. The enthalpy of activation for the PhBr + Ph_2_P(O)H coupling using NEt_3_ was somewhat similar than that of the barrier for the PhCl + Ph_2_(O)H reaction in the presence of K_2_CO_3_ (58 vs. 66 kJ mol^−1^).

## 3. Experimental

### 3.1. General Information

The reactions were carried out in a CEM^®^ Discover Model SP (300 W) focused microwave reactor (CEM Microwave Technology Ltd., Buckingham, UK) equipped with a stirrer and a pressure controller using 80–100 W irradiation under isothermal conditions. The reaction mixtures were irradiated in sealed glass vessels (with a volume of 10 mL) available from CEM^®^. The reaction temperature was monitored by an external IR sensor.

The ^31^P, ^13^C, and ^1^H NMR spectra were taken on a Bruker Avance 300/Avance 500 spectrometer (Rheinstetten, Germany) operating at 121.5/202.4, 75.5/125.7, and 300/500 MHz, respectively, in CDCl_3_ solution. The ^31^P chemical shifts are downfield relative to H_3_PO_4_, while the ^13^C and ^1^H chemical shifts are downfield relative to TMS. The couplings are given in Hz. The exact mass measurements were performed using an Agilent 6545 Q-TOF mass spectrometer (Santa Clara, CA, USA) in high resolution, positive electrospray mode. Preparative HPLC purifications, applied in a few cases (where marked), were carried out on a Teledyne ACCQPrep HP150 instrument (Teledyne Technologies Incorporated, Thousand Oaks, CA, USA) using a Phenomenex Gemini C18 (Torrance, CA 90501-1430, USA) 250 × 50.00 mm; 10 μm, 110 Å column. The flow speed was set to 120 mL min^−1^ and the elution programs were usually around 30 min when using 0.1% *v*/*v* TFA in water (A) and MeCN (B).

### 3.2. Best Procedures for the P–C Coupling of Chlorobenzene and >P(O)H-Reagents (Table 2)

To 0.038 mmol (0.0085 g) of Pd(OAc)_2_, we added 0.38 mmol (0.039 mL) of chlorobenzene, 0.49 mmol of the >P(O)H reagents (diphenylphosphine oxide: 0.10 g; bis(4-methylphenyl)phosphine oxide: 0.11 g; bis(3,5-dimethylphenyl)phosphine oxide: 0.13 g; diethyl phosphite: 0.064 mL, ethyl phenyl *H*-phosphinate: 0.075 mL), 0.42 mmol of different bases (cesium carbonate: 0.14 g or potassium carbonate: 0.058 g), and 1 mL of ethanol or acetonitrile solvent. Then, the resulting mixture was irradiated isothermally in a closed vial in the MW reactor at 135 or 150 °C (as shown in Table 1 and Table 2). The reaction mixture was diluted with 3 mL of EtOH or MeCN, then filtrated, and the residue obtained after evaporation of the filtrate was filtered through a thin (2–3 cm) layer of silica gel using ethyl acetate as the eluant. The crude mixture obtained was analyzed by ^31^P NMR spectroscopy. Then, if the sample was relevant, it was purified further by column chromatography (silica gel, ethyl acetate–hexane 1:1 as the eluant) to afford products **1**, **2a**–**d**.

The following compounds were thus prepared:

#### 3.2.1. Triphenylphosphine Oxide (**1**) (Table 2, Entry 1)

Yield: 0.086 g (81%) obtained as white crystals; mp. 156–157 °C, mp. lit. [17] 156.6–157.4 °C; ^31^P{^1^H} NMR (121.5 MHz, CDCl_3_) δ 29.1, δ_P_ lit. [17] (162 MHz, CDCl_3_) 29.5; ^13^C{^1^H} NMR (75.5 MHz, CDCl_3_) δ 132.7 (d, *J* = 103.8 Hz), 132.2 (d, *J* = 9.9 Hz,), 132.0 (d, *J* = 2.8 Hz), 128.6 (d, *J* = 12.1 Hz), δ_C_ lit. [17] (100 MHz, CDCl_3_) 132.8 (d, *J* = 104.6 Hz), 132.5 (d, *J* = 9.9 Hz), 131.9 (d, *J* = 2.2 Hz), 128.4 (d, *J* = 12.1 Hz); ^1^H NMR (300 MHz, CDCl_3_) δ 7.72–7.59 (m, 6H), 7.56–7.48 (m, 3H), 7.48–7.38 (m, 6H), δ_H_ lit. [17] (400 MHz, CDCl_3_) 7.70–7.64 (m, 6H), 7.56–7.52 (m, 3H), 7.48–7.43 (m, 6H); HRMS (*m*/*z*): calcd for C_18_H_16_OP [M + H]^+^ = 279.0939; found, 279.0941.

#### 3.2.2. Bis(4-Methylphenyl)-Phenylphosphine Oxide (**2a**) (Table 2, Entry 2)

Yield: 0.067 g (58%) obtained as white crystals; mp. 76 °C, mp. lit. [15] 78–79 °C; ^31^P{^1^H} NMR (121.5 MHz, CDCl_3_) δ 27.8, δ_P_ lit. [19] (162 MHz, CDCl_3_) 30.5; ^13^C{^1^H} NMR (75.5 MHz, CDCl_3_) δ 142.4 (d, *J* = 2.8 Hz), 133.1 (d, *J* = 104.1 Hz), 132.1 (d, *J* = 10.3 Hz), 132.1 (d, *J* = 9.8 Hz), 131.8 (d, *J* = 2.7 Hz), 129.4 (d, *J* = 106.6 Hz), 129.3 (d, *J* = 12.5 Hz), 128.5 (d, *J* = 12.1 Hz), 21.6 (s), δ_C_ lit [19]. (100 MHz, CDCl_3_) 142.6 (d, *J* = 2.9 Hz), 133.0 (d, *J* = 102.5 Hz), 132.2 (d, *J* = 10.2 Hz), 132.0 (d, *J* = 8.7 Hz), 131.9 (d, *J* = 3.2 Hz), 129.4 (d, *J* = 106.9 Hz), 129.4 (d, *J* = 12.6 Hz), 128.6 (d, *J* = 11.8 Hz), 21.7; ^1^H NMR (300 MHz, CDCl_3_) δ 7.73–7.61 (m, 2H), 7.61–7.47 (m, 5H), 7.47–7.37 (m, 2H), 7.32–7.18 (m, 4H), 2.39 (s, 6H); δ_H_ lit [19] (400 MHz, CDCl_3_) 7.68–7.62 (m, 2H), 7.53 (dd, *J*_1_ = 11.8 Hz, *J*_2_ = 8.0 Hz, 4H), 7.48 (m, 1H), 7.24 (dd, *J*_1_ = 8.4 Hz, *J*_2_ = 2.4 Hz, 4H), 2.38 (s, 6H); HRMS (*m*/*z*): calcd for C_20_H_20_OP [M + H]^+^ = 307.1252; found, 307.1252.

#### 3.2.3. Bis(3,5-Dimethylphenyl)-Phenylphosphine Oxide (**2b**) (Table 2, Entry 3)

Yield: 0.084 g (66%) obtained as white crystals; mp. 159 °C, mp. lit [19]. 158.6–159.2 °C; ^31^P{^1^H} NMR (121.5 MHz, CDCl_3_): δ 29.6, δ_P_ lit [19]. (162 MHz, CDCl_3_) 30.9; ^13^C{^1^H} NMR (75.5 MHz, CDCl_3_) δ 138.1 (d, *J* = 12.7 Hz), 133.7 (d, *J* = 2.8 Hz), 133.1 (d, *J* = 103.1 Hz), 132.4 (d, *J* = 105.3 Hz), 132.1 (d, *J* = 9.9 Hz), 131.7, 129.7 (d, *J* = 9.8 Hz), 128.4 (d, *J* = 12.0 Hz), 21.4 (s), δ_C_ lit [19]. (100 MHz, CDCl_3_) 138.3 (d, *J* = 12.2 Hz), 133.9 (d, *J* = 2.3 Hz), 133.1 (d, *J* = 102.7 Hz), 132.4 (d, *J* = 102.6 Hz), 132.3 (d, *J* = 9.7 Hz), 131.9 (d, *J* = 2.2 Hz), 129.8 (d, *J* = 10.0 Hz), 128.6 (d, *J* = 11.7 Hz), 21.56; ^1^H NMR (300 MHz, CDCl_3_) δ 7.73–7.62 (m, 2H), 7.55–7.39 (m, 3H), 7.28 (d, *J* = 12.2 Hz, 4H), 7.15 (s, 2H), 2.31 (s, 12H), δ_H_ lit [19]. (400 MHz, CDCl_3_) 7.68–7.63 (m, 2H), 7.55–7.51 (m, 1H), 7.47–7.42 (m, 2H), 7.26 (d, *J* = 12.4 Hz, 4H), 7.15 (s, 2H), 2.31 (s, 12H); HRMS (*m*/*z*): calcd for C_22_H_24_OP [M + H]^+^ = 335.1565; found, 335.1566.

#### 3.2.4. Diethyl Phenylphosphonate (**2c**) (Table 2, Entry 7)

Yield: 0.058 g (71%) obtained as colorless oil; ^31^P{^1^H} NMR (121.5 MHz, CDCl_3_) δ 18.9, δ_P_ lit [7]. (162 MHz, CDCl_3_) 18.8; ^13^C{^1^H} NMR (75.5 MHz, CDCl_3_) δ 132.5 (d, *J* = 3.0 Hz), 131.9 (d, *J* = 9.8 Hz), 128.6 (d, *J* = 15.0 Hz), 128.5 (d, *J* = 188.0 Hz), 62.2 (d, *J* = 5.4 Hz), 16.4 (d, *J* = 6.5 Hz), δ_C_ lit [7]. (100 MHz, CDCl_3_) 132.3 (d, *J* = 2.7 Hz), 131.7 (d, *J* = 9.2 Hz), 128.44 (d, *J* = 15.2 Hz), 128.41 (d, *J* = 187.6 Hz), 62.0 (d, *J* = 5.9 Hz), 16.3 (d, *J* = 6.5 Hz); ^1^H NMR (300 MHz, CDCl_3_) δ 7.84–7.71 (m, 2H), 7.56–7.37 (m, 3H), 4.21–3.97 (m, 4H), 1.29 (t, *J* = 7.1 Hz, 6H), δ_H_ lit [7]. (400 MHz, CDCl_3_) 7.82 (m, 2H), 7.55 (~tq, *J*_1_ = 7.5 Hz, *J*_2_ = 1.4 Hz, 1H), 7.47 (m, 2H), 4.12 (m, 4H), 1.32 (td, *J*_1_ *=* 7.0 Hz, *J*_2_ = 0.5 Hz, 6H); HRMS (*m*/*z*): calcd for C_10_H_16_O_3_P [M + H]^+^ = 215.0837; found, 215.0835.

#### 3.2.5. Ethyl Diphenylphosphinate (**2d**) (Table 2, Entry 11)

Yield: 0.055 g (59%) obtained as colorless oil; ^31^P{^1^H} NMR (121.5 MHz, CDCl_3_) δ 32.2, δ_P_ lit [20]. (120 MHz, CDCl_3_) 30.8; ^13^C{^1^H} NMR (75.5 MHz, CDCl_3_) δ 132.0 (d, *J* = 2.8 Hz), 131.7 (d, *J* = 137.0 Hz), 131.6 (d, *J* = 10.1 Hz), 128.4 (d, *J* = 13.1 Hz), 61.1 (d, *J* = 5.9 Hz), 16.5 (d, *J* = 6.6 Hz), δ_C_ lit [20]. (75 MHz, CDCl_3_) 139.9 (d, *J* = 11.1 Hz), 133.7 (d, *J* = 143.4 Hz), 130.9 (d, *J* = 12.9 Hz), 127.0, 59.0, 16.7; ^1^H NMR (300 MHz, CDCl_3_) δ 7.88–7.75 (m, 4H), 7.58–7.39 (m, 6H), 4.16–4.01 (m, 2H), 1.36 (t, *J* = 7.1 Hz, 3H), δ_H_ lit [20]. (300 MHz, CDCl_3_) 7.70–7.63 (m, 4H), 7.43–7.31 (m, 6H), 4.16–4.09 (m, 2H), 1.30 (t, *J* = 7.3 Hz, 3H); HRMS (*m*/*z*): calcd for C_14_H_16_O_2_P [M + H]^+^ = 247.0888; found, 247.0889.

### 3.3. General Procedure for the P–C Coupling of Substituted Chlorobenzenes and Diphenylphosphine Oxide (Table 3)

To 0.038 mmol (0.0085 g) of Pd(OAc)_2_ in 1 mL of acetonitrile, we, added 0.38 mmol of substituted chlorobenzenes (4-chlorotoluene: 0.045 mL; 3-chlorotoluene: 0.045 mL; 1,4-dichlorobenzene: 0.045 mL; 1,3-dicholorobenzene: 0.043 mL; 4-bromochlorobenzene: 0.073 g; 3-bromochlorobenzene: 0.045 mL, methyl 4-chlorobenzoate: 0.065 g, 4-acetylchlorobenzene: 0.049 mL, 3-acetylchlorobenzene: 0.050 mL), 0.49 mmol (0.10 g) diphenylphosphine oxide, and 0.42 mmol (0.14 g) of cesium carbonate. Then, the resulting mixture was irradiated isothermally in a closed vial in the MW reactor at 135 °C for 1.5 h. The reaction mixture was diluted with 3 mL of MeCN, then filtrated, and the residue obtained after evaporation of the filtrate was filtered through a thin (2–3 cm) layer of silica gel using ethyl acetate as the eluant. The crude mixture obtained was analyzed by ^31^P NMR spectroscopy. Then, if the sample was relevant, it was purified further by column chromatography (silica gel, ethyl acetate–hexane 1:1 as the eluant) to afford products **3e**, **3f**, and **3h**. Compounds **3i**–**k** were purified by preparative HPLC.

The following compounds were thus prepared:

#### 3.3.1. 4-Methylphenyl-Diphenylphosphine Oxide (**3e**) (Table 3, Entry 2)

Yield: 0.069 g (63%) obtained as a white solid; mp 118–119 °C, mp. lit [21]. 129.5–130.2 °C; ^31^P{1H} NMR (121.5 MHz, CDCl_3_) δ 29.3, δ_P_ lit [21]. (162 MHz, CDCl_3_) 29.1; ^13^C{1H} NMR (125.7 MHz, CDCl_3_) δ 142.4 (d, *J* = 2.8 Hz), 132.8 (d, *J* = 105.9 Hz), 132.1 (d, *J* = 10.2 Hz), 132.0 (d, *J* = 9.9 Hz), 131.8 (d, *J* = 2.7 Hz), 129.2 (d, *J* = 12.6 Hz), 129.1 (d, *J* = 106.4 Hz), 128.4 (d, *J* = 12.1 Hz), 21.6 (s), δ_C_ lit [21]. (100 MHz, CDCl_3_) 142.5 (d, *J* = 2.6 Hz), 132.9 (d, *J* = 104.1 Hz), 132.2 (d, *J* = 10.2 Hz), 132.1 (d, *J* = 10.0 Hz), 131.9 (d, *J* = 2.8 Hz), 129.3 (d, *J* = 12.5 Hz), 129.2 (d, *J* = 106.4 Hz), 128.5 (d, *J* = 11.9 Hz), 21.6; ^1^H NMR (500 MHz, CDCl_3_) δ 7.80–7.40 (m, 12H), 7.29–7.11 (m, 2H), 2.40 (s, 3H), δ_H_ lit [21]. (400 MHz, CDCl_3_) 7.67–7.62 (m, 4H), 7.56–7.48 (m, 4H), 7.44–7.40 (m, 4H), 7.26–7.23 (m, 2H), 2.37 (s, 3H); HRMS (*m*/*z*): calcd for C_19_H_18_OP [M + H]+ = 293.1095; found, 293.1101.

#### 3.3.2. 3-Methylphenyl-Diphenylphosphine Oxide (**3f**): (Table 3, Entry 3)

Yield: 0.058 g (52%) obtained as a white solid; mp. 122–123 °C, mp. Lit [19]. 123.7–124.2 °C; ^31^P{^1^H} NMR (202.4 MHz, CDCl_3_) δ 29.3, δ_P_ lit [22]. (162 MHz, CDCl_3_) 29.5; ^13^C{^1^H} NMR (125.7 MHz, CDCl_3_) δ 138.5 (d, *J* = 12.1 Hz), 132.8 (d, *J* = 3.0 Hz), 132.7 (d, *J* = 104.0 Hz), 132.5 (d, *J* = 9.5 Hz), 132.3 (d, *J* = 96.7 Hz), 132.1 (d, *J* = 9.9 Hz), 131.9 (d, *J* = 2.6 Hz), 129.2 (d, *J* = 10.2 Hz), 128.5 (d, *J* = 12.0 Hz), 128.3 (d, *J* = 12.9 Hz), 21.4 (s), δ_C_ lit [22]. (100 MHz, CDCl_3_) 138.4 (d, *J* = 15.9 Hz), 133.1, 132.8 (d, *J* = 2.4 Hz), 132.5 (d, *J* = 9.5 Hz), 132.2 (d, *J* = 103.4 Hz), 132.0 (d, *J* = 9.8 Hz), 131.8 (d, *J* = 2.5 Hz), 129.2 (d, *J* = 10.2 Hz), 128.5 (d, *J* = 12.1 Hz), 128.2, 21.5; ^1^H NMR (500 MHz, CDCl_3_) δ 7.72–7.67 (m, 4H), 7.61–7.55 (m, 3H), 7.50–7.47 (m, 4H), 7.42–7.34 (m, 3H), 2.39 (s, 3H), δ_H_ lit [22]. (400 MHz, CDCl_3_) 7.64 (dd, *J*_1_ = 11.6 Hz, *J*_2_ = 7.6 Hz, 4H), 7.56–7.49 (m, 3H), 7.44–7.43 (m, 4H), 7.32 (m, 3H), 2.33 (s, 3H); HRMS (*m*/*z*): calcd for C_19_H_18_OP [M + H]^+^ = 293.1095; found, 293.1097.

#### 3.3.3. 3-Chlorophenyl-Diphenylphosphine Oxide (**3h**): (Table 3, Entry 7)

Yield: 0.099 g (83%) obtained as white crystals, mp. 75–76 °C; ^31^P{^1^H} NMR (202.4 MHz, CDCl_3_) δ 28.1 ^31^P{^1^H} NMR (202.4 MHz, CDCl_3_) δ 28.1; ^13^C{^1^H} NMR (125.7 MHz, CDCl_3_) δ 135.1 (d, *J* = 101.3 Hz), 135.0 (d, *J* = 15.6 Hz), 132.3 (d, *J* = 2.8 Hz), 132.1 (d, *J* = 2.7 Hz), 132.0 (d, *J* = 10.0 Hz)^a^, 131.84 (d, *J* = 10.7 Hz)^b^, 131.82 (d, *J* = 105.1 Hz), 130.1 (d, *J* = 9.5 Hz)^b^, 129.9 (d, *J* = 12.9 Hz), 128.7 (d, *J* = 12.3 Hz)^a^, ^a,b^ may be reversed; ^1^H NMR (500 MHz, CDCl_3_) δ 7.68–7.64 (m, 5H), 7.59–7.55 (m, 3H), 7.53–7.47 (m, 5H), 7.43–7.39 (m, 1H); HRMS (*m*/*z*): calcd for C_18_H_15_OPCl [M + H]^+^ = 313.0549; found 313.0547.

#### 3.3.4. (4-Methoxycarbonylphenyl)-Diphenylphosphine Oxide (**3i**): (Table 3, Entry 8)

Yield: 0.070 g (55%) obtained as colorless oil; ^31^P{^1^H} NMR (202.4 MHz, CDCl_3_) δ 29.7, δ_P_ lit [23]. (162 MHz, CDCl_3_) δ 28.2; ^13^C{^1^H} NMR (125.7 MHz, CDCl_3_) δ 166.1 (s), 137.1 (d, *J* = 101.5 Hz), 133.3 (d, *J* =2.8 Hz), 132.4 (d, *J* = 2.8 Hz), 132.1 (d, *J* = 10.1 Hz)^a^, 132.0 (d, *J* = 10.1 Hz)^b^, 131.3 (d, *J* = 105.3 Hz), 129.5 (d, *J* = 12.2 Hz)^a^, 128.7 (d, *J* = 12.3 Hz)^b^, 52.5 (s), ^a,b^ may be reversed δ_C_ lit [23].(150 MHz, CDCl_3_) δ 166.08, 137.56, 133.07, 132.12, 132.02, 131.91, 131.39, 129.29, 128.54, 52.33; ^1^H NMR (500 MHz, CDCl_3_) δ 8.14–8.12 (m, 2H), 7.79–7.75 (m, 2H), 7.69–7.64 (m, 4H), 7.61–7.57 (m, 2H), 7.52–7.48 (m, 4H), 3.95 (s, 3H), δ_H_ lit [23]. (600 MHz, CDCl_3_) δ 8.12–8.10 (m, 2H), 7.78–7.75 (m, 2H), 7.68–7.65 (m, 4H), 7.58–7.56 (m, 2H), 7.50–7.47 (m, 4H), 3.94 (s, 3H); HRMS (*m*/*z*): calcd for C_20_H_18_O_3_P [M + H]^+^ = 337.0993; found 337.0994.

#### 3.3.5. (4-Acetylphenyl)-Diphenylphosphine Oxide(**3j**): (Table 3, Entry 9)

Yield: 0.072 g (59%) obtained as colorless oil; ^31^P{^1^H} NMR (202.4 MHz, CDCl_3_) δ 31.0 δ_P_ lit [23]. (162 MHz, CDCl_3_) δ 28.0; ^13^C{^1^H} NMR (125.7 MHz, CDCl_3_) δ 197.4 (s), 139.8 (d, *J* = 2.7 Hz), 136.5 (d, *J* =102.3 Hz), 132.7 (d, *J* = 2.8 Hz), 132.5 (d, *J* = 10.2 Hz)^a^, 132.1 (d, *J* = 9.9 Hz)^b^, 130.6 (d, *J* = 106.3 Hz), 128.8 (d, *J* = 12.7 Hz)^b^, 128.2 (d, *J* = 12.3 Hz)^a^, 26.8 (s), ^a,b^ may be reversed δ_C_ lit [23]. (100 MHz, CDCl_3_) 197.38, 139.37, 137.61, 132.29, 132.17, 131.89, 131.13, 128.56, 127.92, 26.69; ^1^H NMR (500 MHz, CDCl_3_) δ 8.07–8.05 (m, 2H), 7.84–7.80 (m, 2H), 7.70–7.66 (m, 4H), 7.64–7.61 (m, 2H), 7.54–7.51 (m, 4H), 2.66 (s, 3H), δ_H_ lit [23]. (400 MHz, CDCl_3_) 8.03–8.01 (m, 2H), 7.82–7.77 (m, 2H), 7.69–7.64 (m, 4H), 7.59–7.55 (m, 2H), 7.50–7.46 (m, 4H), 2.63 (s, 3H); HRMS (*m*/*z*): calcd for C_20_H_18_O_2_P [M + H]^+^ = 321.1044; found 321.1046.

#### 3.3.6. (3-Acetylphenyl)-Diphenylphosphine Oxide (**3k**): (Table 3, Entry 10)

Yield: 0.061 g (50%) obtained as colorless oil; ^31^P{^1^H} NMR (202.4 MHz, CDCl_3_) δ 31.4 δ_P_ lit [23]. (162 MHz, CDCl_3_) δ 28.3; ^13^C{^1^H} NMR (125.7 MHz, CDCl_3_) δ 197.1 (s), 137.3 (d, *J* = 11.1 Hz), 136.3 (d, *J* =10.6 Hz)^a^, 132.7 (d, *J* = 2.8 Hz), 132.3 (d, *J* = 104.6 Hz), 132.1 (d, *J* = 10.3 Hz)^b^, 131.89 (d, *J* = 3.2 Hz), 131.87 (d, *J* = 10.2 Hz)^a^, 130.5 (d, *J* = 106.4 Hz), 129.1 (d, *J* = 12.2 Hz)^a^, 128.9 (d, *J* = 12.4 Hz)^b^, 26.6 (s), ^a,b^ may be reversed, δ_C_ lit [23].(150 MHz, CDCl_3_) 197.16, 137.17, 136.23, 133.62, 132.22, 131.98, 131.82, 131.55, 131.40, 128.87, 128.64, 26.64; ^1^H NMR (500 MHz, CDCl_3_) δ 8.24–8.21 (m, 1H), 8.11–8.08 (m, 1H), 7.80–7.76 (m, 1H), 7.62–7.52 (m, 7H), 7.46–7.42 (m, 4H), 2.53 (s, 3H), δ_H_ lit [23]. (600 MHz, CDCl_3_) 8.31 (d, *J* = 12.0 Hz, 1H), 8.14 (d, *J* = 7.8 Hz, 1H), 7.86–7.82 (m, 1H), 7.69–7.65 (m, 4H), 7.59–7.56 (m, 3H), 7.50–7.47 (m, 4H), 2.59 (s, 3H); HRMS (*m*/*z*): calcd for C_20_H_18_O_2_P [M + H]^+^ = 321.1044; found 321.1042.

### 3.4. Details of the Theoretical Calculations 

All computations were carried out with the Gaussian16 program package (G16C1) [24], using standard convergence criteria for the gradients of the root mean square (RMS), force, maximum force, RMS displacement, and maximum displacement vectors (3.0 × 10^−4^, 4.5 × 10^−4^, 1.2 × 10^−3^, and 1.8 × 10^−3^). Computations were carried out at the M06-2X level of theory [25], with a basis set of 6–31G(d,p). The vibrational frequencies were computed at the same level of theory as the small molecular system; however, in the case of the explicit solvent model, M06-2X/6-31G(d,p)[PCM(MeCN)] was applied. The thermodynamic functions U, H, G, and S were computed at 398.15 K. In addition to the vacuum calculations, the IEFPCM method was also applied to model the solvent effect, using the default settings of G16 as the setting for MeCN [26]. See the Supporting Information for details.

## 4. Conclusions

In summary, the P–C coupling of the less reactive chlorobenzene and a few >P(O)H reagents, such as diarylphosphine oxides, diethyl phosphite, and ethyl *H*-phosphinate, was carried out using 10% Pd(OAc)_2_ catalyst precursor and an alkali carbonate (Cs_2_CO_3_ or K_2_CO_3_) in ethanol or in acetonitrile under MW irradiation. A “green” point was that instead of the traditional mono- or bidante P-ligands, the excess (20%) of the >P(O)H reagent was used via its >P-OH trivalent tautomer form. Chlorobenzene could also be involved in Hirao reactions with diethyl phosphite and ethyl phenyl-*H* phosphinate, applying an alkali carbonate in ethanol. Then, a series of substituted chlorobenzenes comprising derivatives with electron-donating and electron-withdrawing substituents was reacted with diphenylphosphine oxide, applying Cs_2_CO_3_ in acetonitrile. The mechanism of the P–C coupling of bromobenzene and chlorobenzene with Ph_2_P(O)H was explored by theoretical calculations, revealing the lesser reactivity of the chloroarene but suggesting that the activation barriers may be overcome. It was also found that the activation enthalpy for the coupling of chlorobenzene and diphenylphosphine oxide in the presence of a carbonate is similar to the phosphinoylation of the more reactive bromobenzene using triethylamine. It can be concluded that, under appropriate conditions, the cheaper chloroarenes may be used instead of the bromo derivatives, allowing a more practical and a “greener” approach.

## Figures and Tables

**Table 1:**
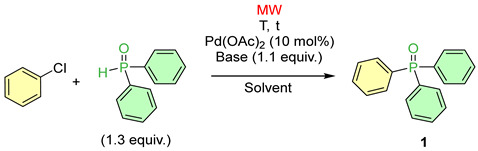
Optimization of the coupling reaction between chlorobenzene and diphenylphosphine oxide.

Entry	Base	Solvent	T (°C)	t (h)	Conversion ^a^ (%)	Product Composition ^a,b^ (%)		Yield of 1 (%)
						1	(EtO)Ph_2_PO	
1	NEt_3_	MeCN	150	1.5	0	–	–	–
2	NEt_3_	EtOH	150	1.5	10	5	5	–
3 ^c^	NEt_3_	MeCN	150	1.5	39	39	–	–
4	N ^i^Pr_2_E	MeCN	150	1.5	0	–	–	–
5	N ^i^Pr_2_E	EtOH	150	1.5	14	8	6	–
6	Cs_2_CO_3_	MeCN	120	1.5	30	30	–	–
7	Cs_2_CO_3_	MeCN	135 ^d^	1.5	100	100	–	81
8	Cs_2_CO_3_	MeCN	135 ^e^	1.5	65	65	–	32
9	Cs_2_CO_3_	MeCN	135 ^e^	3	100	100	–	73
10	Cs_2_CO_3_	EtOH	135	1.5	100	94	6	71
11	K_2_CO_3_	MeCN	135	1.5	100	100	–	73
12	K_2_CO_3_	EtOH	135	1.5	45	32	13	–

^a^ On the basis of relative ^31^P NMR intensities. ^b^ The average of two or three parallel experiments. ^c^ 10 mol% of Pd(PPh_3_)_4_ and 1.0 equiv. of Ph_2_P(O)H reagent. ^d^ A three-fold scale-up led to a yield of 78%. ^e^ On conventional heating.

**Table 2:**
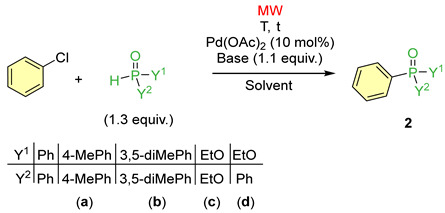
The coupling reaction between chlorobenzene and different >P(O)H reagents.

Entry	Y^1^, Y^2^	Base	Solvent	T (°C)	t (h)	Conversion ^a^ (%)	Product Composition ^a,b^ (%)		Yield (%)
							2	Y_3_P = O or (EtO)_2_PhP(O)	
1	Ph	Cs_2_CO_3_	MeCN	135	1.5	100	100 (**1**)	–	81 (**1**)
2	4-MeC_6_H_4_ (**a**)	Cs_2_CO_3_	MeCN	135	2	100	65	35 ^c^	58 (**2a**)
3	3,5-diMeC_6_H_3_ (**b**)	Cs_2_CO_3_	MeCN	135	2	92	77	15 ^d^	66 (**2b**)
4	EtO (**c**)	Cs_2_CO_3_	MeCN	150	1	0	–	–	–
5	EtO (**c**)	K_2_CO_3_	MeCN	150	1	0	–	–	–
6	EtO (**c**)	Cs_2_CO_3_	EtOH	150	1	92	92	–	61 (**2c**)
7	EtO (**c**)	K_2_CO_3_	EtOH	150	1	100	100	–	71 (**2c**)
8 ^e^	EtO (**c**)	K_2_CO_3_	EtOH	150	1	68	68	–	49 (**2c**)
9	EtO, Ph (**d**)	Cs_2_CO_3_	MeCN	150	1	0	–	–	–
10	EtO, Ph (**d**)	K_2_CO_3_	MeCN	150	1	0	–	–	–
11	EtO, Ph (**d**)	Cs_2_CO_3_	EtOH	150	1	100	73	27 ^f^	59 (**2d**)
12	EtO, Ph (**d**)	K_2_CO_3_	EtOH	150	1	98	67	31 ^f^	55 (**2d**)

^a^ On the basis of relative ^31^P NMR intensities. ^b^ The average of two or three parallel experiments. ^c^ (4-MeC_6_H_4_)_3_PO as the by-product; HRMS (*m*/*z*): calcd for C_21_H_22_OP [M + H]^+^ = 321.1408, found, 321.1406. ^d^ (3,5-diMeC_6_H_3_)_3_PO as the side-product; HRMS (*m*/*z*): calcd for C_24_H_28_OP [M + H]^+^ = 363.1878, found, 363.1876. ^e^ On conventional heating. ^f^ (EtO)_2_PhPO as the side-product of ^31^P NMR (CDCl_3_) δ 18.9, δ_P_ lit. [7] (CDCl_3_) 18.8; HRMS (*m*/*z*): calcd for C_10_H_16_O_3_P [M + H]^+^ = 215.0837; found, 215.0835.

**Table 3:**
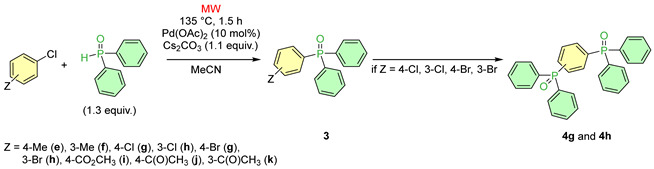
P–C coupling reactions of substituted chlorobenzenes with diphenylphosphine oxide.

Entry	Z	Product Composition ^a,b^ (%)			Yield of 3 (%)
		3	4	Ph_3_PO	
1	H	100	–		81 (**1**)
2	4-Me	75	–	25	63 (**3e**)
3	3-Me	63	–	37	52 (**3f**)
4	4-Cl	23 ^c^	24 ^d^	53	–
5	3-Cl	65	11 ^e^	24	49 (**3h**)
6	4-Br	31^c^	9 ^d^	60	–
7	3-Br	90	3 ^e^	7	83 (**3h**)
8	4-CO_2_CH_3_	87	–	13	55 (**3i**)
9	4-C(O)CH_3_	84	–	16	59 (**3j**)
10	3-C(O)CH_3_	80	–	20	50 (**3k**)

^a^ On the basis of relative ^31^P NMR intensities of the P-components. ^b^ The average of two or three parallel experiments. ^c^ For **3g***:* ^31^P NMR (CDCl_3_) δ 28.5, δ_P_ lit. [16] (CDCl_3_) 28.2; HRMS (*m*/*z*): calcd for C_18_H_15_ClOP [M + H]^+^ = 313.0549; found, 313.0542. ^d^ For **4g**: ^31^P NMR (CDCl_3_) δ 28.6; HRMS (*m*/*z*): calcd for C_30_H_25_O_2_P_2_ [M + H]^+^ = 479.1330; found, 479.1320. ^d,e^ For **4h** ^31^P NMR (CDCl_3_) δ 28.5, δ_P_ lit. [17] (CDCl_3_) 30.5; HRMS (*m*/*z*): calcd for C_30_H_25_O_2_P_2_ [M + H]^+^ = 479.1330; found, 479.1323.

**Table 4:** Enthalpies, Gibbs free energies, and entropies for the mechanism for the PhX + Ph_2_P(O)H coupling in the presence of K_2_CO_3_ at the M06-2X/6-31G(d,p)[PCM(MeCN)] level of theory.

	R = Cl			R = Br		
	Δ*H* (kJ mol^−1^)	Δ*G* (kJ mol^−1^)	Δ*S* (J mol^−1^ K^−1^)	Δ*H* (kJ mol^−1^)	Δ*G* (kJ mol^−1^)	Δ*S* (J mol^−1^ K^−1^)
**5**	0.0	0.0	0.0	0.0	0.0	0.0
**6**	11.3	10.7	1.9	16.9	15.5	4.7
**TS-1**	77.5	79.9	−8.1	65.7	68.5	−9.3
**7**	−126.6	−120.5	−18.7	−154.6	−160.3	19.3
**8**	−31.5	−65.1	112.2	−55.2	−68.4	44.0
**9**	−191.2	−156.4	−115.9	−215.9	−162.0	−179.7
**TS-2**	−103.3	−70.7	−108.6	−128.0	−76.3	−172.4
**1 + 10**	−221.9	−195.7	−87.4	−246.6	−201.3	−151.2

## Data Availability

The data presented in this study are available on request from the corresponding authors.

## Supplementary Materials

The following supporting information can be downloaded at: https://www.mdpi.com/article/10.3390/molecules30051045/s1. The Supporting Information containing ^31^P, ^13^C and ^1^H NMR spectra, as well as data of the theoretical calculations.
