# Supplementary material for: Microwave-Assisted P–C Coupling of the Less Reactive Chlorobenzene and >P(O)H Reagents in the Absence of the Usual Mono- and Bidental P-Ligands
Source: Molecules. 2025 Feb 25;30(5):1045. doi: 10.3390/molecules30051045 (PMC11901695; doi:10.3390/molecules30051045)
Supplement: Supplementary file 1 [file molecules-30-01045-s001.zip › molecules-3397848-supplementary.pdf]

Supporting Information

Microwave-Assisted P–C Coupling of the Less Reactive Chlorobenzene and >P(O)H Reagents in the Absence of the Usual Mono- and Bidental P-Ligands

Bianka Huszár,[a] Zoltán Mucsi[b] and György Keglevich[a,*]

[a]Department of Organic Chemistry and Technology, Faculty of Chemical Technology and Biotechnology, Budapest University of Technology and Economics, 1111 Budapest, Műegyetem rkp. 3., Hungary

[b]Faculty of Materials and Chemical Sciences, University of Miskolc, Miskolc H-3515, Hungary

Table of contents

1. NMR spectra for compounds **1**, **2a-d**, **3e, 3f** and **3h-k** pg. 2–34.
2. Table S1–S4 containing the computed row data pg. 35–37.
3. Additional Tables containing XYZ coordinates of computed species pg. 38–73.

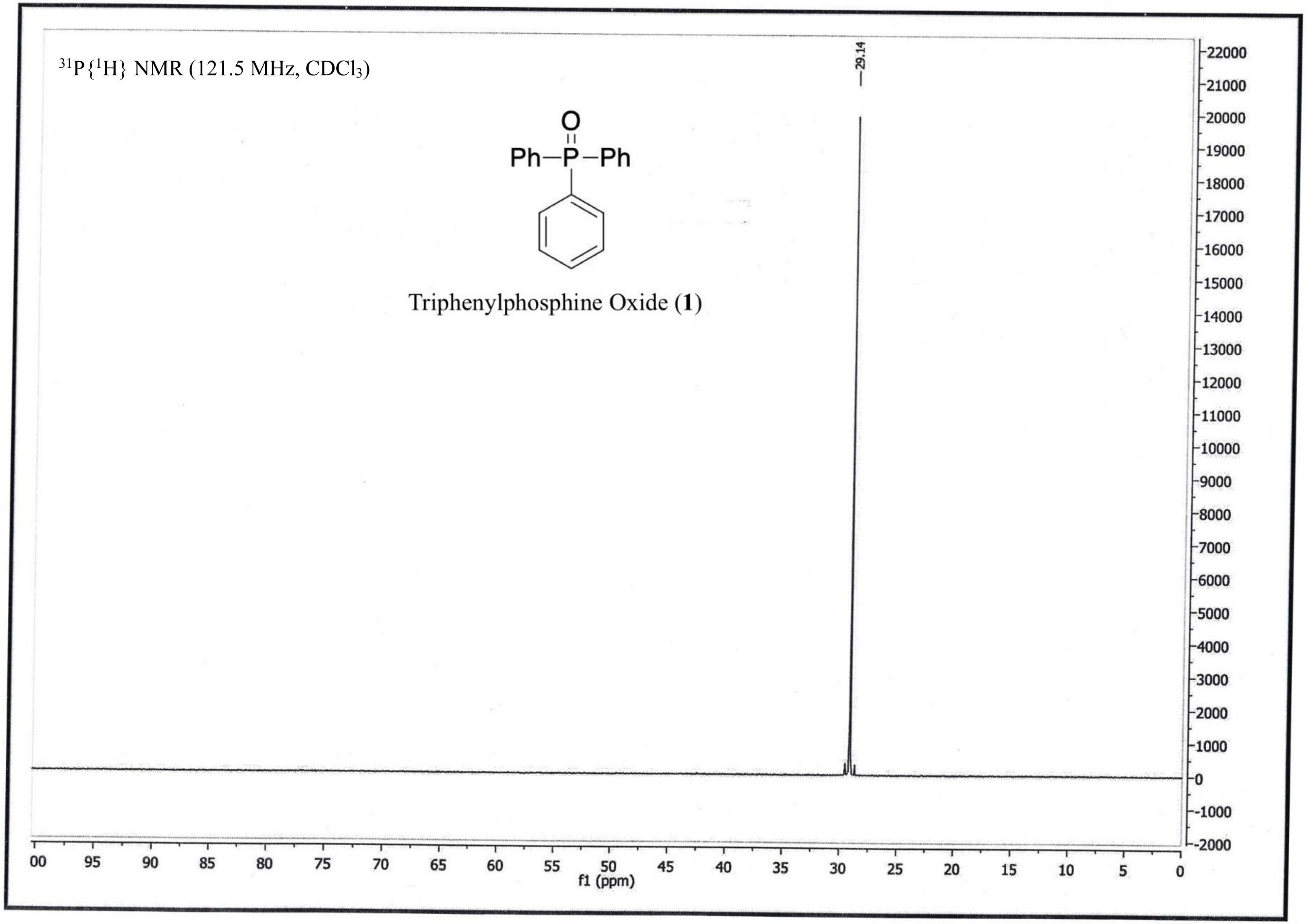
$^{31}P\{^1H\}$ NMR (121.5 MHz, $CDCl_3$)
O
Ph–P–Ph
Triphenylphosphine Oxide (**1**)
29.14
f1 (ppm)
00
95
90
85
80
75
70
65
60
55
50
45
40
35
30
25
20
15
10
5
0
22000
21000
20000
19000
18000
17000
16000
15000
14000
13000
12000
11000
10000
9000
8000
7000
6000
5000
4000
3000
2000
1000
0
-1000
-2000

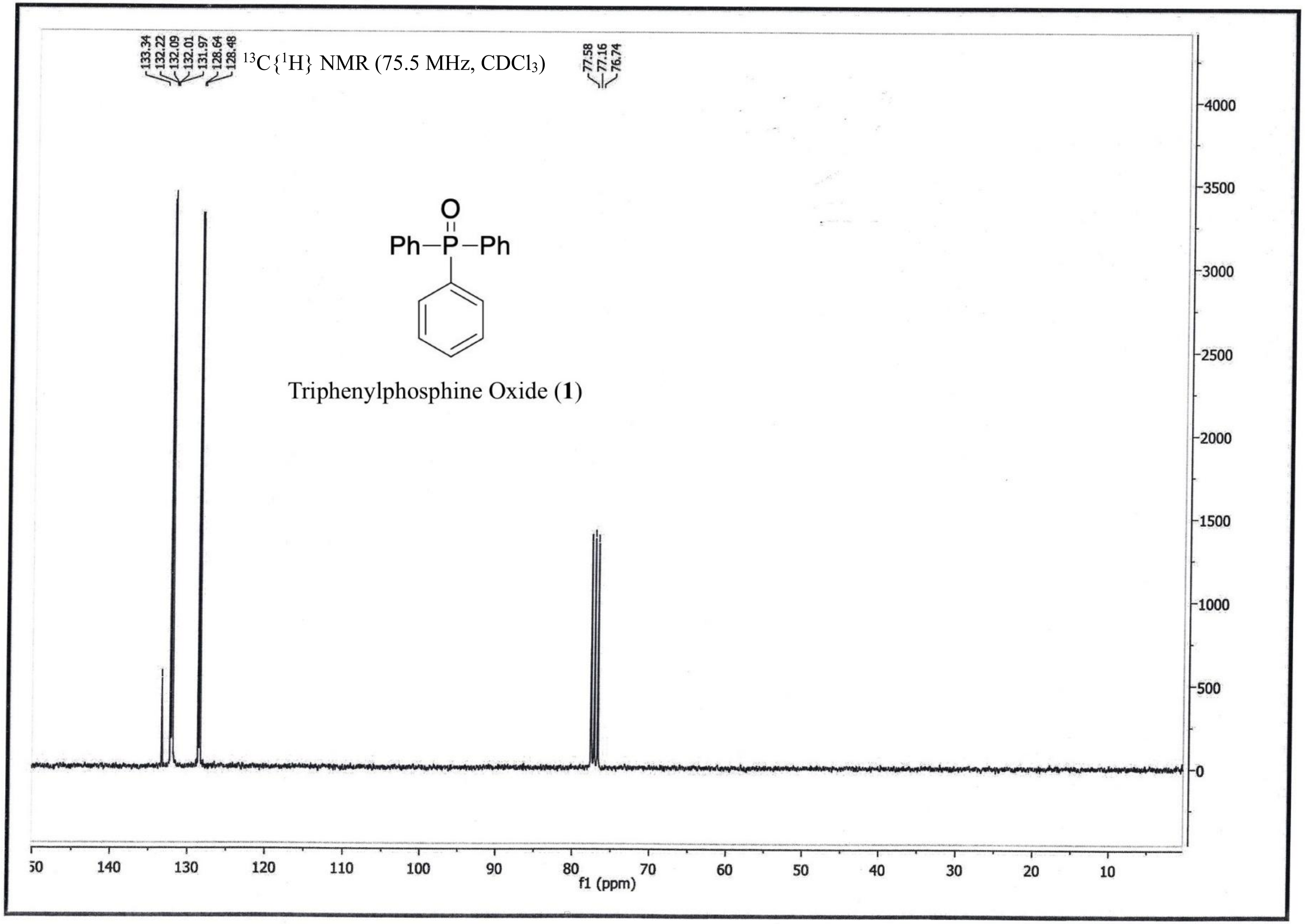

133.34
132.22
132.09
132.01
131.97
128.64
128.48
$^{13}C\{^1H\}$ NMR (75.5 MHz, $CDCl_3$)
77.58
77.16
76.74
O
Ph—P—Ph
Triphenylphosphine Oxide (**1**)
4000
3500
3000
2500
2000
1500
1000
500
0
140
130
120
110
100
90
80
70
60
50
40
30
20
10
f1 (ppm)

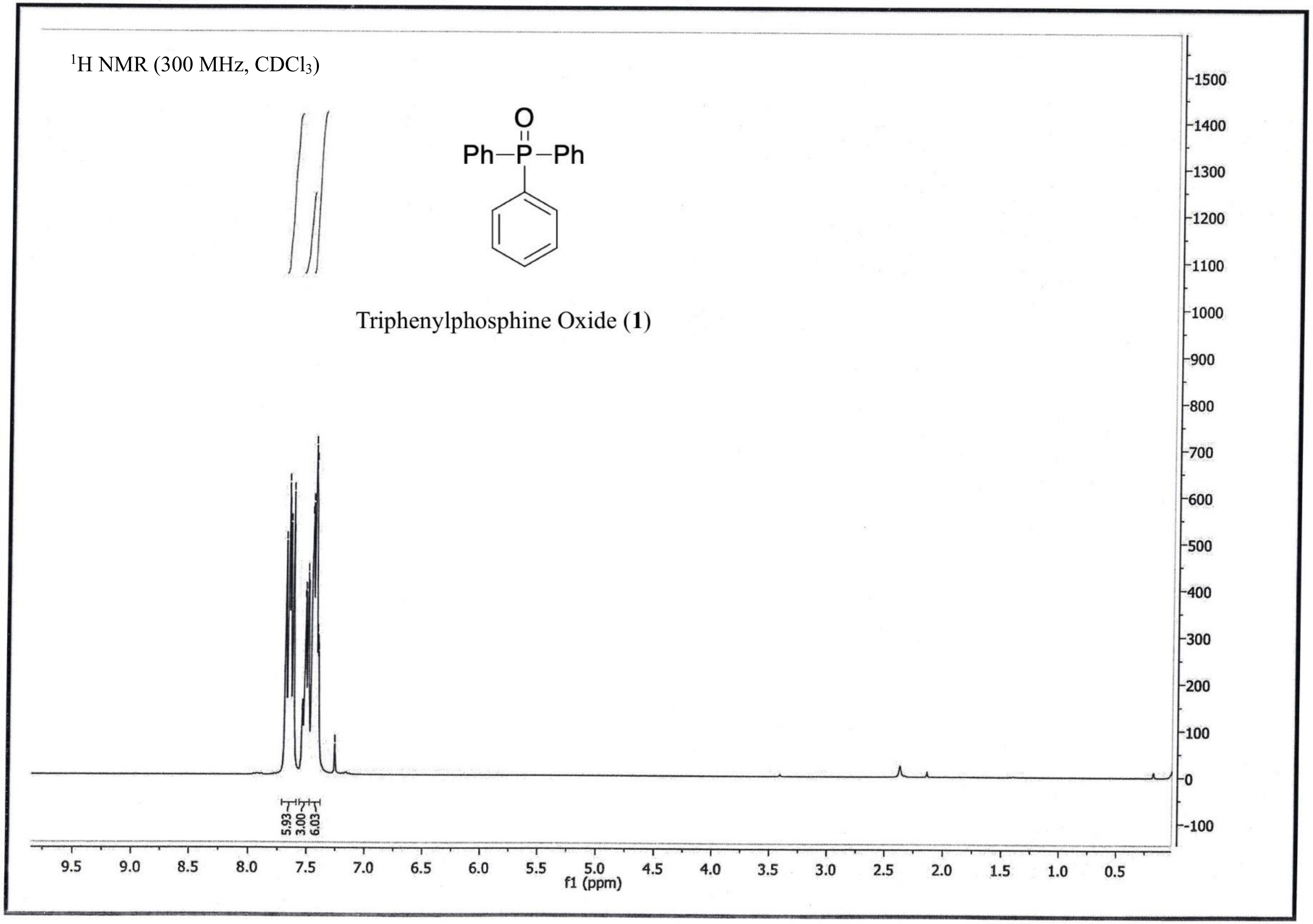

^{1}H NMR (300 MHz, $CDCl_3$)
Ph–P–Ph
O
Triphenylphosphine Oxide (**1**)
5.93
3.00
6.03
9.5 9.0 8.5 8.0 7.5 7.0 6.5 6.0 5.5 5.0 4.5 4.0 3.5 3.0 2.5 2.0 1.5 1.0 0.5
f1 (ppm)
1500 1400 1300 1200 1100 1000 900 800 700 600 500 400 300 200 100 0 -100

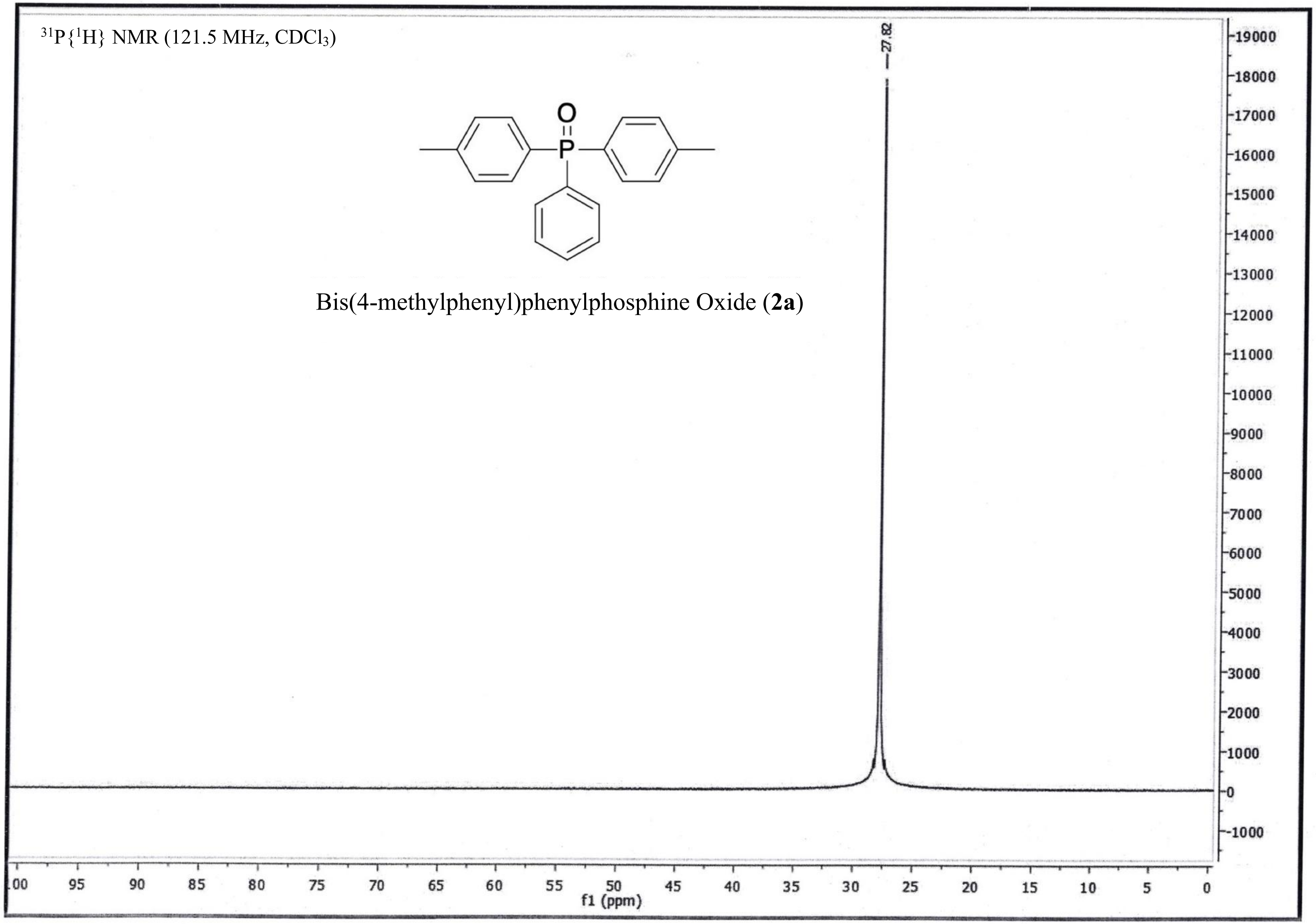
$^{31}P\{^1H\}$ NMR (121.5 MHz, $CDCl_3$)
27.82
Bis(4-methylphenyl)phenylphosphine Oxide (**2a**)
f1 (ppm)

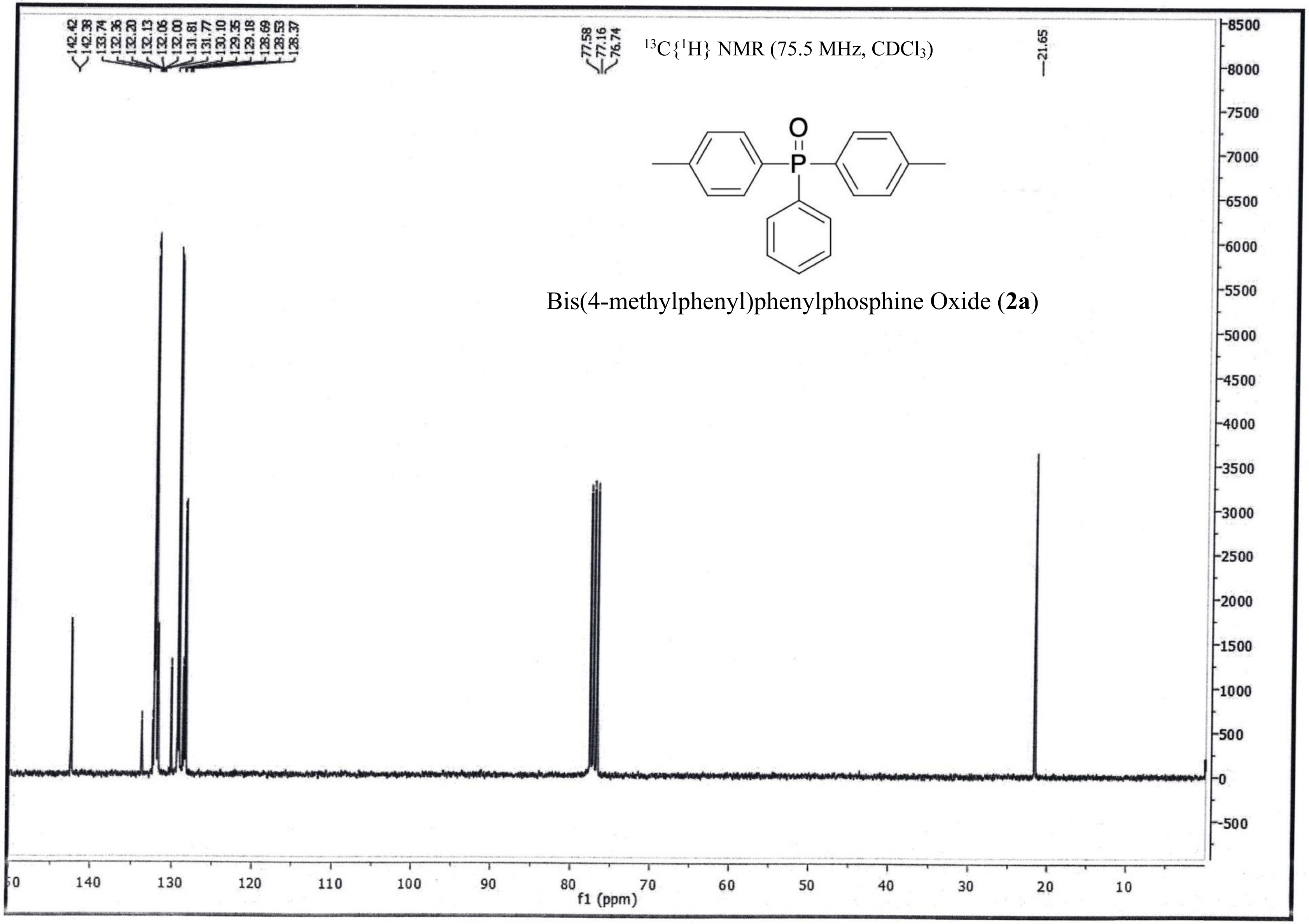

$^{13}C\{^1H\}$ NMR (75.5 MHz, $CDCl_3$)
Bis(4-methylphenyl)phenylphosphine Oxide (**2a**)
f1 (ppm)

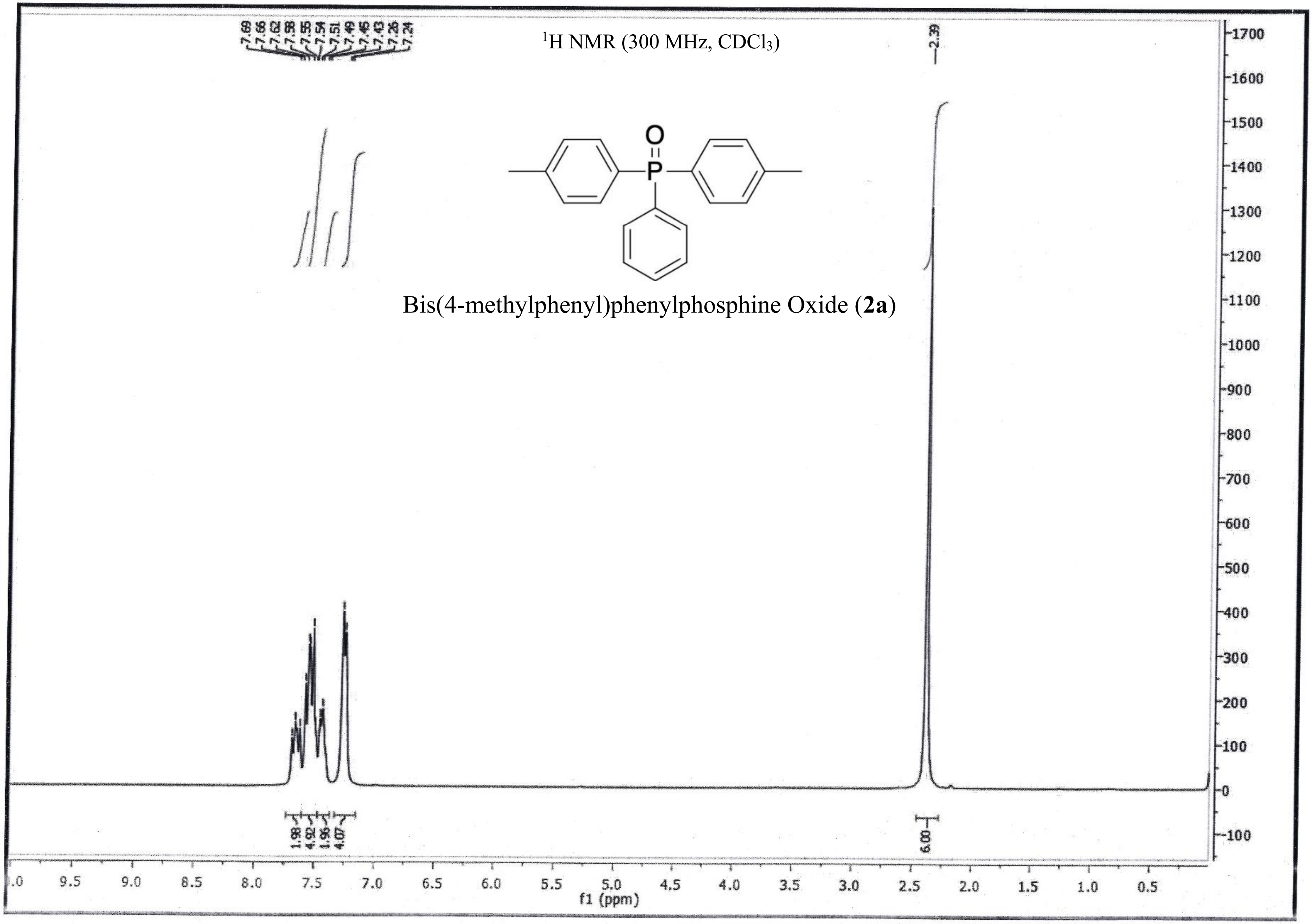

Bis(4-methylphenyl)phenylphosphine Oxide (**2a**)

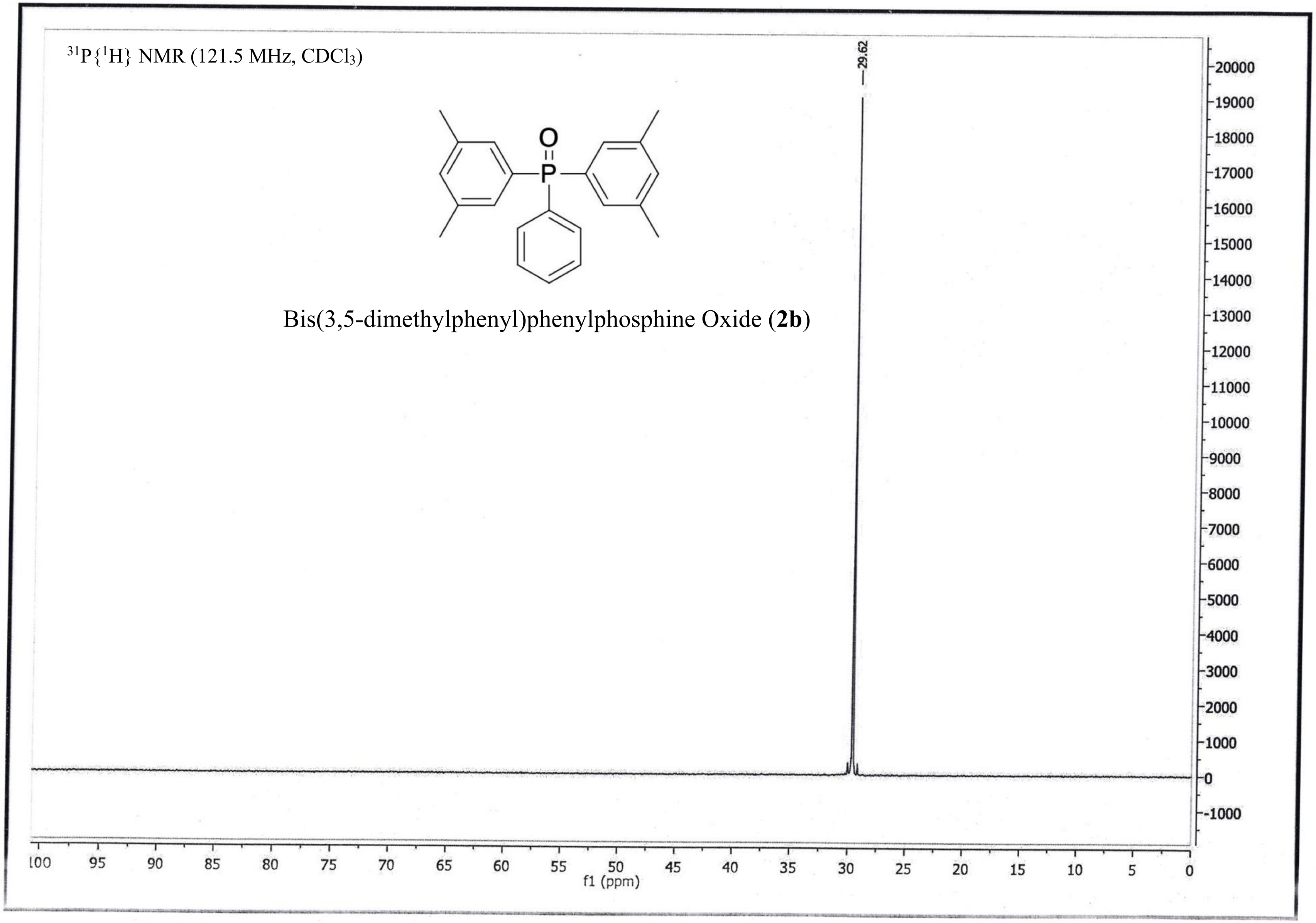

$^{31}P\{^{1}H\}$ NMR (121.5 MHz, $CDCl_3$)
O
P
Bis(3,5-dimethylphenyl)phenylphosphine Oxide (**2b**)
29.62
20000
19000
18000
17000
16000
15000
14000
13000
12000
11000
10000
9000
8000
7000
6000
5000
4000
3000
2000
1000
0
-1000
100 95 90 85 80 75 70 65 60 55 50 45 40 35 30 25 20 15 10 5 0
f1 (ppm)

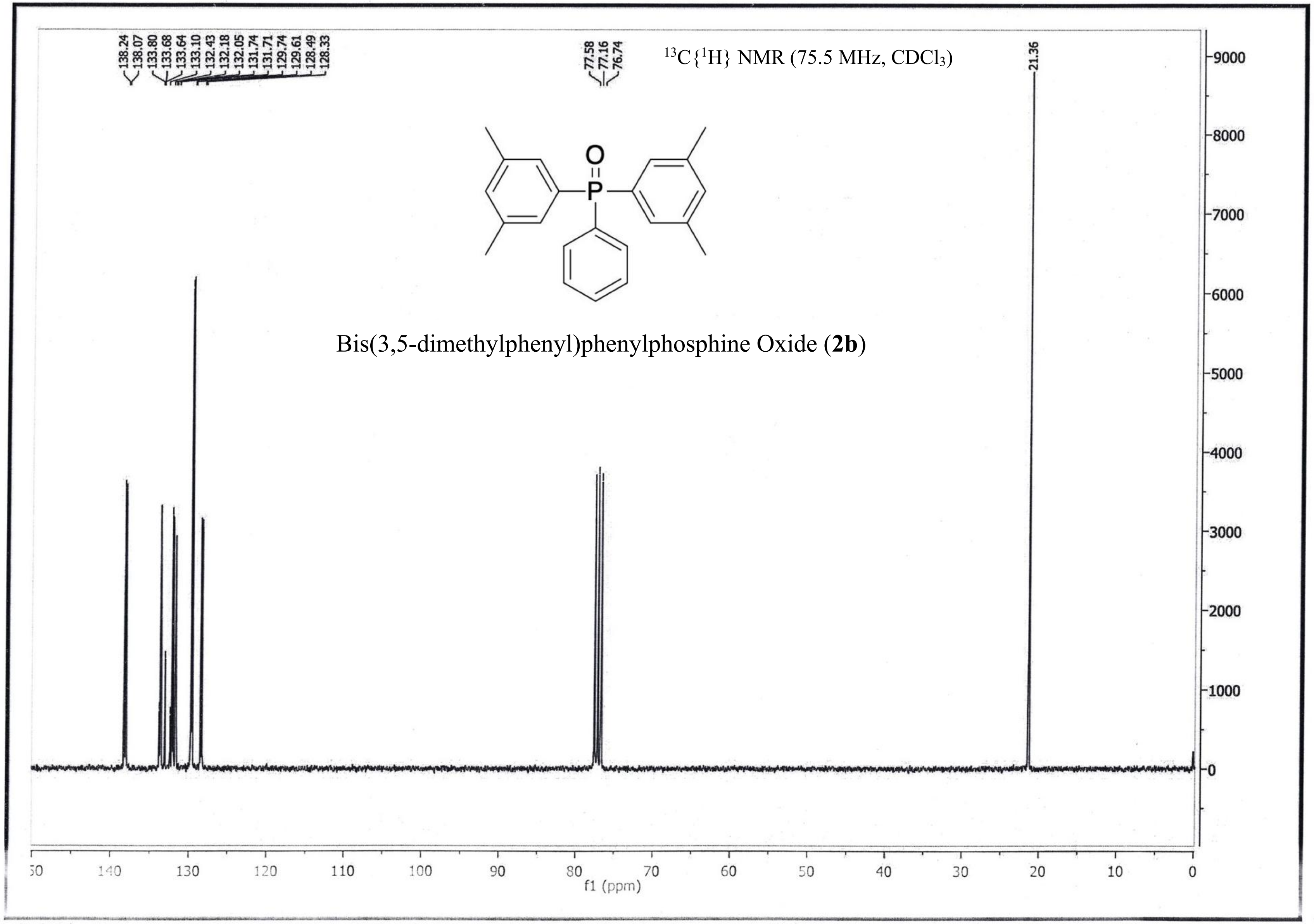
138.24
138.07
133.80
133.68
133.64
133.10
132.43
132.18
132.05
131.74
131.71
129.74
129.61
128.49
128.33
77.58
77.16
76.74
21.36
$^{13}C\{^{1}H\}$ NMR (75.5 MHz, $CDCl_3$)
O
P
Bis(3,5-dimethylphenyl)phenylphosphine Oxide (**2b**)
f1 (ppm)

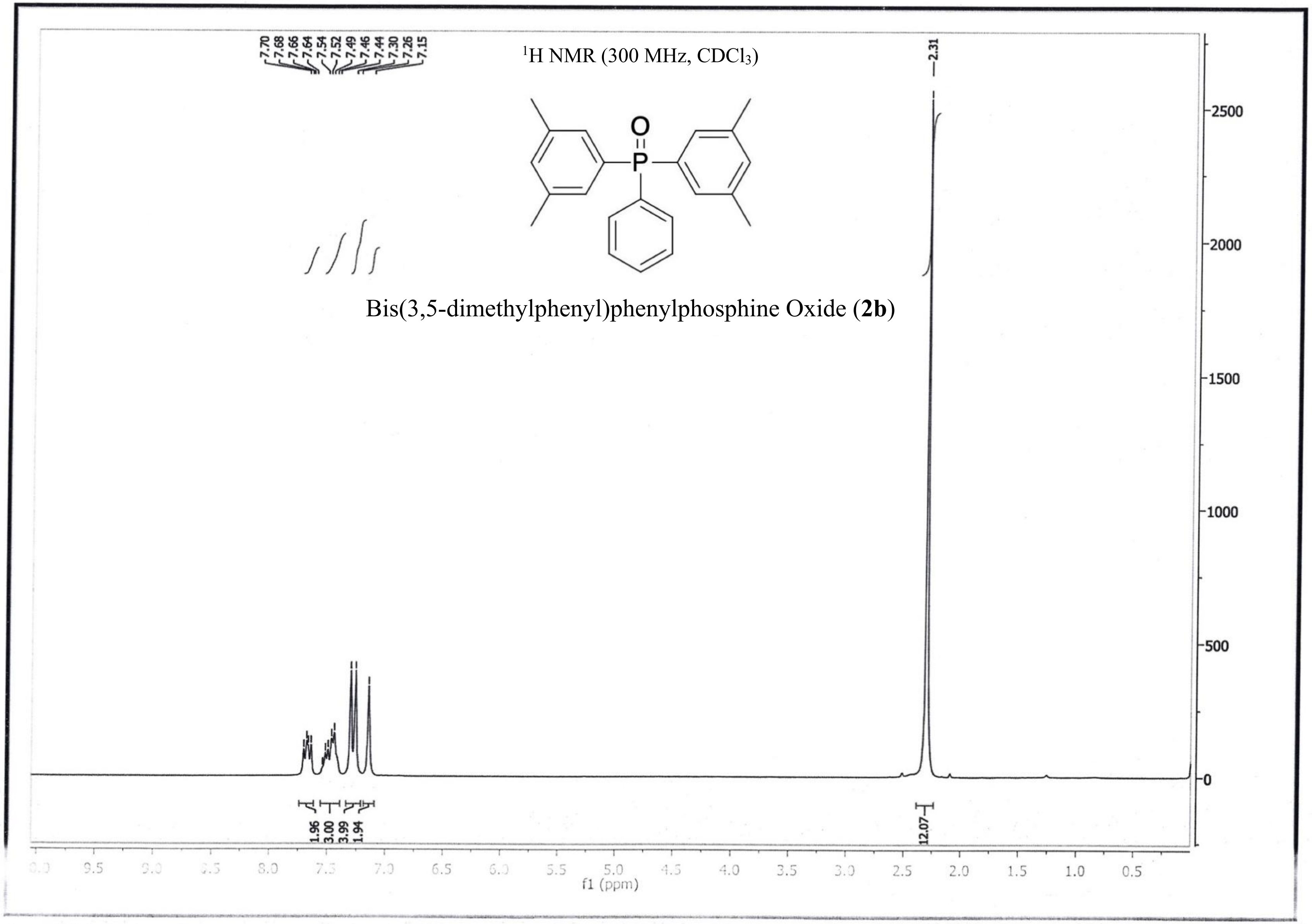

Bis(3,5-dimethylphenyl)phenylphosphine Oxide (**2b**)

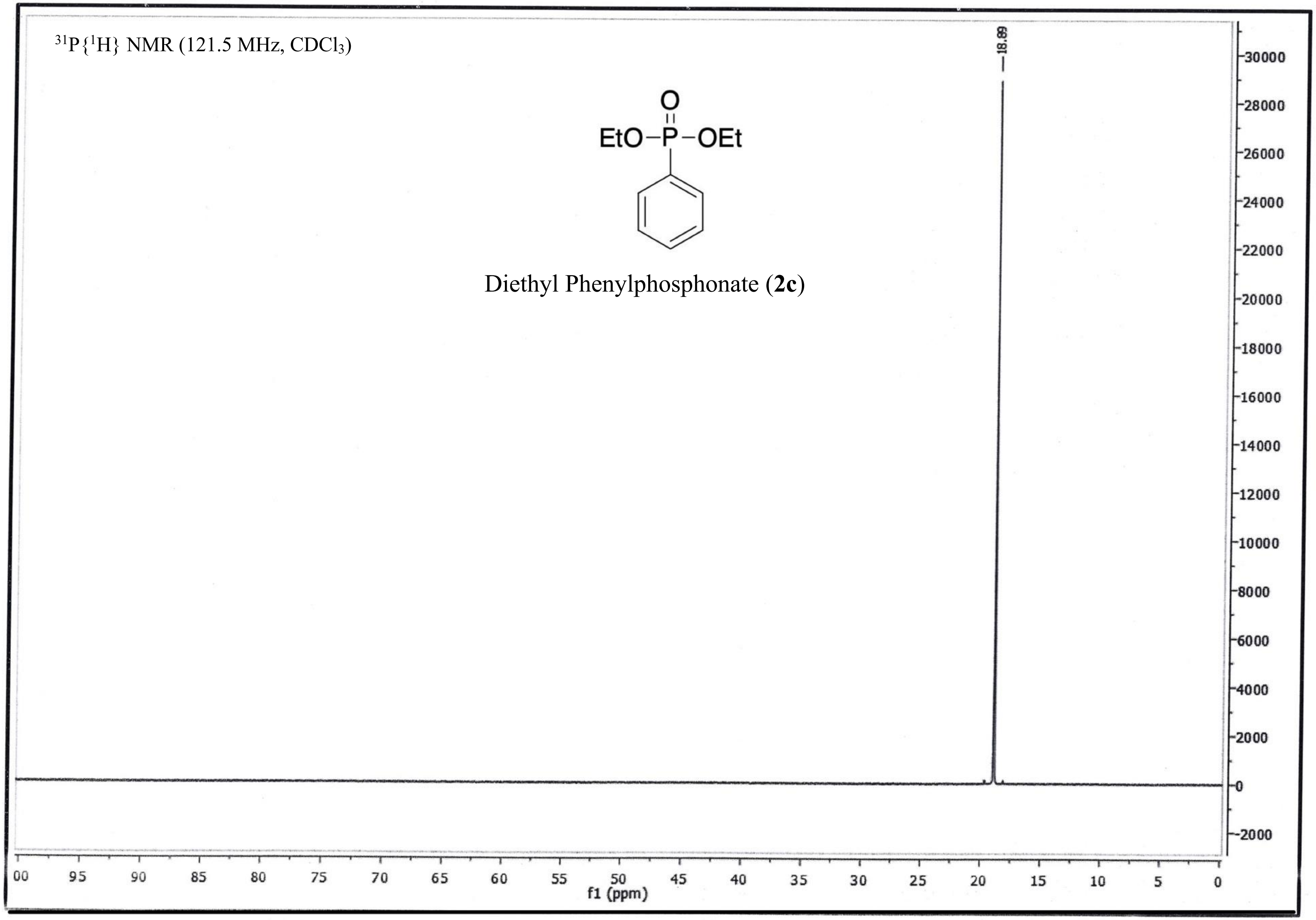

$^{31}P\{^{1}H\}$ NMR (121.5 MHz, $CDCl_3$)
18.89
EtO
P
OEt
O
Diethyl Phenylphosphonate (**2c**)
30000
28000
26000
24000
22000
20000
18000
16000
14000
12000
10000
8000
6000
4000
2000
0
-2000
00
95
90
85
80
75
70
65
60
55
50
45
40
35
30
25
20
15
10
5
0
f1 (ppm)

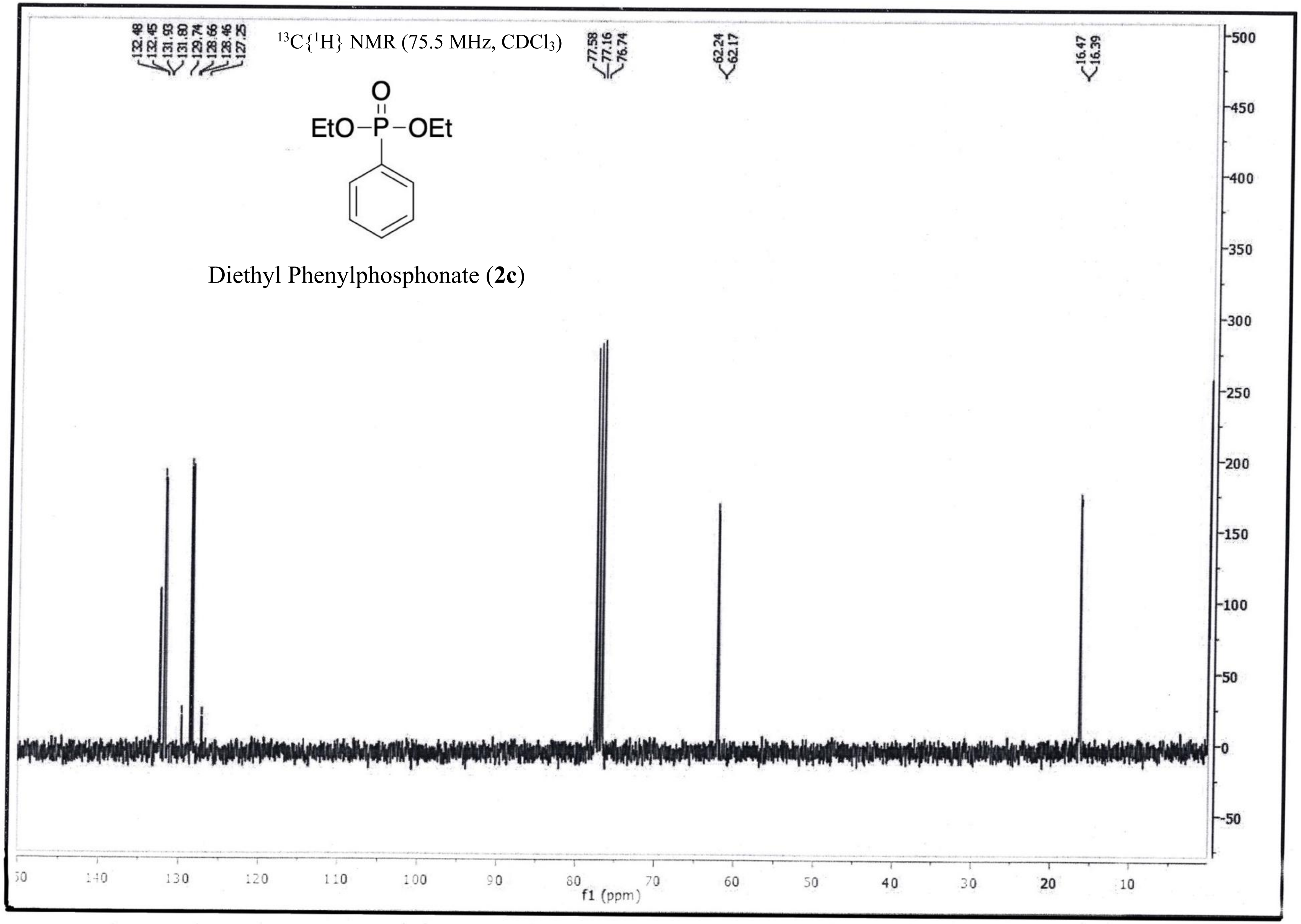

$^{13}C\{^1H\}$ NMR (75.5 MHz, $CDCl_3$)
132.48
132.45
131.93
131.80
129.74
128.66
128.46
127.25
77.58
77.16
76.74
62.24
62.17
16.47
16.39
O
EtO–P–OEt
Diethyl Phenylphosphonate (**2c**)
f1 (ppm)

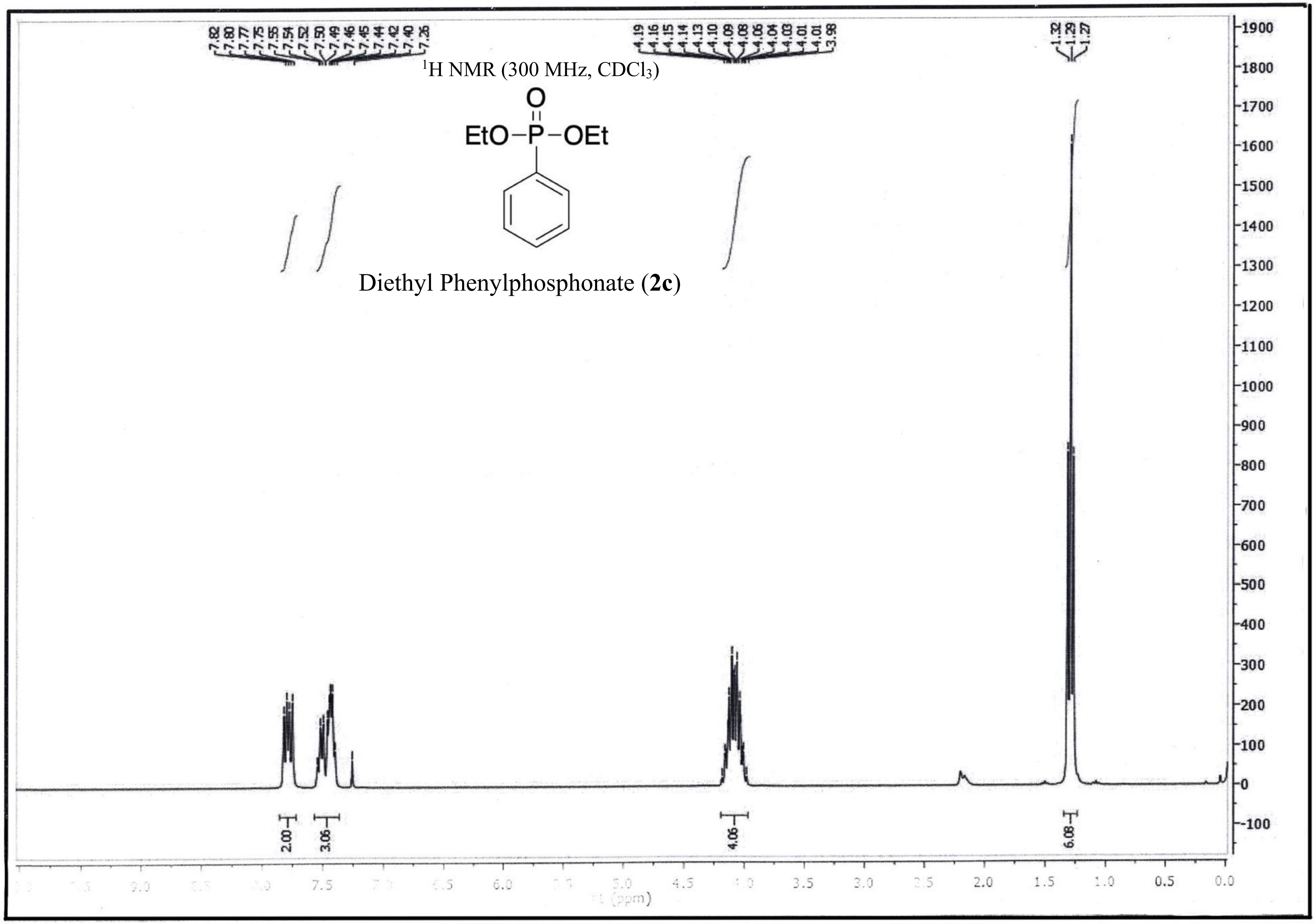

Diethyl Phenylphosphonate (**2c**)

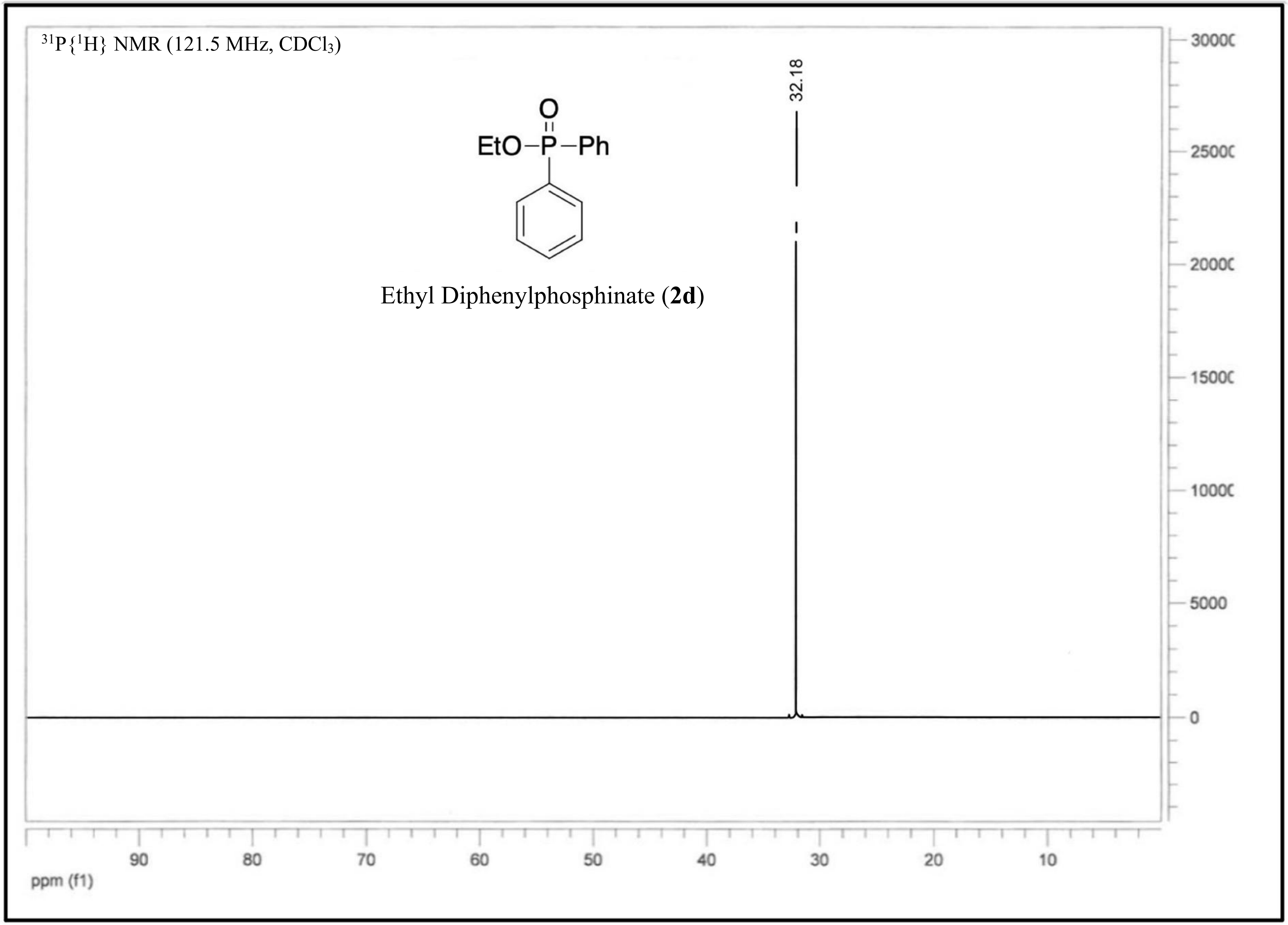

$^{31}P\{^{1}H\}$ NMR (121.5 MHz, $CDCl_3$)
EtO–P–Ph
O
Ethyl Diphenylphosphinate (**2d**)
32.18
30000
25000
20000
15000
10000
5000
0
90
80
70
60
50
40
30
20
10
ppm (f1)

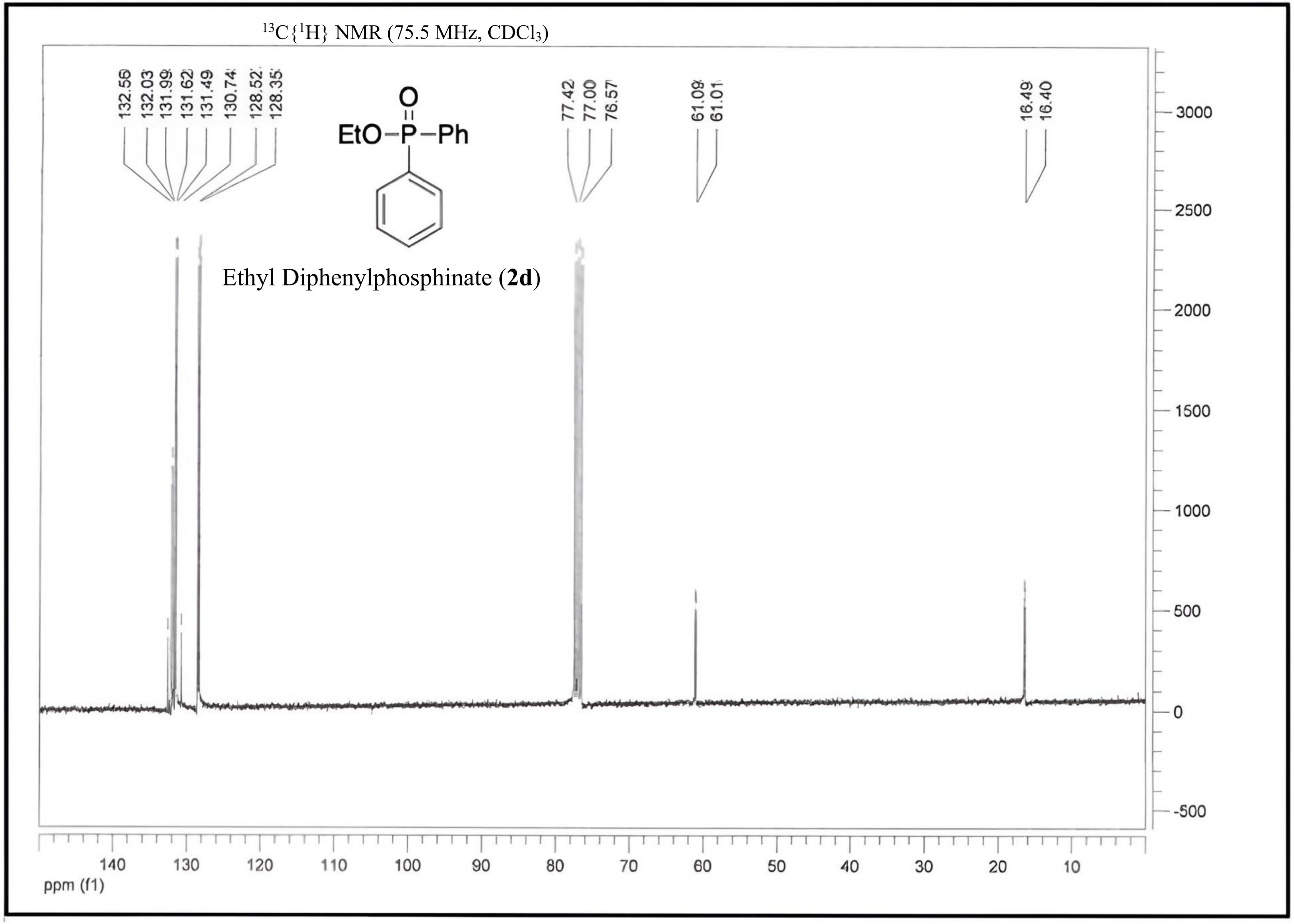

$^{13}C\{^{1}H\}$ NMR (75.5 MHz, $CDCl_3$)
132.56
132.03
131.99
131.62
131.49
130.74
128.52
128.35
77.42
77.00
76.57
61.09
61.01
16.49
16.40
EtO–P–Ph
Ethyl Diphenylphosphinate (**2d**)
ppm (f1)

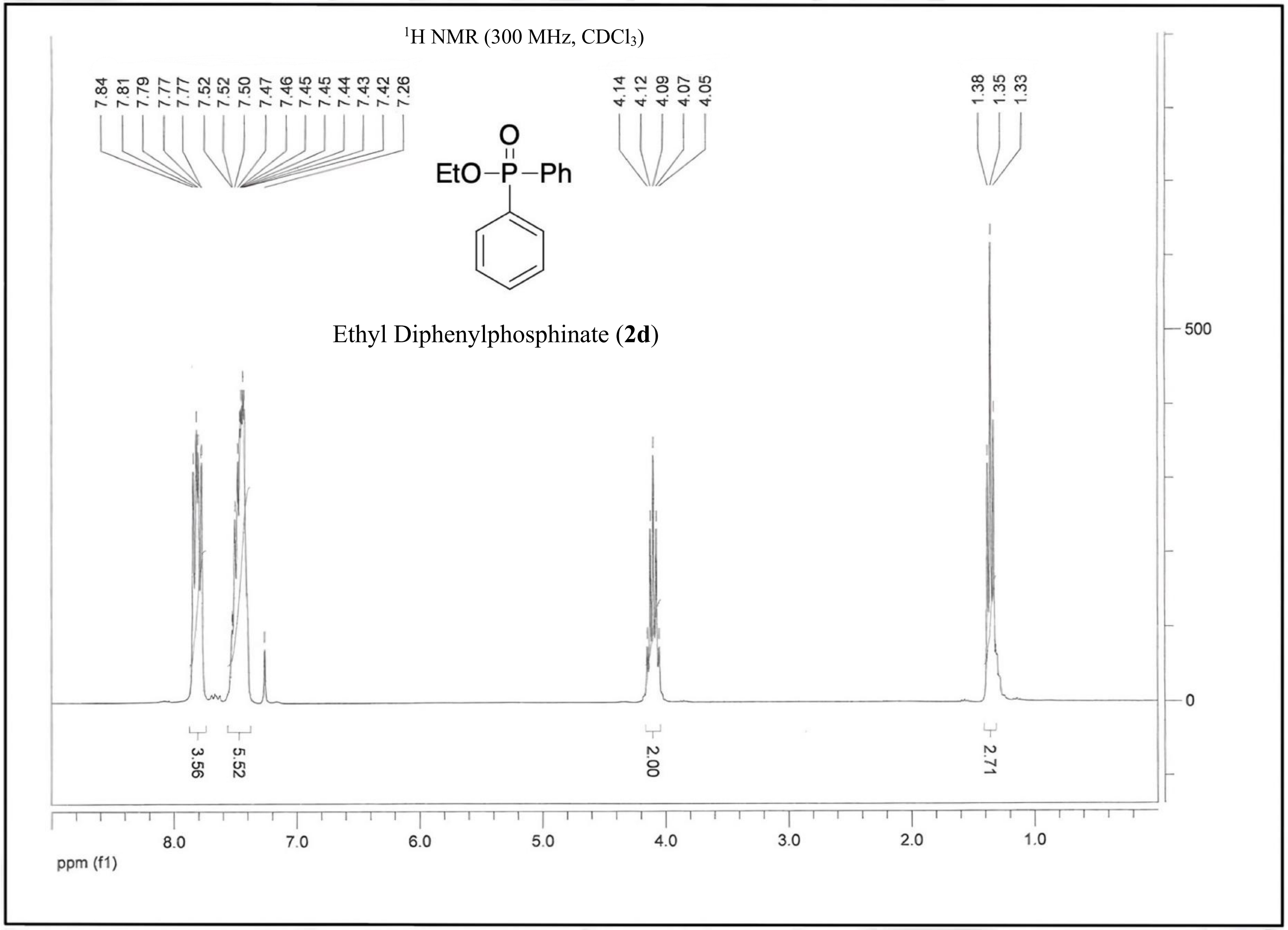

^{1}H NMR (300 MHz, CDCl$_3$)
7.84
7.81
7.79
7.77
7.77
7.52
7.52
7.50
7.47
7.46
7.45
7.45
7.44
7.43
7.42
7.26
4.14
4.12
4.09
4.07
4.05
1.38
1.35
1.33
EtO
P
Ph
O
Ethyl Diphenylphosphinate (**2d**)
500
0
3.56
5.52
2.00
2.71
8.0
7.0
6.0
5.0
4.0
3.0
2.0
1.0
ppm (f1)

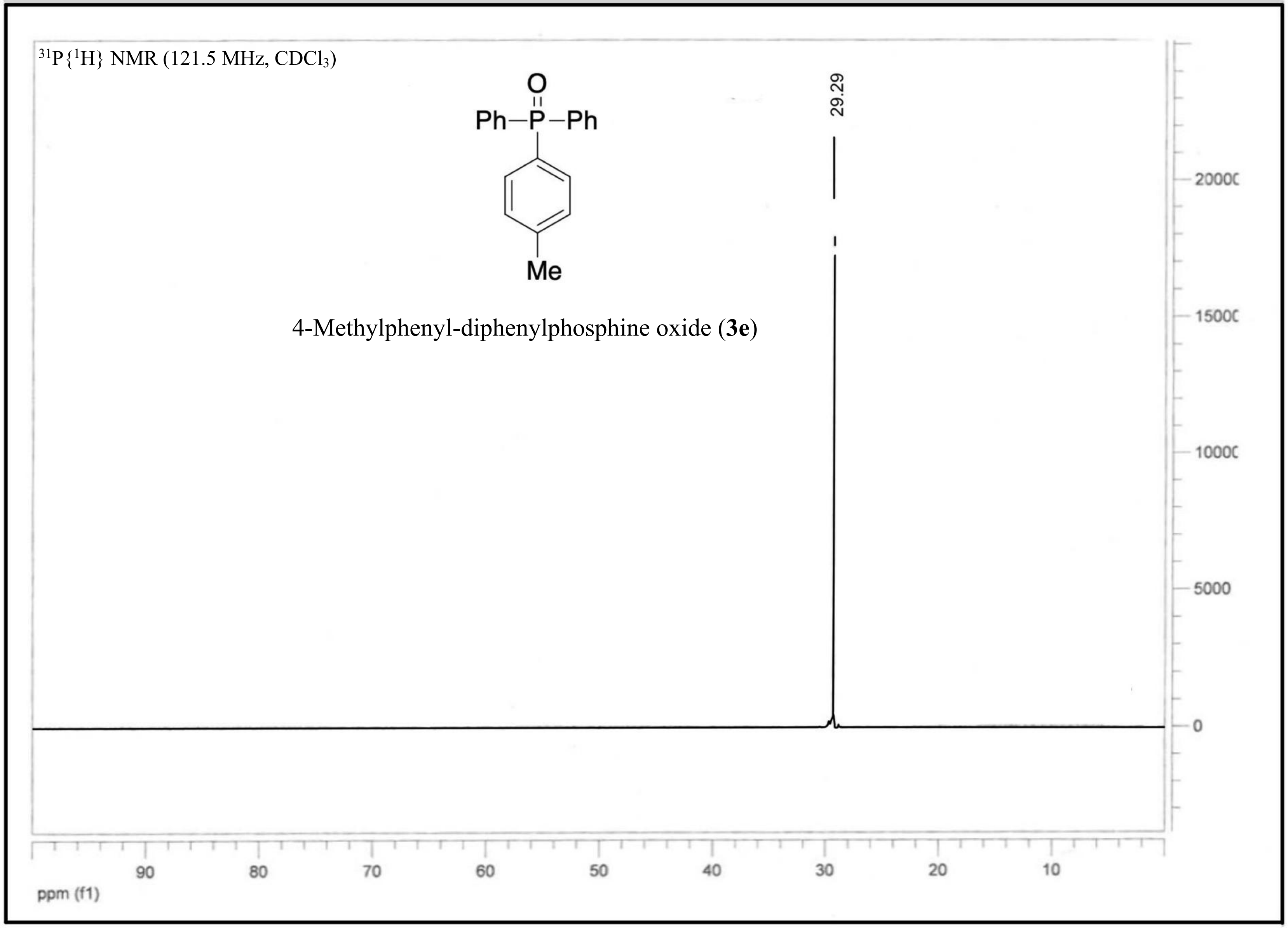

$^{31}P\{^{1}H\}$ NMR (121.5 MHz, $CDCl_3$)
Ph–P–Ph
O
Me
4-Methylphenyl-diphenylphosphine oxide (**3e**)
29.29
20000
15000
10000
5000
0
90
80
70
60
50
40
30
20
10
ppm (f1)

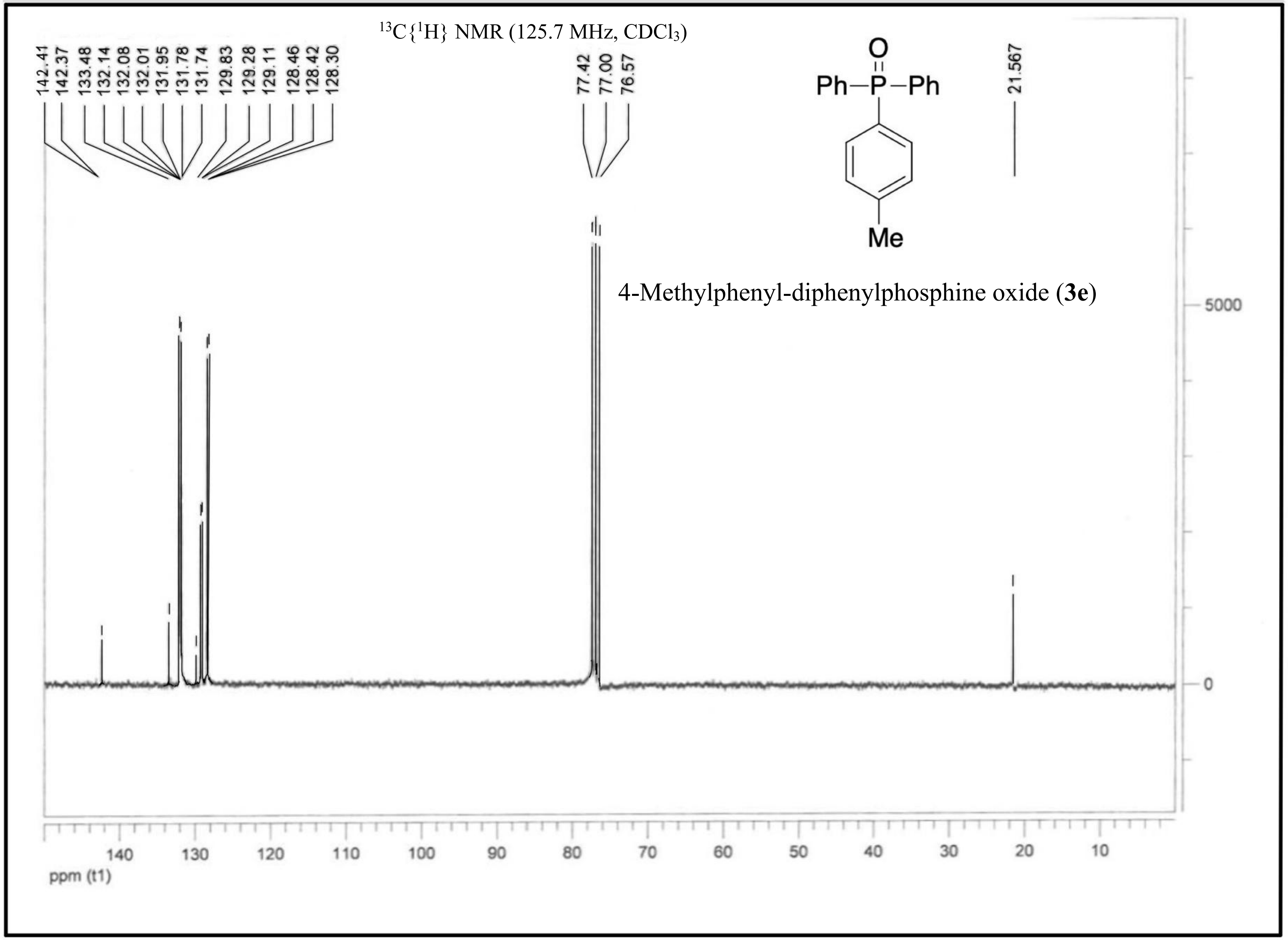

13C{1H} NMR (125.7 MHz, CDCl3)
142.41
142.37
133.48
132.14
132.08
132.01
131.95
131.78
131.74
129.83
129.28
129.11
128.46
128.42
128.30
77.42
77.00
76.57
21.567
O
Ph
P
Ph
Me
4-Methylphenyl-diphenylphosphine oxide (3e)
5000
0
140
130
120
110
100
90
80
70
60
50
40
30
20
10
ppm (t1)

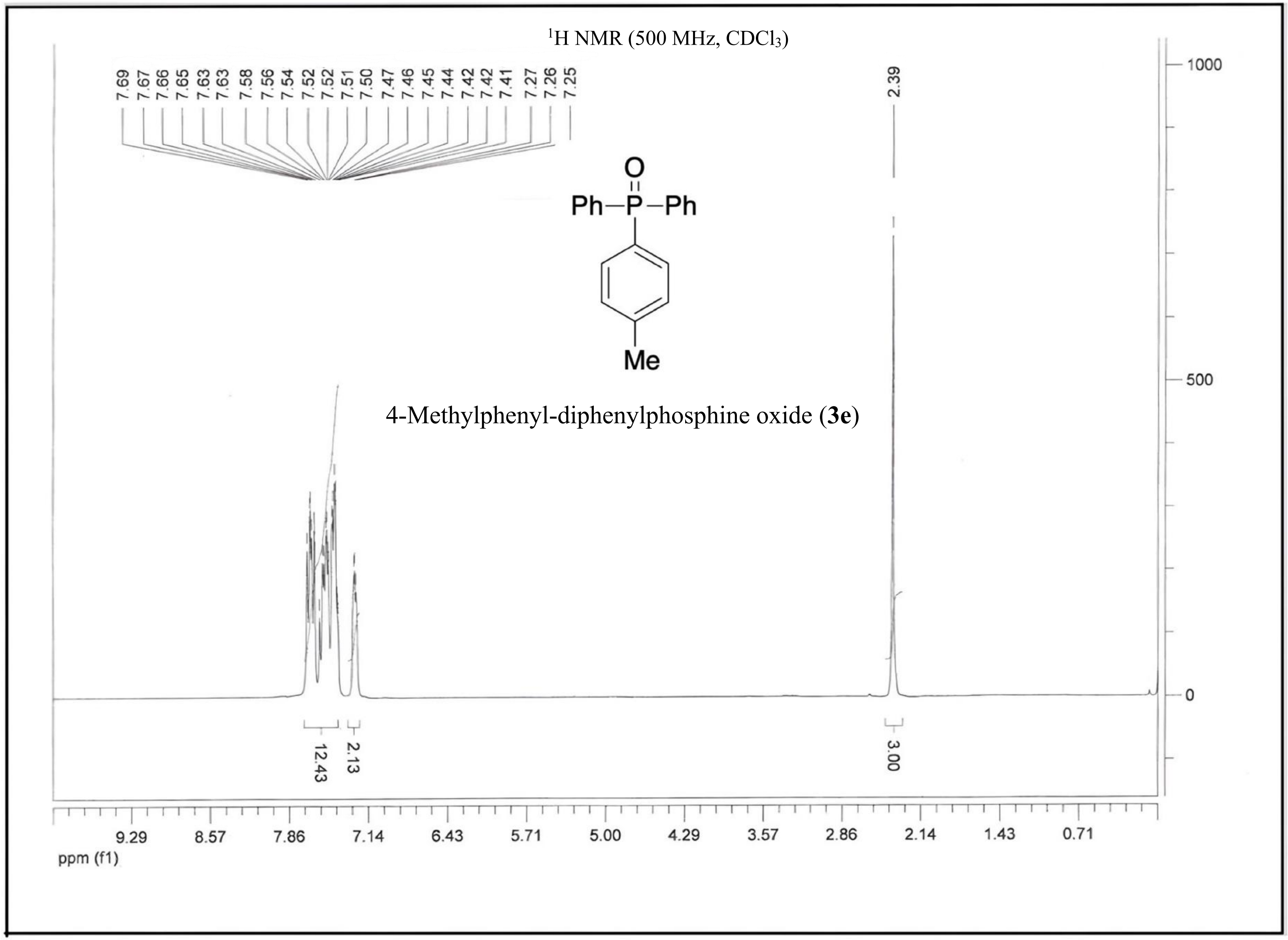

^{1}H NMR (500 MHz, CDCl$_3$)
7.69 7.67 7.66 7.65 7.63 7.63 7.58 7.56 7.54 7.52 7.52 7.51 7.50 7.47 7.46 7.45 7.44 7.42 7.42 7.41 7.27 7.26 7.25
2.39
O
Ph–P–Ph
Me
4-Methylphenyl-diphenylphosphine oxide (**3e**)
12.43
2.13
3.00
1000
500
0
9.29 8.57 7.86 7.14 6.43 5.71 5.00 4.29 3.57 2.86 2.14 1.43 0.71
ppm (f1)

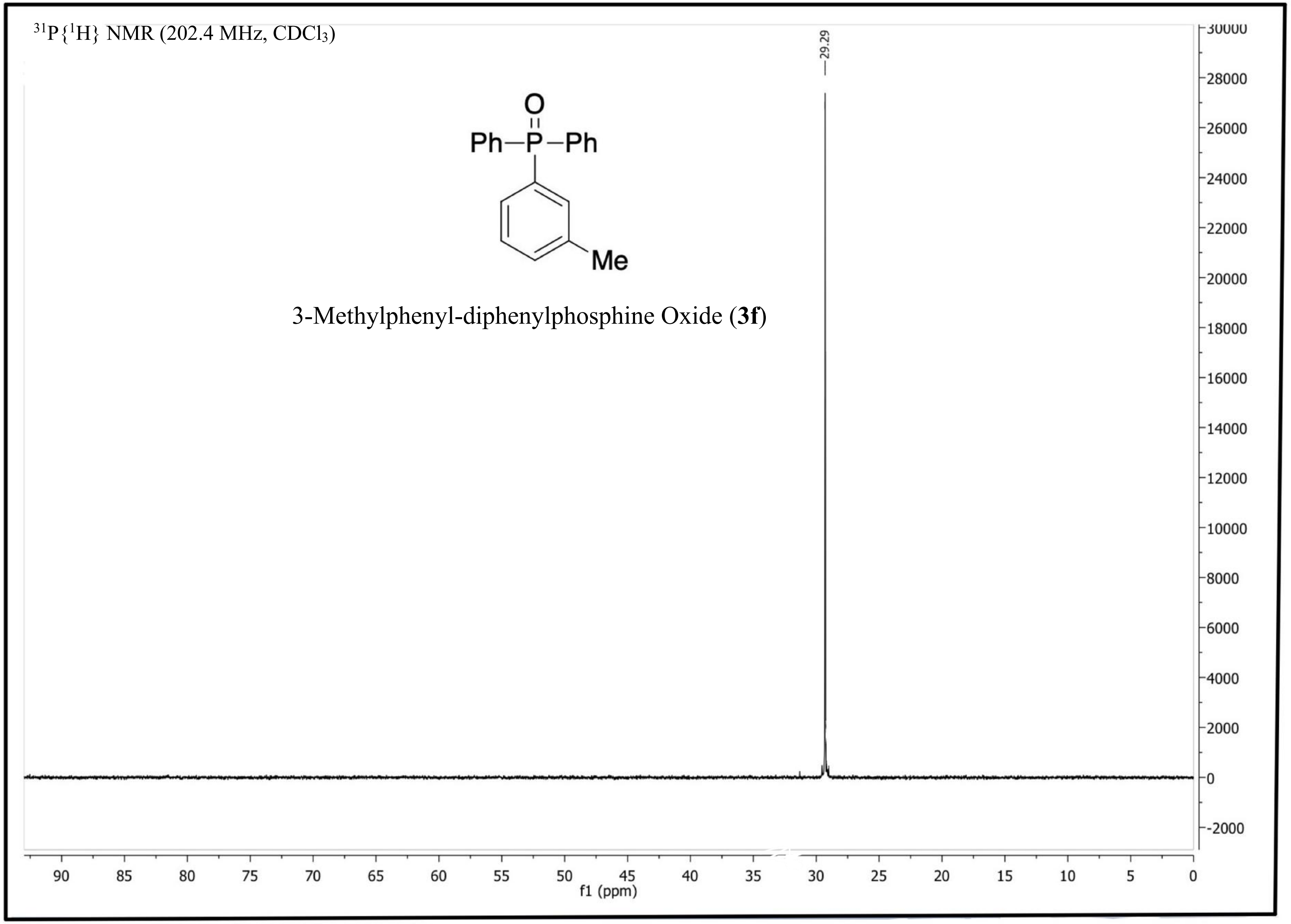
$^{31}P\{^{1}H\}$ NMR (202.4 MHz, $CDCl_3$)
29.29
Ph–P(=O)–Ph
Me
3-Methylphenyl-diphenylphosphine Oxide (**3f**)
f1 (ppm)

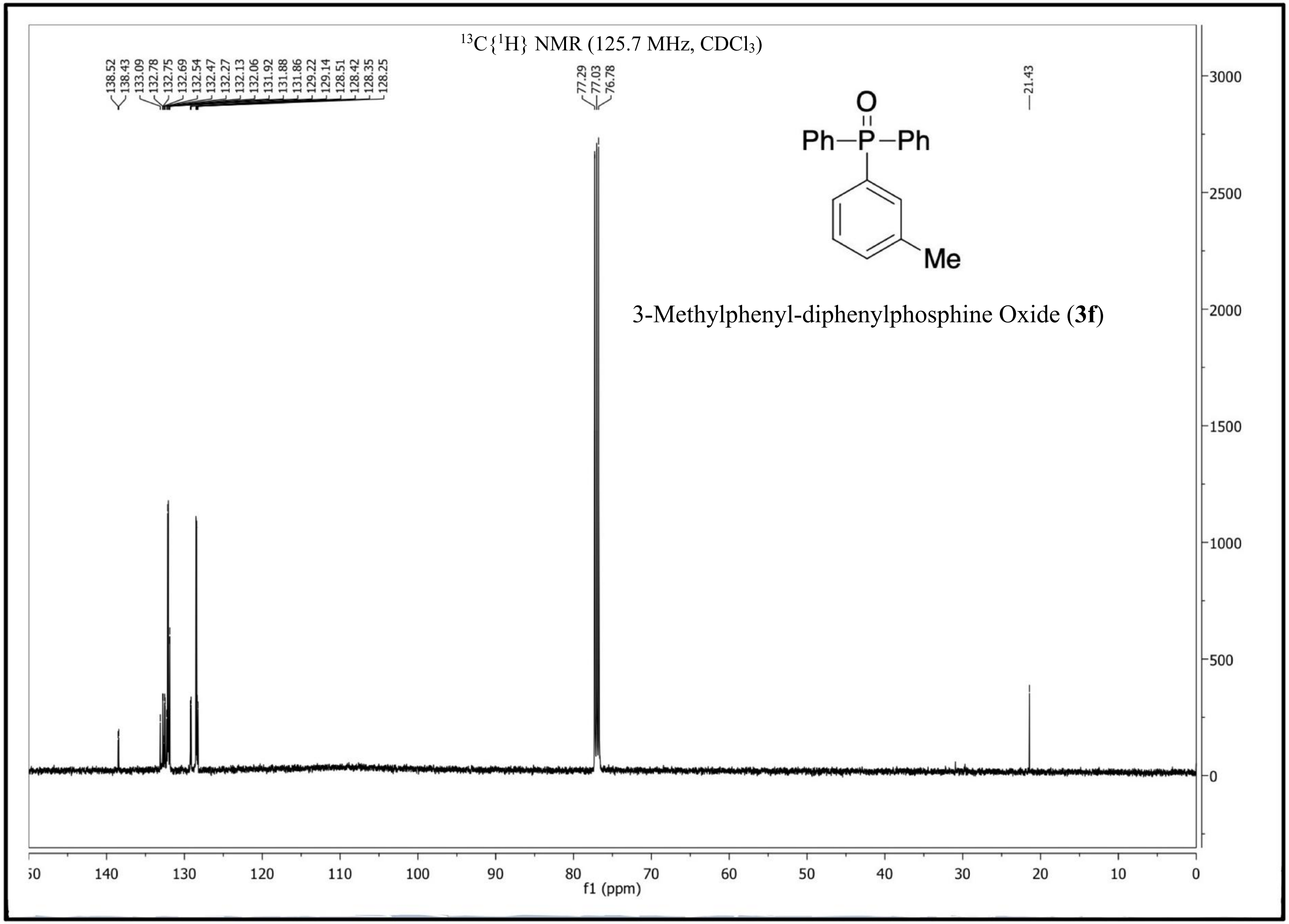
13C{1H} NMR (125.7 MHz, CDCl3)
138.52
138.43
133.09
132.78
132.75
132.69
132.54
132.47
132.27
132.13
132.06
131.92
131.88
131.86
129.22
129.14
128.51
128.42
128.35
128.25
77.29
77.03
76.78
21.43
O
Ph
P
Ph
Me
3-Methylphenyl-diphenylphosphine Oxide (3f)
f1 (ppm)

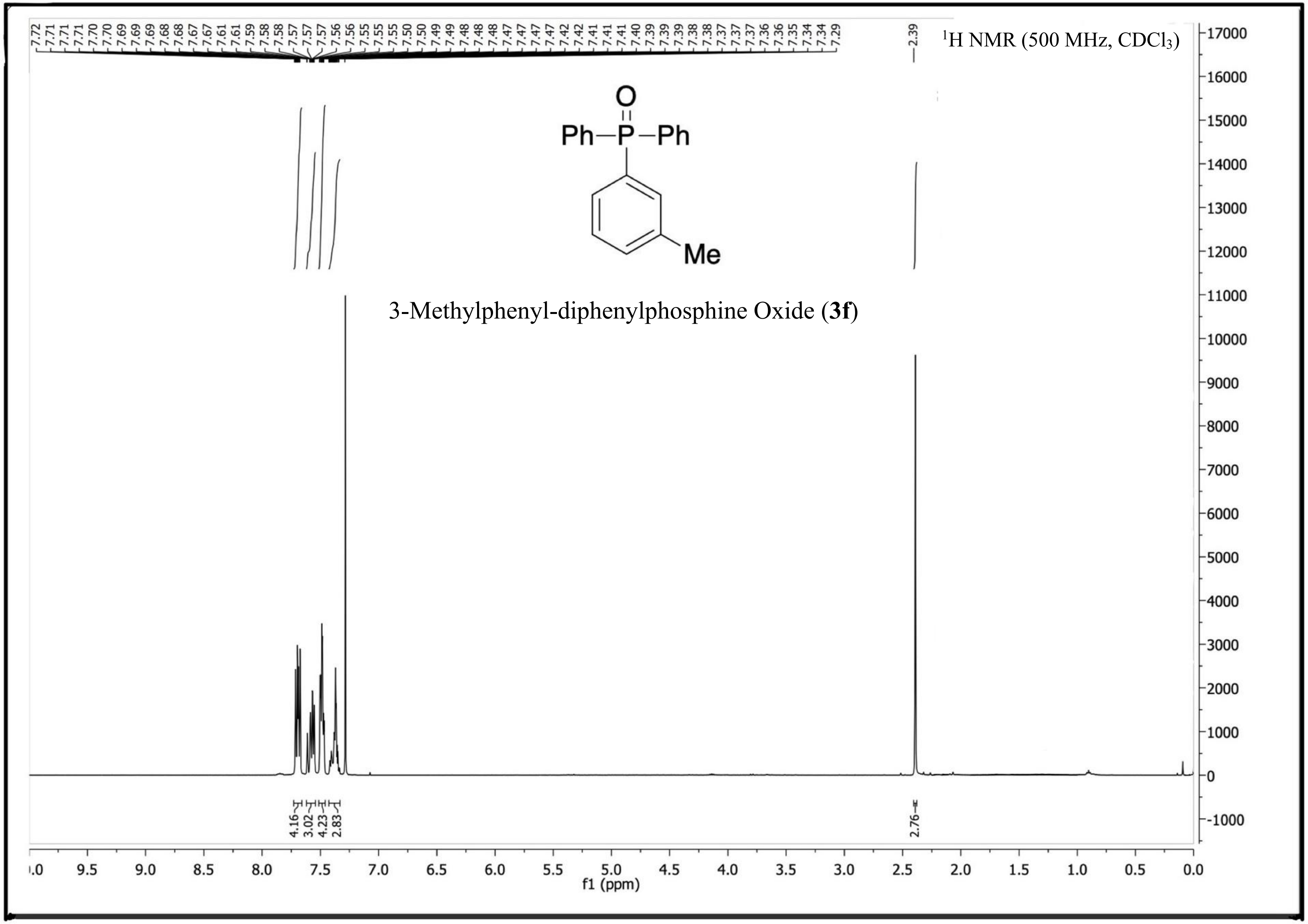

^{1}H NMR (500 MHz, $CDCl_3$)
3-Methylphenyl-diphenylphosphine Oxide (**3f**)
f1 (ppm)

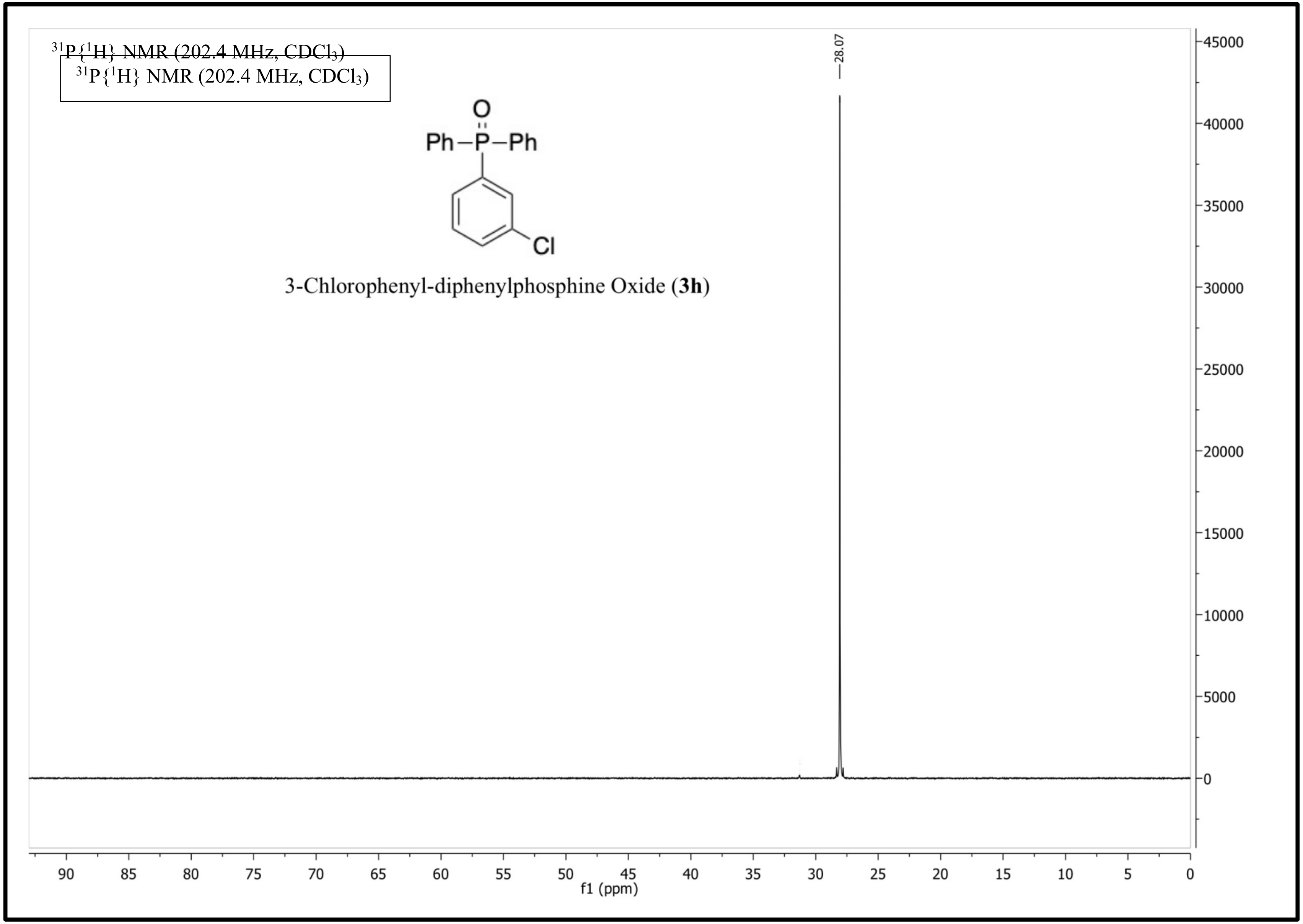

3-Chlorophenyl-diphenylphosphine Oxide (**3h**)

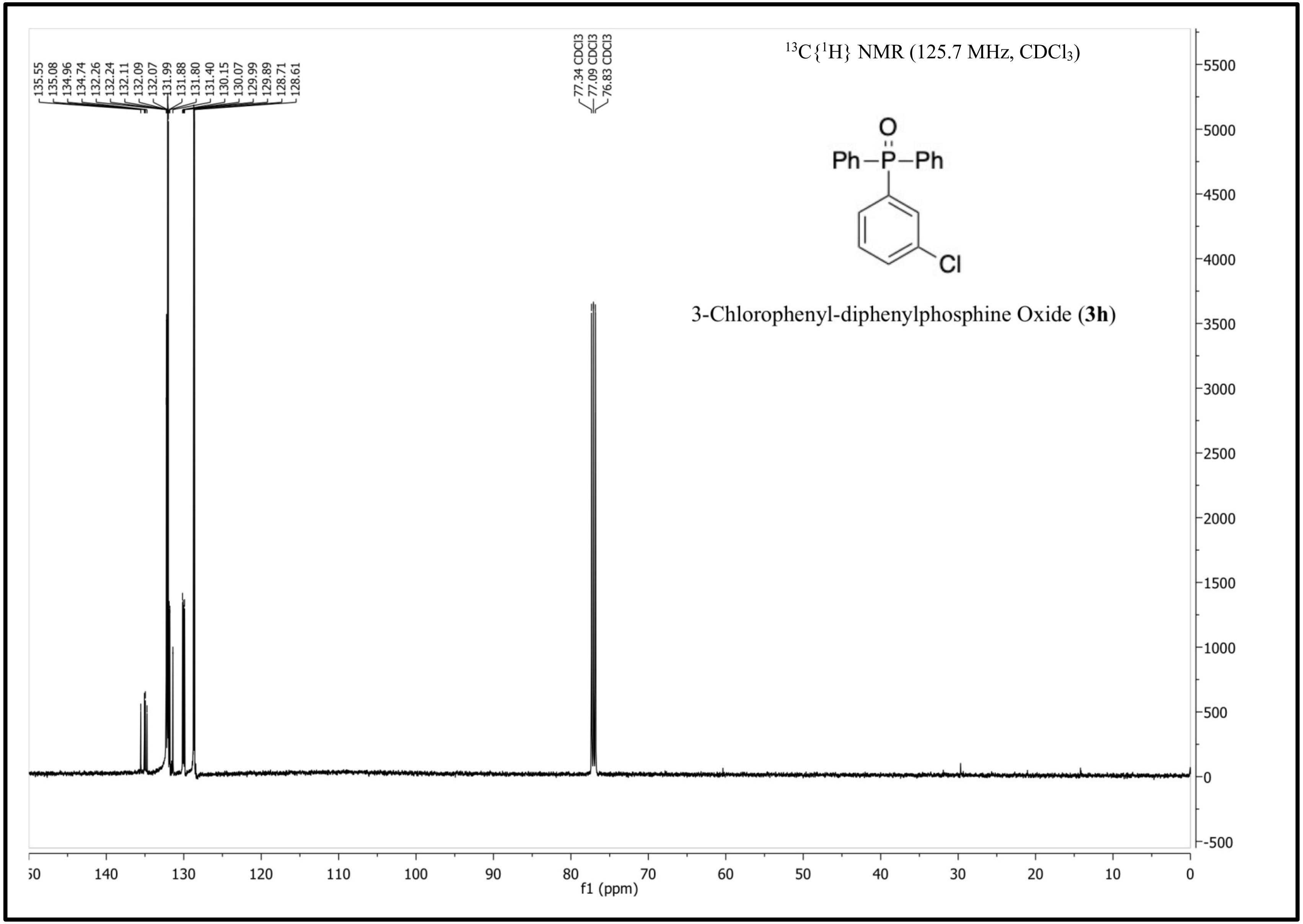

135.55
135.08
134.96
134.74
132.26
132.24
132.11
132.09
132.07
131.99
131.88
131.80
131.40
130.15
130.07
129.99
129.89
128.71
128.61
77.34 CDCl3
77.09 CDCl3
76.83 CDCl3
$^{13}C\{^1H\}$ NMR (125.7 MHz, $CDCl_3$)
O
Ph–P–Ph
Cl
3-Chlorophenyl-diphenylphosphine Oxide (**3h**)
f1 (ppm)

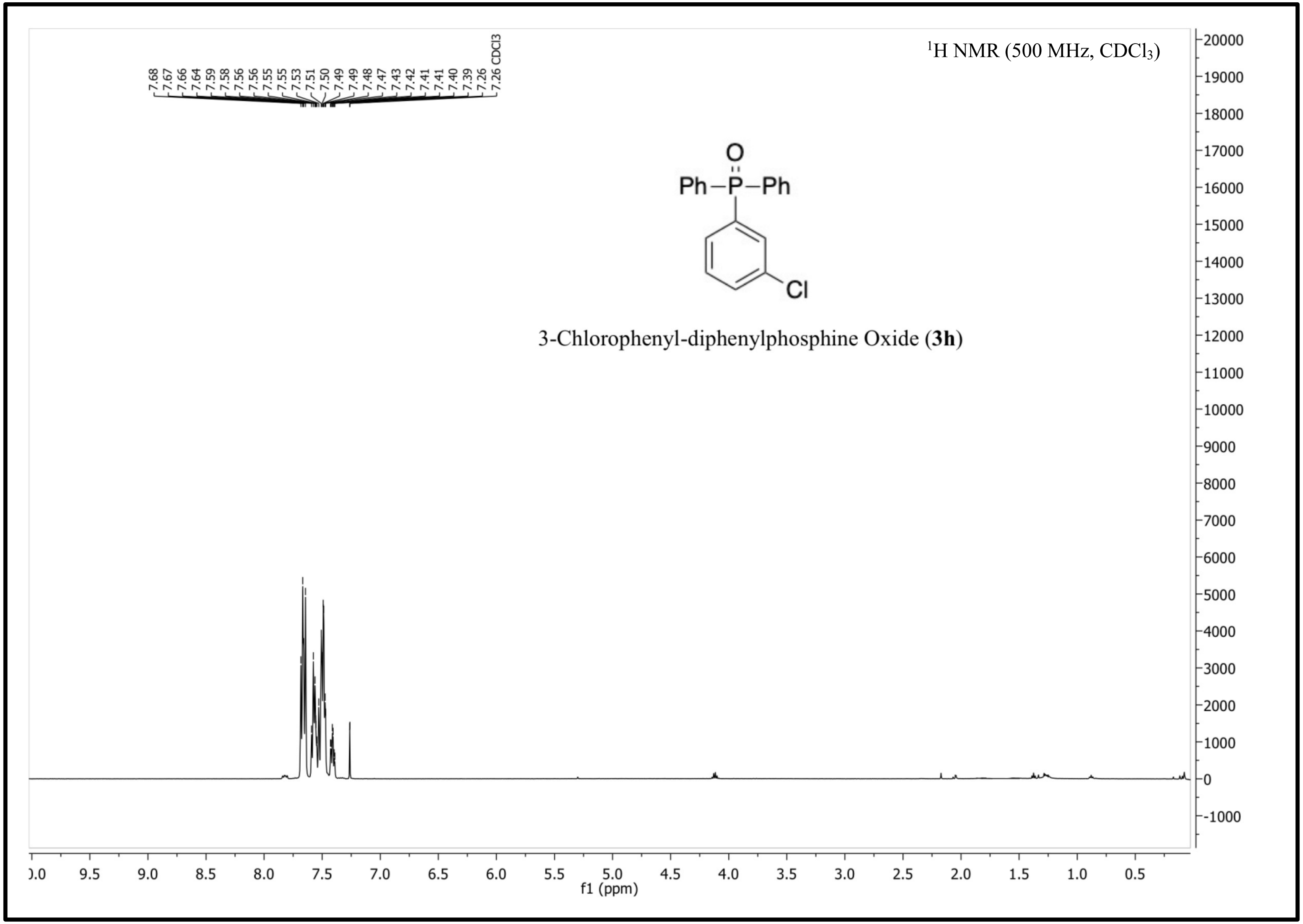

¹H NMR (500 MHz, CDCl₃)
3-Chlorophenyl-diphenylphosphine Oxide (3h)
f1 (ppm)

$^{31}P\{^{1}H\}$ NMR (202.4 MHz, $CDCl_3$)

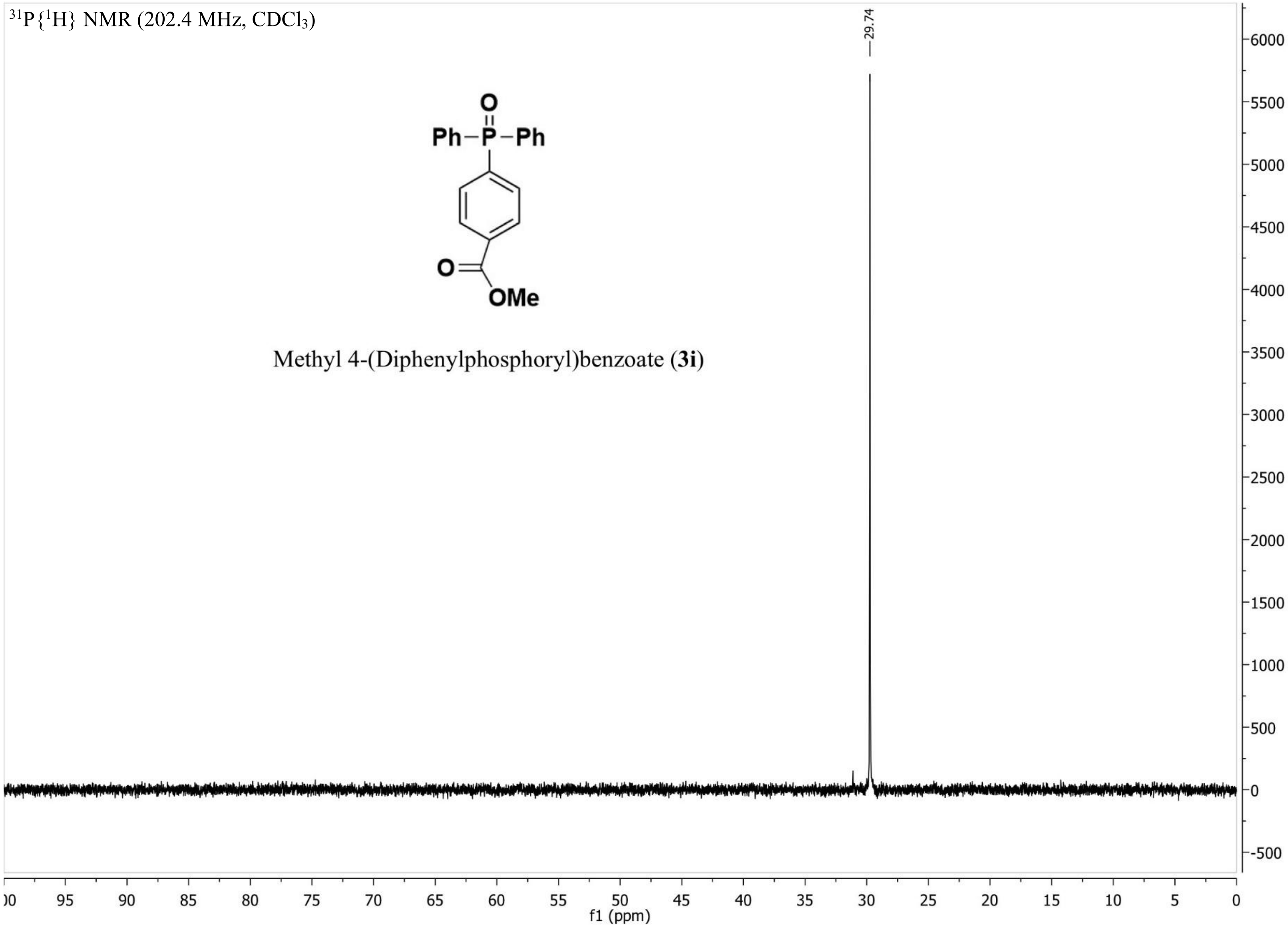

Methyl 4-(Diphenylphosphoryl)benzoate (**3i**)

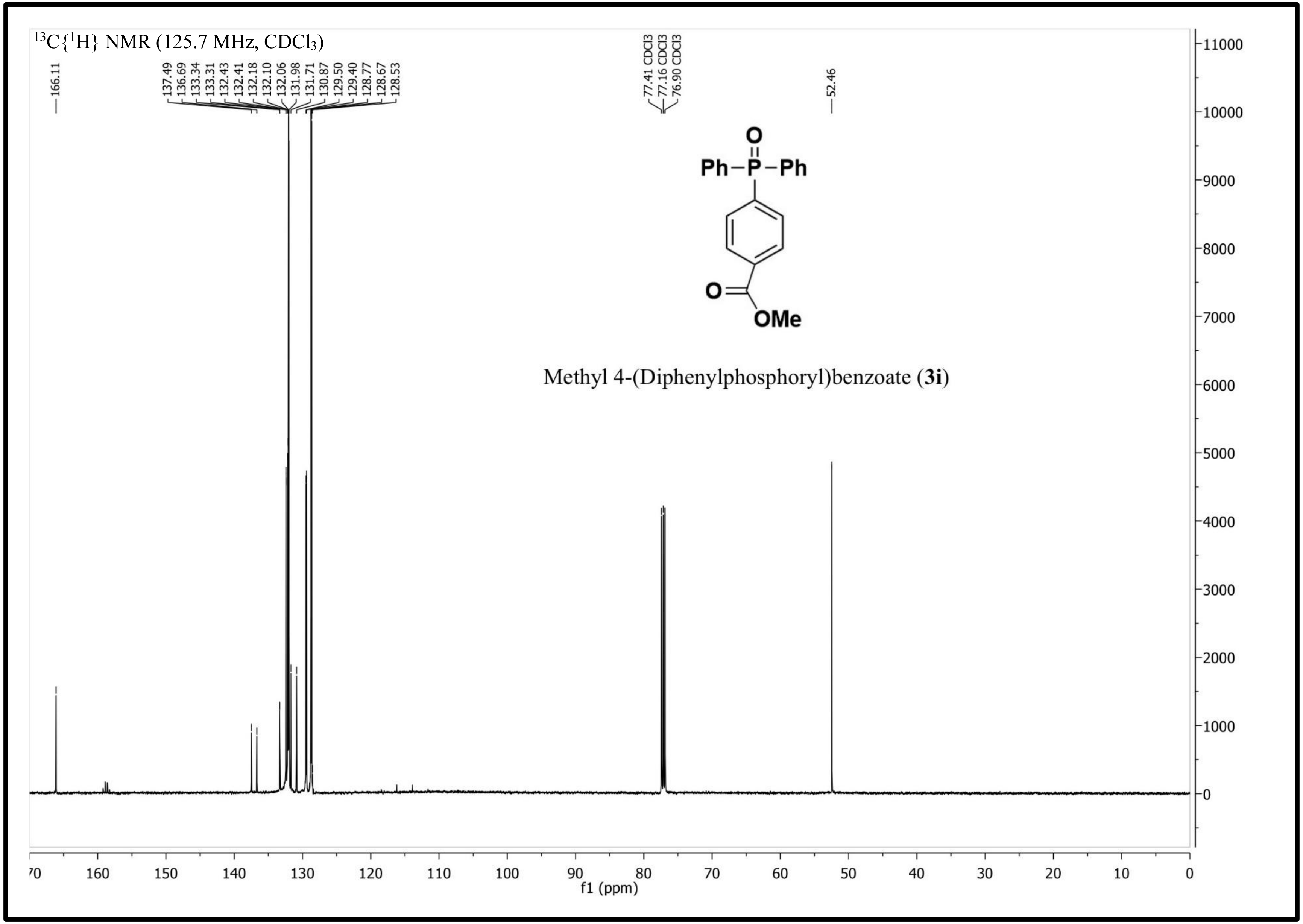

$^{13}C\{^{1}H\}$ NMR (125.7 MHz, $CDCl_3$)
166.11
137.49
136.69
133.34
133.31
132.43
132.41
132.18
132.10
132.06
131.98
131.71
130.87
129.50
129.40
128.77
128.67
128.53
77.41 CDCl3
77.16 CDCl3
76.90 CDCl3
52.46
O
Ph
P
Ph
O
OMe
Methyl 4-(Diphenylphosphoryl)benzoate (**3i**)
11000
10000
9000
8000
7000
6000
5000
4000
3000
2000
1000
0
70
160
150
140
130
120
110
100
90
80
70
60
50
40
30
20
10
0
f1 (ppm)

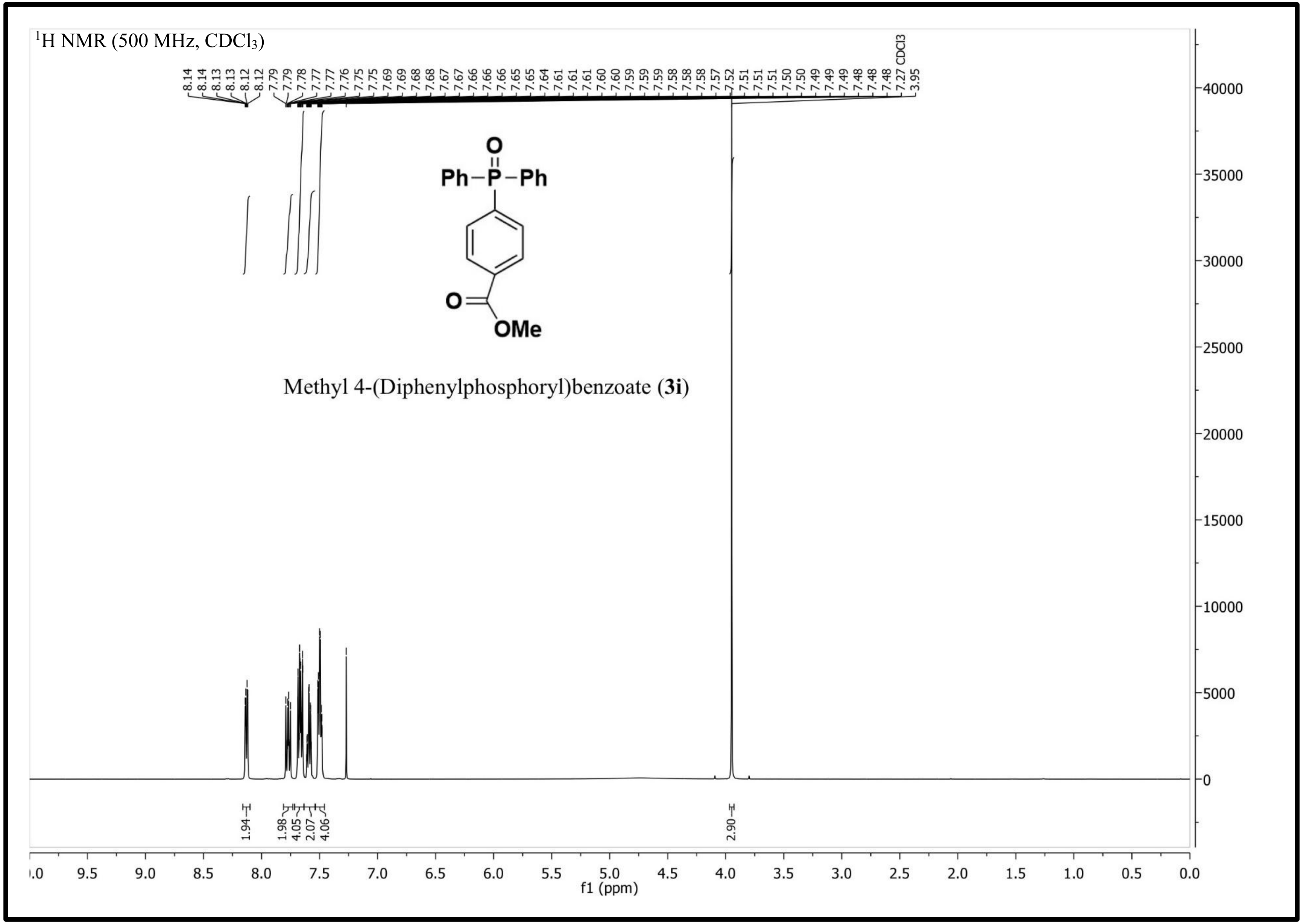

^{1}H NMR (500 MHz, CDCl$_3$)
Ph–P–Ph
O
OMe
Methyl 4-(Diphenylphosphoryl)benzoate (**3i**)
f1 (ppm)

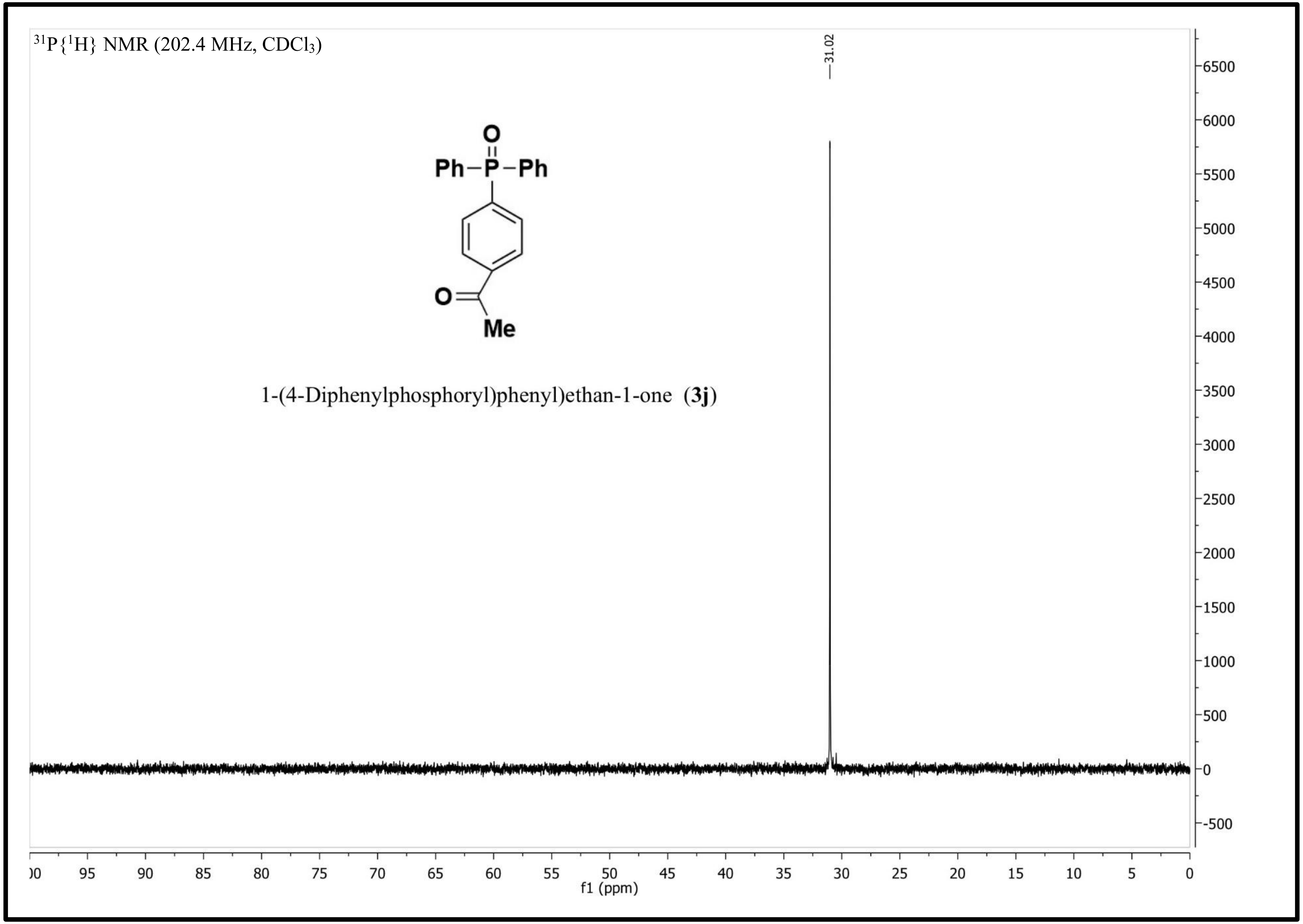

31P{1H} NMR (202.4 MHz, CDCl3)
31.02
Ph–P–Ph
O
O
Me
1-(4-Diphenylphosphoryl)phenyl)ethan-1-one (3j)
f1 (ppm)
95 90 85 80 75 70 65 60 55 50 45 40 35 30 25 20 15 10 5 0
6500 6000 5500 5000 4500 4000 3500 3000 2500 2000 1500 1000 500 0 -500

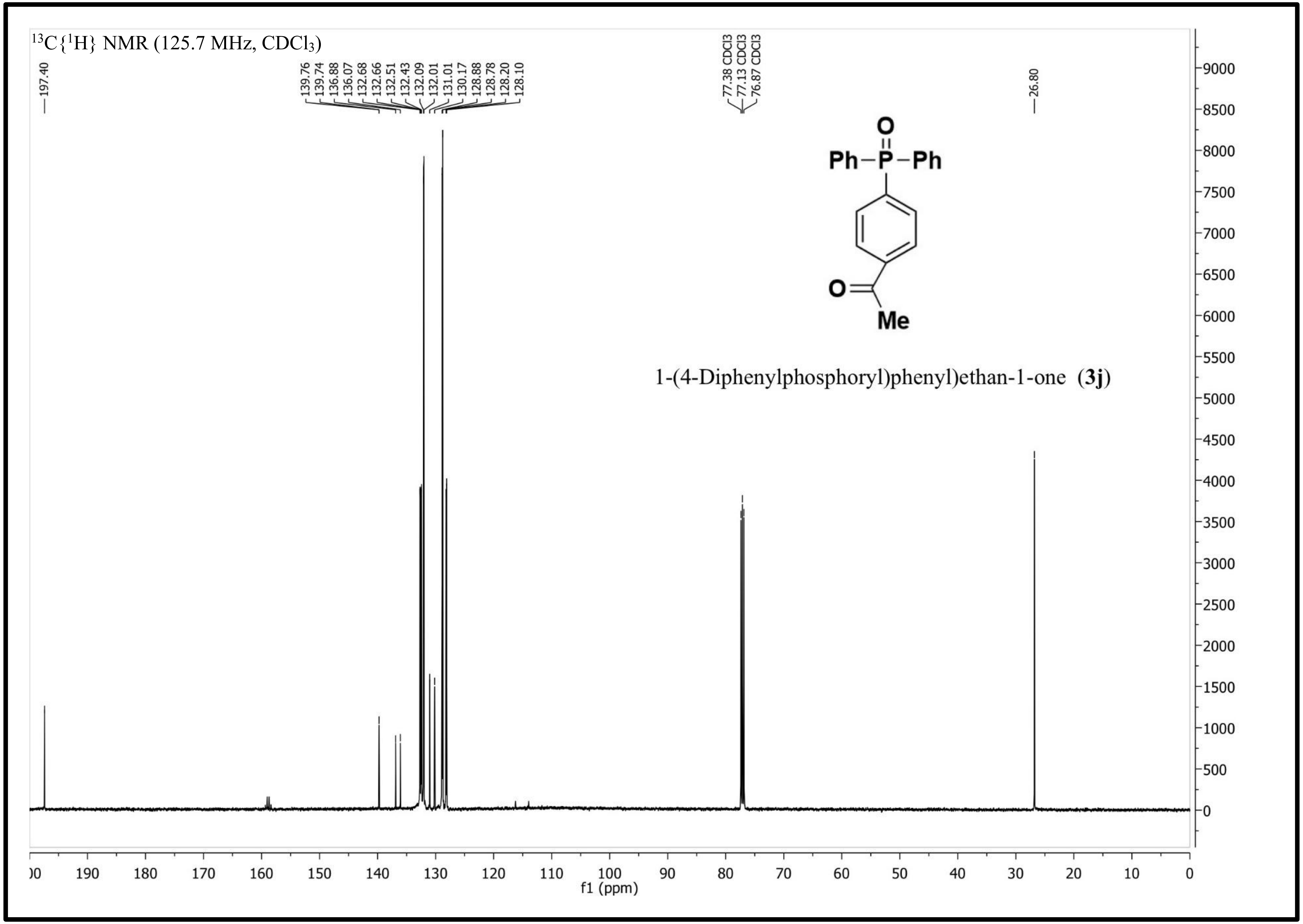

$^{13}C\{^1H\}$ NMR (125.7 MHz, CDCl$_3$)
197.40
139.76 139.74 136.88 136.07 132.68 132.66 132.51 132.43 132.09 132.01 131.01 130.17 128.88 128.78 128.20 128.10
77.38 CDCl3 77.13 CDCl3 76.87 CDCl3
26.80
Ph–P–Ph
O
Me
1-(4-Diphenylphosphoryl)phenyl)ethan-1-one (**3j**)
f1 (ppm)

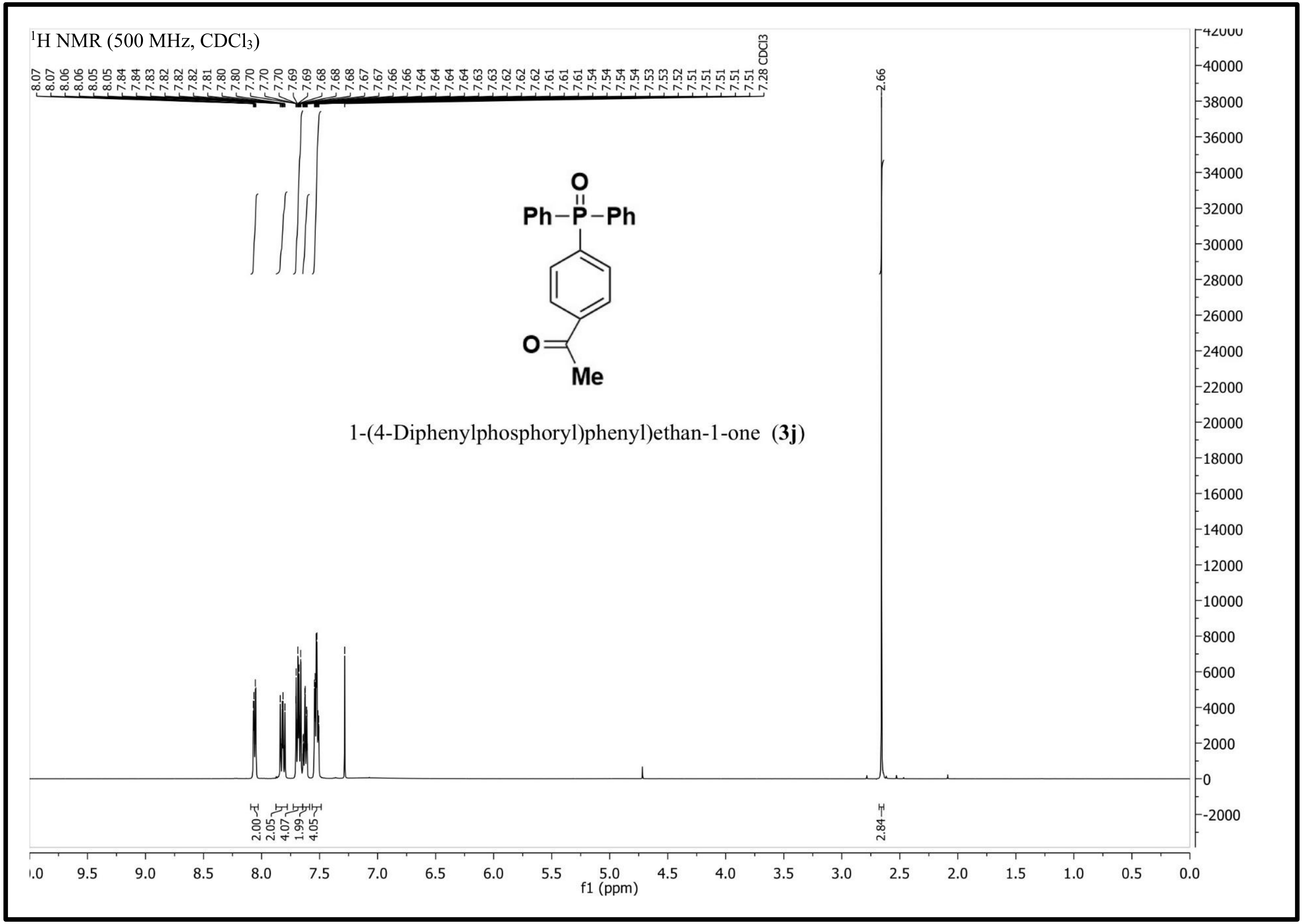

1-(4-Diphenylphosphoryl)phenyl)ethan-1-one (**3j**)

$^{31}P\{^{1}H\}$ NMR (202.4 MHz, $CDCl_3$)

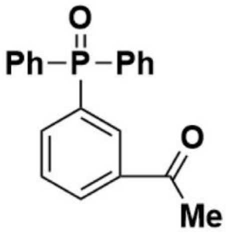

1-(3-Diphenylphosphoryl)phenyl)ethan-1-one (**3k**)

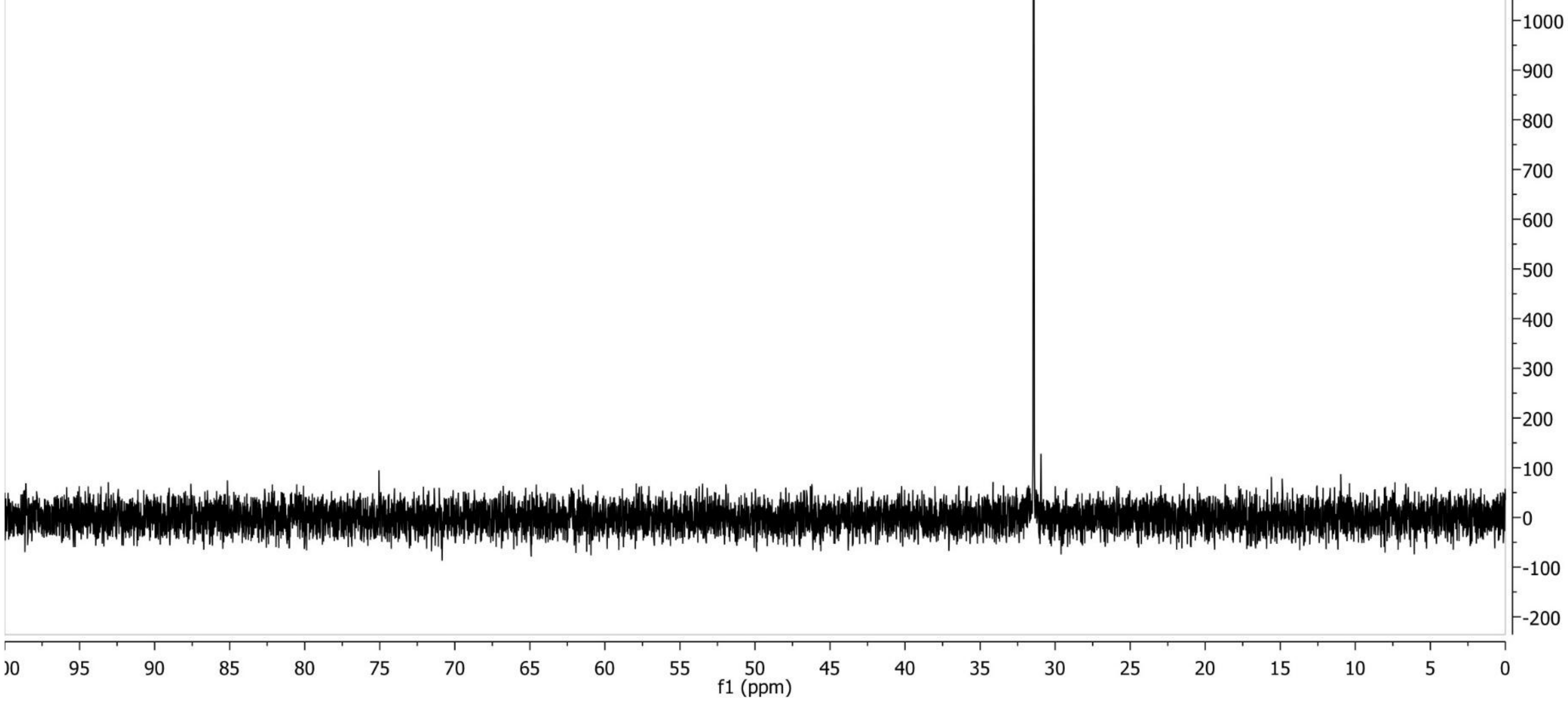

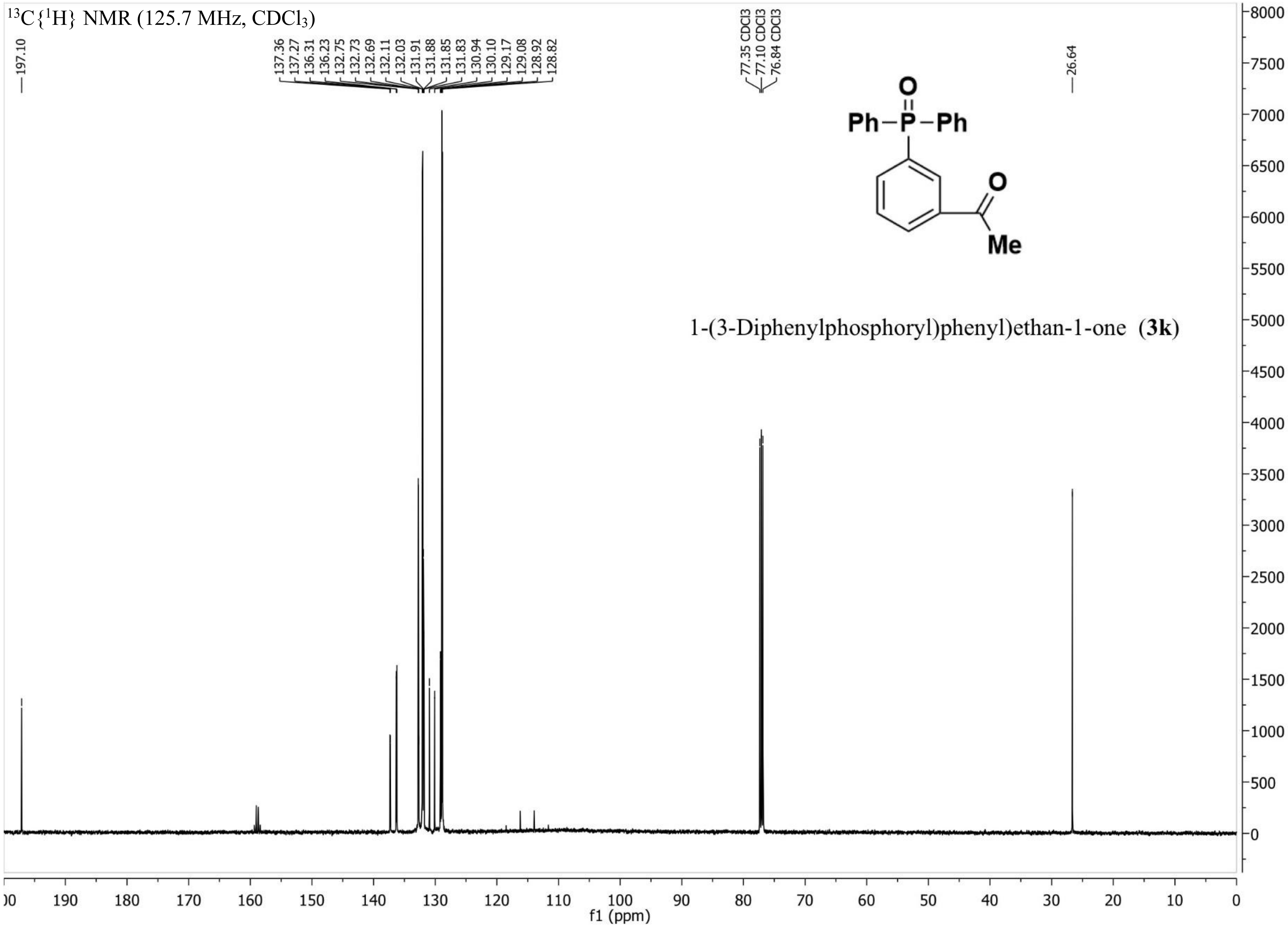

$^{13}C\{^1H\}$ NMR (125.7 MHz, CDCl$_3$)
197.10
137.36
137.27
136.31
136.23
132.75
132.73
132.69
132.11
132.03
131.91
131.88
131.85
131.83
130.94
130.10
129.17
129.08
128.92
128.82
77.35 CDCl3
77.10 CDCl3
76.84 CDCl3
26.64
Ph–P–Ph
O
Me
1-(3-Diphenylphosphoryl)phenyl)ethan-1-one (**3k**)
f1 (ppm)

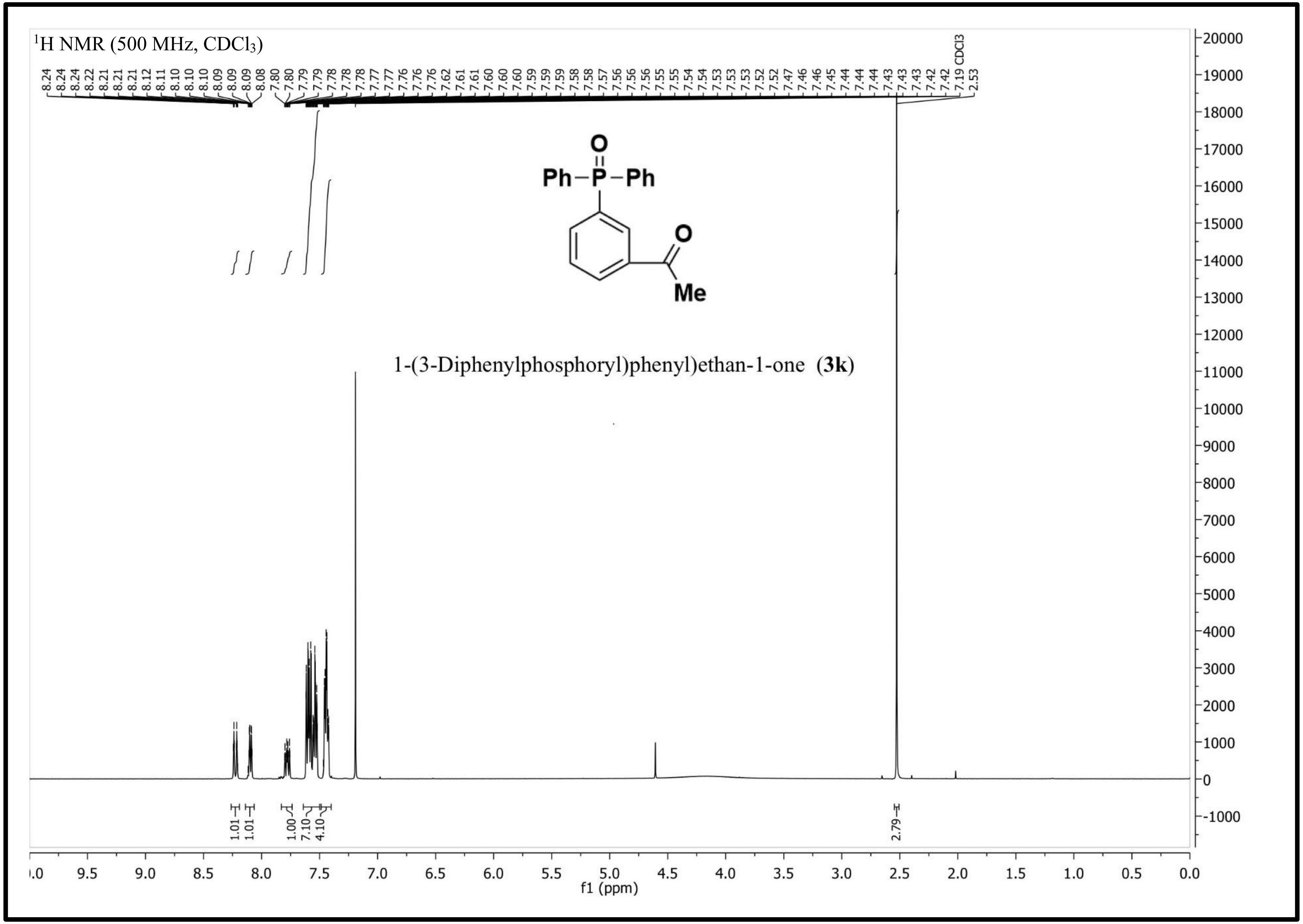

1-(3-Diphenylphosphoryl)phenyl)ethan-1-one (**3k**)

Table S1–S4 Containing the Computed Row Data

Table S1. Computed energies (*E*), zero point energies (*ZPE*), internal energies (*U*), enthalpies (*H*) and Gibbs free energies (*G*) given in Hartree as well as entropies (*S*) given in J mol^{-1} K^{-1} at M06-2X/6-31G(d,p)[PCM(MeCN)] basis set with the consideration of PCM solvent method using the parameter set of water for small molecules and ions used for the calculations.

	E	ZPE	U	H	G	S
PPh_2OH	-880.23905399	-880.040081	-880.027897	-880.026953	-880.080126	111.913
PPh_2OH	-880.23843080	-880.039655	-880.027304	-880.026360	-880.080452	113.847
PPh_2HO	-880.24134257	-880.042809	-880.030989	-880.030045	-880.082996	111.446
PhCl	-691.71717884	-691.625295	-691.619824	-691.618880	-691.655056	76.138
PhBr	-2803.32937318	-2803.237877	-2803.232218	-2803.231274	-2803.268707	78.784
Cl^-	-460.33265000	-460.332650	-460.331230	-460.330290	-460.347670	36.586
Br^-	-2571.94193200	-2571.941932	-2571.940516	-2571.939572	-2571.958108	39.012
KCO_3^-	-863.76934106	-863.753673	-863.748671	-863.747727	-863.783499	75.288
$KHCO_3$	-864.29415624	-864.266014	-864.260513	-864.259569	-864.296571	77.876
Et_3N	-292.27369716	-292.065707	-292.056513	-292.055569	-292.099271	91.979
Et_3NH	-292.73716038	-292.512637	-292.503641	-292.502697	-292.545527	90.143
Pd_L_2	-1888.33191754	-1887.931662	-1887.903220	-1887.902276	-1887.998667	202.872
Pd_L_2	**-1888.33247796**	**-1887.932818**	**-1887.903997**	**-1887.903053**	**-1888.001292**	**206.761**

Table S2. Computed energies (*E*), zero point energies (*ZPE*), internal energies (*U*), enthalpies (*H*) and Gibbs free energies (*G*) given in Hartree as well as entropies (*S*) given in J mol^{-1} K^{-1} at M06-2X/6-31G(d,p)[PCM(MeCN)] basis set with the consideration of PCM solvent method using the parameter set of water for small molecules and ions used for the calculations. ImFrq = imaginary frequency of the TS.

	E	ZPE	U	H	G	S	ImFrq
A(Cl)	-2580.06193094	-2579.569005	-2579.533082	-2579.532137	-2579.643583	234.557	
TS1A(Cl)	-2580.02700656	-2579.535811	-2579.500499	-2579.499555	-2579.607197	226.553	
B(Cl)	-2580.11040161	-2579.616516	-2579.581408	-2579.580464	-2579.689512	229.512	
C(Cl)	-2119.72355786	-2119.230654	-2119.197390	-2119.196446	-2119.299155	216.169	
Pd_L_2–H^+	-1887.85235798	-1887.464669	-1887.436454	-1887.435510	-1887.531305	201.618	
A–H^+(Cl)	-2579.58160900	-2579.100312	-2579.065277	-2579.064332	-2579.172370	227.384	
TS1B(Cl)	-2579.54216400	-2579.063464	-2579.028664	-2579.027720	-2579.134568	224.882	-327
B–H^+(Cl)	**-2579.63458117**	**-2579.152327**	**-2579.117919**	**-2579.116975**	**-2579.222655**	**222.422**	
C–H^+(Cl)	-2119.26497019	-2118.784231	-2118.751275	-2118.750331	-2118.853604	217.357	

Table S3. Computed energies (*E*), zero point energies (*ZPE*), internal energies (*U*), enthalpies (*H*) and Gibbs free energies (*G*) given in Hartree as well as entropies (*S*) given in J mol^{-1} K^{-1} at M06-2X/6-31G(d,p)[PCM(MeCN)] basis set with the consideration of PCM solvent method using the parameter set of water for small molecules and ions used for the calculations. ImFrq = imaginary frequency of the TS.

	E	ZPE	U	H	G	S	ImFrq
D–H$^+$(Cl)	-2999.56865931	-2998.887735	-2998.842211	-2998.841266	-2998.971403	273.895	
TS2	-2999.53410709	-2998.854175	-2998.808687	-2998.807742	-2998.938712	275.648	-171
Final	-2999.58277959	-2998.900121	-2998.853918	-2998.852974	-2998.986367	280.748	

Table S4. Computed energies (*E*), zero point energies (*ZPE*), internal energies (*U*), enthalpies (*H*) and Gibbs free energies (*G*) given in Hartree as well as entropies (*S*) given in J mol^{-1} K^{-1} at M06-2X/6-31G(d,p)[PCM(MeCN)] basis set with the consideration of PCM solvent method using the parameter set of water for small molecules and ions used for the calculations. ImFrq = imaginary frequency of the TS.

	E	ZPE	U	H	G	S	ImFrq
A(Br)	-4691.37520220	-4690.882805	-4690.846931	-4690.845987	-4690.956518	232.632	
TS1A(Br)	-4691.35093129	-4690.860451	-4690.824808	-4690.823864	-4690.934430	232.706	-261
B(Br)	-4691.43148222	-4690.937879	-4690.902621	-4690.901677	-4691.010717	229.494	
A–H$^+$(Br)	-4690.89672694	-4690.415696	-4690.380522	-4690.379578	-4690.487915	228.015	
TS1B(Br)	-4690.86796541	-4690.389153	-4690.354275	-4690.353331	-4690.460102	224.717	
B–H$^+$(Br)	-4690.95488686	-4690.473051	-4690.438417	-4690.437473	-4690.544283	224.800	

Tables containing XYZ coordinates of computed species at M06-2X/6-31G(d,p)[PCM(MeCN)]

_00baa_PPh2OH_A_b3lyp631dp_PCM062X_.log

Input orientation:

Center Number	Atomic Number	Atomic Type	Coordinates (Angstroms) X	Y	Z
1	15	0	-2.081674	-0.069634	0.618066
2	8	0	-2.193140	-0.022217	2.277946
3	1	0	-3.109093	-0.023621	2.596293
4	6	0	-3.210858	-1.453928	0.176733
5	6	0	-4.597642	-1.402893	0.373676
6	6	0	-2.639033	-2.625004	-0.329831
7	6	0	-5.392190	-2.505089	0.076460
8	1	0	-5.058369	-0.491872	0.748412
9	6	0	-3.434787	-3.728300	-0.635494
10	1	0	-1.564771	-2.672012	-0.488104
11	6	0	-4.810257	-3.668228	-0.429915
12	1	0	-6.465057	-2.457783	0.232649
13	1	0	-2.981592	-4.630684	-1.032588
14	1	0	-5.432157	-4.525445	-0.667060
15	6	0	-3.147072	1.357766	0.158070
16	6	0	-3.372447	2.401211	1.059849
17	6	0	-3.666614	1.454778	-1.139461
18	6	0	-4.119165	3.515382	0.677413
19	1	0	-2.964444	2.341971	2.064503
20	6	0	-4.407139	2.568371	-1.521151
21	1	0	-3.502396	0.648870	-1.851079
22	6	0	-4.636796	3.601385	-0.611663
23	1	0	-4.294401	4.315961	1.389035
24	1	0	-4.810100	2.629589	-2.527016
25	1	0	-5.216694	4.468892	-0.909317

_00bba_PPh2OH_B_b3lyp631dp_PCM062X_.log

Input orientation:

Center Number	Atomic Number	Atomic Type	Coordinates (Angstroms) X	Y	Z
1	15	0	-2.143883	-0.086401	0.928861
2	8	0	-2.607840	0.082086	2.520699
3	6	0	-3.234965	-1.466548	0.397493
4	6	0	-4.456662	-1.720998	1.030534
5	6	0	-2.833180	-2.271601	-0.670692

6	6	0	-5.265600	-2.765115	0.594965
7	1	0	-4.760545	-1.100073	1.868675
8	6	0	-3.652469	-3.308399	-1.118341
9	1	0	-1.874900	-2.092764	-1.152787
10	6	0	-4.866347	-3.556325	-0.483989
11	1	0	-6.210527	-2.962330	1.091435
12	1	0	-3.336542	-3.927164	-1.952018
13	1	0	-5.500778	-4.368370	-0.824450
14	6	0	-3.111113	1.317175	0.238153
15	6	0	-3.518297	2.380688	1.046780
16	6	0	-3.377570	1.360642	-1.135487
17	6	0	-4.194510	3.466223	0.490495
18	1	0	-3.314850	2.348076	2.111988
19	6	0	-4.049164	2.445790	-1.688947
20	1	0	-3.069702	0.536016	-1.774961
21	6	0	-4.460096	3.502230	-0.875675
22	1	0	-4.514255	4.284683	1.127977
23	1	0	-4.255589	2.466714	-2.754284
24	1	0	-4.985174	4.348352	-1.306933
25	1	0	-1.861755	-0.104768	3.103671

_00bca_PPh2HO_b3lyp631dp_PCM062X_.log

Input orientation:

Center Number	Atomic Number	Atomic Type	Coordinates (Angstroms) X	Y	Z
1	15	0	-2.304647	-0.073933	0.830897
2	8	0	-2.006043	0.005955	2.301125
3	6	0	-3.272732	-1.494976	0.277375
4	6	0	-4.652014	-1.535815	0.513809
5	6	0	-2.639230	-2.574003	-0.344093
6	6	0	-5.388201	-2.652412	0.134016
7	1	0	-5.148192	-0.691080	0.984750
8	6	0	-3.380206	-3.689873	-0.726826
9	1	0	-1.569440	-2.543349	-0.532769
10	6	0	-4.751523	-3.728046	-0.486142
11	1	0	-6.457316	-2.683774	0.314928
12	1	0	-2.888505	-4.525595	-1.213072
13	1	0	-5.328549	-4.596888	-0.785500
14	6	0	-3.188905	1.382227	0.220346
15	6	0	-3.345486	2.467834	1.083113
16	6	0	-3.694305	1.439966	-1.082580
17	6	0	-4.009110	3.611985	0.644599
18	1	0	-2.950364	2.402855	2.092354
19	6	0	-4.351258	2.585790	-1.518333
20	1	0	-3.581683	0.590560	-1.751723

21	6	0	-4.508769	3.670084	-0.654063
22	1	0	-4.135673	4.455401	1.315134
23	1	0	-4.744858	2.633138	-2.528120
24	1	0	-5.025097	4.561265	-0.995790
25	1	0	-1.156476	-0.182655	0.019183

_00caa_PhCl_M062X_631dp_PCMmecn.log

Input orientation:

Center Number	Atomic Number	Atomic Type	Coordinates (Angstroms) X	Y	Z
1	6	0	-0.477057	0.456713	0.000063
2	6	0	0.916069	0.455343	0.000524
3	6	0	1.611516	1.662435	-0.000048
4	6	0	0.922927	2.873332	-0.001060
5	6	0	-0.467415	2.851807	-0.001495
6	6	0	-1.181328	1.658521	-0.000955
7	1	0	-1.024525	-0.479865	0.000498
8	1	0	1.458290	-0.483932	0.001317
9	1	0	2.696351	1.668238	0.000305
10	1	0	1.453174	3.818900	-0.001514
11	1	0	-2.265336	1.672227	-0.001327
12	17	0	-1.343177	4.368722	-0.002792

_00cba_PhBr_M062X_631dp_PCMmecn.log

Input orientation:

Center Number	Atomic Number	Atomic Type	Coordinates (Angstroms) X	Y	Z
1	6	0	-0.477412	0.456853	0.000063
2	6	0	0.915801	0.455815	0.000522
3	6	0	1.611569	1.662814	-0.000041
4	6	0	0.922591	2.873659	-0.001070
5	6	0	-0.467374	2.851726	-0.001512
6	6	0	-1.181449	1.658960	-0.000959
7	1	0	-1.025188	-0.479594	0.000505
8	1	0	1.458038	-0.483491	0.001322
9	1	0	2.696443	1.668965	0.000319
10	1	0	1.454269	3.818289	-0.001524
11	1	0	-2.265359	1.670963	-0.001329
12	35	0	-1.417473	4.497365	-0.002910

_01aaa_Br-_M062X_631dp_PCMmecn.log

Input orientation:

Center Number	Atomic Number	Atomic Type	Coordinates (Angstroms) X	Y	Z
1	35	0	-0.406699	0.430622	0.000000

_01baa_Cl-_M062X_631dp_PCMmecn.log

Input orientation:

Center Number	Atomic Number	Atomic Type	Coordinates (Angstroms) X	Y	Z
1	17	0	-0.406699	0.430622	0.000000

00000gda_KCO3-_M062X_631dp_PCMacn.log

Input orientation:

Center Number	Atomic Number	Atomic Type	Coordinates (Angstroms) X	Y	Z
1	6	0	-0.024204	0.027323	0.011145
2	8	0	-0.021833	-1.277701	0.040043
3	8	0	-1.122255	0.675489	-0.050766
4	8	0	1.119319	0.655955	0.044072
5	19	0	2.494486	-1.458761	-0.158296

00000gfa_KHCO3_M062X_631dp_PCMacn.log

Input orientation:

Center Number	Atomic Number	Atomic Type	Coordinates (Angstroms) X	Y	Z
1	6	0	-0.026864	0.090664	-0.018543
2	8	0	-1.046509	0.809987	-0.025720
3	8	0	1.176889	0.443381	-0.049047
4	8	0	-0.267159	-1.268298	0.029229
5	1	0	0.604208	-1.685108	0.028958
6	19	0	0.456188	2.987121	-0.119388

000bbb_TEA_M06_2X_631dp_PCMMeCN.log

Input orientation:

Center Number	Atomic Number	Atomic Type	Coordinates (Angstroms) X	Y	Z
1	6	0	0.137200	1.416743	0.858801
2	6	0	-0.743613	2.470640	0.194628
3	1	0	-1.787207	2.397876	0.510014
4	6	0	0.949028	-0.859832	0.674804
5	6	0	1.234098	-1.163158	2.149102
6	1	0	0.386767	-1.661885	2.629392
7	1	0	0.755900	-1.796954	0.142801
8	1	0	1.843948	-0.426242	0.214877
9	1	0	2.100555	-1.824345	2.234142
10	1	0	1.452293	-0.250476	2.711151
11	1	0	0.052169	1.509619	1.957129
12	1	0	1.183201	1.624951	0.607438
13	1	0	-0.391928	3.471808	0.457047
14	1	0	-0.704834	2.362000	-0.892522
15	6	0	-1.440771	-0.407848	0.929730
16	6	0	-1.818962	-1.801297	0.442517
17	1	0	-2.209702	0.295587	0.596569
18	1	0	-1.458385	-0.383461	2.035507
19	1	0	-2.854441	-2.021613	0.712679
20	1	0	-1.725425	-1.861294	-0.645654
21	1	0	-1.189195	-2.578193	0.884312
22	7	0	-0.158869	0.059086	0.403181

000cbb_TEA+H+_M06_2X_631dp_PCMMeCN.log

Input orientation:

Center Number	Atomic Number	Atomic Type	Coordinates (Angstroms) X	Y	Z
1	6	0	0.203471	1.460891	0.800991
2	6	0	-0.788731	2.485128	0.279559
3	1	0	-1.763255	2.406072	0.762484
4	6	0	1.003204	-0.902505	0.687547
5	6	0	1.161232	-1.162671	2.173938
6	1	0	0.279116	-1.637304	2.608573
7	1	0	0.795492	-1.823249	0.142037
8	1	0	1.904519	-0.456975	0.264082

9	1	0	2.000708	-1.848537	2.300679
10	1	0	1.390600	-0.254443	2.733674
11	1	0	0.231362	1.449703	1.891152
12	1	0	1.207897	1.666962	0.427649
13	1	0	-0.391780	3.478323	0.495941
14	1	0	-0.915759	2.405475	-0.803516
15	6	0	-1.466958	-0.398525	0.862449
16	6	0	-1.800343	-1.821704	0.454792
17	1	0	-2.195184	0.295327	0.443840
18	1	0	-1.453675	-0.270061	1.946624
19	1	0	-2.835966	-2.018879	0.736307
20	1	0	-1.716148	-1.956331	-0.626719
21	1	0	-1.172004	-2.559617	0.956294
22	7	0	-0.119885	0.055147	0.359224
23	1	0	-0.178393	0.077266	-0.665022

001aaa_Pd_PHPh22_b3lyp631dp_PCM062X_.log

Standard orientation:

Center Number	Atomic Number	Atomic Type	Coordinates (Angstroms) X	Y	Z
1	46	0	-0.009876	0.537686	0.044230
2	15	0	2.204837	0.468793	0.753472
3	15	0	-2.214869	0.434007	-0.689223
4	8	0	-2.529453	1.218300	-2.113089
5	1	0	-3.430704	1.070716	-2.439869
6	8	0	2.521243	1.137099	2.234786
7	1	0	3.468897	1.186637	2.435507
8	6	0	3.415265	1.295099	-0.344051
9	6	0	4.789004	1.030455	-0.271520
10	6	0	2.947037	2.253679	-1.248569
11	6	0	5.678862	1.721223	-1.087839
12	1	0	5.162397	0.275666	0.416650
13	6	0	3.838573	2.941316	-2.069834
14	1	0	1.878787	2.452442	-1.308221
15	6	0	5.203184	2.675485	-1.987867
16	1	0	6.741870	1.512064	-1.028330
17	1	0	3.468318	3.680635	-2.772334
18	1	0	5.898586	3.208404	-2.628216
19	6	0	2.943123	-1.199529	0.908715
20	6	0	3.361042	-1.707746	2.139770
21	6	0	3.039738	-2.000700	-0.236689
22	6	0	3.882971	-2.999259	2.223328
23	1	0	3.275764	-1.099186	3.034830
24	6	0	3.561184	-3.286374	-0.151039
25	1	0	2.709268	-1.613042	-1.198550

26	6	0	3.984774	-3.787681	1.081327
27	1	0	4.208462	-3.386498	3.183399
28	1	0	3.637687	-3.899009	-1.043389
29	1	0	4.389750	-4.792069	1.148118
30	6	0	-3.488172	1.129151	0.428530
31	6	0	-3.700636	0.522542	1.673446
32	6	0	-4.206789	2.278181	0.092938
33	6	0	-4.628863	1.052624	2.562219
34	1	0	-3.141638	-0.371046	1.943912
35	6	0	-5.137746	2.808340	0.986981
36	1	0	-4.038630	2.762335	-0.864227
37	6	0	-5.350157	2.197685	2.219119
38	1	0	-4.791414	0.574257	3.522544
39	1	0	-5.695005	3.699644	0.717429
40	1	0	-6.073515	2.611650	2.913846
41	6	0	-2.876434	-1.248585	-0.978675
42	6	0	-4.250344	-1.496130	-1.096981
43	6	0	-1.972709	-2.305527	-1.126753
44	6	0	-4.709375	-2.781028	-1.367605
45	1	0	-4.963111	-0.685209	-0.965684
46	6	0	-2.433735	-3.593883	-1.391759
47	1	0	-0.906380	-2.112195	-1.025583
48	6	0	-3.800721	-3.830037	-1.513426
49	1	0	-5.774432	-2.967570	-1.457739
50	1	0	-1.727455	-4.410275	-1.500768
51	1	0	-4.161487	-4.832905	-1.717804

001baa_Pd_PHPh22_b3lyp631dp_PCM062X_.log

Standard orientation:

Center Number	Atomic Number	Atomic Type	Coordinates (Angstroms) X	Y	Z
1	46	0	-0.003404	-0.584896	0.073791
2	15	0	2.198948	-0.514847	-0.671520
3	15	0	-2.246945	-0.520767	0.697288
4	8	0	-2.652246	-1.417239	2.028943
5	1	0	-3.566885	-1.278462	2.320232
6	8	0	2.599539	-1.339904	-2.054694
7	1	0	1.797122	-1.582693	-2.534733
8	6	0	3.476125	-1.171986	0.458784
9	6	0	4.841126	-0.951161	0.238871
10	6	0	3.070300	-1.941237	1.553103
11	6	0	5.785170	-1.500855	1.099590
12	1	0	5.162988	-0.343808	-0.603175
13	6	0	4.017114	-2.486056	2.419621
14	1	0	2.008697	-2.109497	1.722773

15	6	0	5.372847	-2.266569	2.191501
16	1	0	6.842219	-1.328673	0.924944
17	1	0	3.695842	-3.079045	3.269598
18	1	0	6.111296	-2.688839	2.865410
19	6	0	2.861380	1.148262	-1.048217
20	6	0	3.593285	1.407417	-2.209355
21	6	0	2.618040	2.183860	-0.138567
22	6	0	4.084340	2.689858	-2.452660
23	1	0	3.772325	0.604506	-2.917602
24	6	0	3.116124	3.460508	-0.379758
25	1	0	2.032062	1.988347	0.758008
26	6	0	3.849525	3.714626	-1.539115
27	1	0	4.651182	2.887267	-3.356875
28	1	0	2.925840	4.258828	0.330204
29	1	0	4.231623	4.711902	-1.731410
30	6	0	-3.450083	-1.105846	-0.552591
31	6	0	-3.567156	-0.401054	-1.757881
32	6	0	-4.208181	-2.261497	-0.356306
33	6	0	-4.440986	-0.841163	-2.745311
34	1	0	-2.976888	0.498845	-1.919321
35	6	0	-5.084000	-2.701639	-1.349557
36	1	0	-4.114155	-2.821139	0.569311
37	6	0	-5.202077	-1.993747	-2.541690
38	1	0	-4.530150	-0.286974	-3.673980
39	1	0	-5.672391	-3.599076	-1.188286
40	1	0	-5.882834	-2.337785	-3.313341
41	6	0	-2.914638	1.139033	1.086856
42	6	0	-4.290136	1.404648	1.104289
43	6	0	-2.014687	2.156581	1.420277
44	6	0	-4.754787	2.667267	1.458328
45	1	0	-4.998859	0.627272	0.828847
46	6	0	-2.480733	3.422375	1.770237
47	1	0	-0.946227	1.951109	1.396504
48	6	0	-3.849961	3.676137	1.790354
49	1	0	-5.821037	2.867904	1.469246
50	1	0	-1.776680	4.208035	2.023695
51	1	0	-4.214623	4.661945	2.060273

001baa_Pd_PHPh22_-H+_b3lyp631dp_PCM062X_.log

Input orientation:

Center Number	Atomic Number	Atomic Type	Coordinates (Angstroms) X	Y	Z
1	46	0	-0.002657	-0.001744	0.996112
2	15	0	2.277636	0.052643	0.891348
3	15	0	-2.395517	-0.083055	1.086057

4	8	0	-3.181768	-0.216067	2.407979
5	8	0	3.158467	0.200249	2.295320
6	1	0	2.579945	0.046984	3.053384
7	6	0	3.037288	1.411659	-0.074665
8	6	0	4.410501	1.444471	-0.346476
9	6	0	2.222926	2.453573	-0.526302
10	6	0	4.959323	2.509289	-1.052753
11	1	0	5.048014	0.631475	-0.007958
12	6	0	2.771890	3.517215	-1.242519
13	1	0	1.156266	2.424641	-0.311901
14	6	0	4.138906	3.545258	-1.503455
15	1	0	6.024616	2.531477	-1.258706
16	1	0	2.133177	4.321389	-1.593222
17	1	0	4.568336	4.372149	-2.060005
18	6	0	3.096617	-1.404086	0.138057
19	6	0	4.215230	-2.010652	0.714538
20	6	0	2.568511	-1.921876	-1.050473
21	6	0	4.803909	-3.117234	0.102599
22	1	0	4.616746	-1.615062	1.642314
23	6	0	3.162176	-3.020628	-1.663996
24	1	0	1.683614	-1.464522	-1.490263
25	6	0	4.281370	-3.620892	-1.086175
26	1	0	5.671444	-3.585973	0.556352
27	1	0	2.747708	-3.415345	-2.586071
28	1	0	4.739911	-4.483185	-1.559307
29	6	0	-3.045980	-1.405166	-0.050748
30	6	0	-2.415197	-1.727699	-1.256835
31	6	0	-4.195576	-2.106938	0.318331
32	6	0	-2.934615	-2.718545	-2.087510
33	1	0	-1.504490	-1.200867	-1.541474
34	6	0	-4.714702	-3.105274	-0.505305
35	1	0	-4.667693	-1.856132	1.264872
36	6	0	-4.086500	-3.410854	-1.711653
37	1	0	-2.437947	-2.959084	-3.022963
38	1	0	-5.608225	-3.646684	-0.207835
39	1	0	-4.486859	-4.189564	-2.353448
40	6	0	-3.137611	1.362699	0.178766
41	6	0	-4.303363	1.946178	0.679431
42	6	0	-2.559045	1.893498	-0.979052
43	6	0	-4.888397	3.034242	0.032200
44	1	0	-4.735539	1.531423	1.586724
45	6	0	-3.144270	2.974925	-1.634039
46	1	0	-1.637518	1.457557	-1.364761
47	6	0	-4.311316	3.548873	-1.127536
48	1	0	-5.793924	3.482417	0.431168
49	1	0	-2.687992	3.377340	-2.533762
50	1	0	-4.763324	4.397414	-1.631744

001eaa_Pd_PHPh22_PhCl_complex_b3lyp631dp_PCM062X_f.log

Standard orientation:

Center Number	Atomic Number	Atomic Type	Coordinates (Angstroms) X	Y	Z
1	46	0	-0.472804	-0.990473	-0.905818
2	15	0	1.686793	-1.866986	-0.720209
3	15	0	-2.611241	-0.096402	-1.044483
4	8	0	-3.125505	0.298734	-2.567783
5	1	0	-4.018749	0.676559	-2.585581
6	8	0	2.019597	-3.363768	-1.358088
7	1	0	1.309423	-3.626154	-1.958107
8	6	0	2.267568	-2.109391	0.997306
9	6	0	3.608045	-1.980527	1.374036
10	6	0	1.308267	-2.422065	1.967621
11	6	0	3.982430	-2.164341	2.703096
12	1	0	4.358409	-1.722898	0.631969
13	6	0	1.683179	-2.612558	3.295408
14	1	0	0.262059	-2.500002	1.677422
15	6	0	3.021728	-2.479962	3.663608
16	1	0	5.023332	-2.057528	2.990740
17	1	0	0.933225	-2.852236	4.042123
18	1	0	3.315446	-2.616978	4.699314
19	6	0	3.028486	-0.859761	-1.440281
20	6	0	4.058386	-1.421616	-2.198290
21	6	0	3.009248	0.520307	-1.209483
22	6	0	5.063485	-0.606345	-2.717907
23	1	0	4.067127	-2.492047	-2.378301
24	6	0	4.019225	1.329360	-1.720878
25	1	0	2.201358	0.959107	-0.626896
26	6	0	5.046420	0.766392	-2.478593
27	1	0	5.861204	-1.044491	-3.309193
28	1	0	3.998504	2.398746	-1.532274
29	1	0	5.830237	1.397485	-2.884865
30	6	0	-2.819751	1.487045	-0.148642
31	6	0	-2.898134	1.489598	1.250168
32	6	0	-2.768850	2.703333	-0.835778
33	6	0	-2.926032	2.692364	1.948942
34	1	0	-2.939104	0.547848	1.793310
35	6	0	-2.801313	3.907875	-0.132771
36	1	0	-2.697674	2.708791	-1.919377
37	6	0	-2.875594	3.904082	1.258053
38	1	0	-2.985634	2.685719	3.032372
39	1	0	-2.761694	4.847858	-0.673557
40	1	0	-2.893356	4.841436	1.804045
41	6	0	-3.988168	-1.087552	-0.360223
42	6	0	-5.249790	-0.531814	-0.109545
43	6	0	-3.776202	-2.447999	-0.116877

44	6	0	-6.283740	-1.329448	0.368887
45	1	0	-5.420530	0.529315	-0.275592
46	6	0	-4.810966	-3.245727	0.369473
47	1	0	-2.793639	-2.875448	-0.306502
48	6	0	-6.063304	-2.686450	0.609693
49	1	0	-7.258826	-0.894254	0.561340
50	1	0	-4.638812	-4.299845	0.560118
51	1	0	-6.869804	-3.305746	0.988762
52	6	0	1.568401	3.222365	0.702352
53	6	0	2.836837	3.010260	1.232932
54	6	0	3.048609	1.887016	2.030729
55	6	0	2.007386	0.999014	2.293472
56	6	0	0.743506	1.233452	1.753127
57	6	0	0.512991	2.350975	0.951317
58	1	0	3.640744	3.706384	1.020443
59	1	0	4.035715	1.705536	2.443464
60	1	0	2.182197	0.121860	2.908754
61	1	0	-0.073595	0.541873	1.942313
62	1	0	-0.465751	2.540068	0.523300
63	17	0	1.291003	4.617916	-0.319429

002aca_Pd_PHPh22_Ph_Cl_b3lyp631dp_PCM062X_.log

Standard orientation:

Center Number	Atomic Number	Atomic Type	Coordinates (Angstroms) X	Y	Z
1	46	0	-0.012681	-0.145742	-0.998758
2	15	0	-2.356637	-0.178624	-0.876569
3	15	0	2.337043	-0.158763	-0.903825
4	8	0	2.968337	-0.345160	-2.403415
5	1	0	2.244163	-0.557161	-3.023070
6	8	0	-3.002083	-0.400062	-2.365140
7	6	0	-3.221372	1.311553	-0.312930
8	6	0	-3.260487	1.619082	1.052938
9	6	0	-3.781562	2.191573	-1.242367
10	6	0	-3.862263	2.797404	1.481326
11	1	0	-2.817870	0.940842	1.779053
12	6	0	-4.385136	3.369779	-0.806278
13	1	0	-3.753648	1.946360	-2.298976
14	6	0	-4.424641	3.673124	0.552420
15	1	0	-3.890380	3.033005	2.540037
16	1	0	-4.825779	4.048186	-1.529265
17	1	0	-4.894077	4.591551	0.889376
18	6	0	-3.044648	-1.513834	0.146764
19	6	0	-4.374669	-1.493640	0.583954
20	6	0	-2.232032	-2.610330	0.453072

21	6	0	-4.880985	-2.560602	1.319219
22	1	0	-5.009976	-0.643015	0.354378
23	6	0	-2.740658	-3.676607	1.190634
24	1	0	-1.199601	-2.627030	0.112102
25	6	0	-4.064147	-3.649535	1.624075
26	1	0	-5.911641	-2.542332	1.657182
27	1	0	-2.104906	-4.522955	1.427992
28	1	0	-4.461099	-4.477514	2.202265
29	6	0	3.014899	-1.534247	0.074879
30	6	0	4.287902	-1.487637	0.653806
31	6	0	2.240083	-2.693355	0.200769
32	6	0	4.774195	-2.588187	1.353949
33	1	0	4.895427	-0.592290	0.562849
34	6	0	2.729220	-3.793202	0.899648
35	1	0	1.251882	-2.731332	-0.253032
36	6	0	3.995633	-3.737870	1.478844
37	1	0	5.760683	-2.548063	1.803500
38	1	0	2.122533	-4.687358	0.995855
39	1	0	4.376444	-4.591690	2.029634
40	6	0	3.216256	1.309710	-0.305502
41	6	0	3.867832	2.154011	-1.208118
42	6	0	3.191402	1.630018	1.058583
43	6	0	4.496544	3.309916	-0.748030
44	1	0	3.888549	1.899012	-2.262341
45	6	0	3.819846	2.785526	1.509909
46	1	0	2.679374	0.981225	1.766239
47	6	0	4.472237	3.626222	0.607796
48	1	0	5.006218	3.960752	-1.450700
49	1	0	3.796694	3.030869	2.566634
50	1	0	4.961268	4.527237	0.963407
51	6	0	0.010631	0.556451	0.863292
52	6	0	0.123653	-0.285486	1.973520
53	6	0	-0.054367	1.939320	1.063551
54	6	0	0.176236	0.250489	3.263237
55	1	0	0.183094	-1.363254	1.843162
56	6	0	0.000062	2.472626	2.350607
57	1	0	-0.147694	2.608876	0.211503
58	6	0	0.117517	1.629809	3.454551
59	1	0	0.266995	-0.414687	4.116916
60	1	0	-0.049460	3.548791	2.488713
61	1	0	0.161332	2.043946	4.456719
62	17	0	-0.020018	-0.972078	-3.393715
63	1	0	-2.284114	-0.622408	-2.988472

002baa_Pd_PHPh22_Ph_Cl_b3lyp631dp_PCM062X_f.log

Standard orientation:

Center Number	Atomic Number	Atomic Type	Coordinates (Angstroms) X	Y	Z
1	46	0	0.010418	0.107953	0.332543
2	15	0	-2.335087	-0.032456	0.334211
3	15	0	2.352889	0.036134	0.416748
4	8	0	2.959574	-0.457559	1.859306
5	1	0	3.929434	-0.472452	1.876256
6	8	0	-3.023169	-0.640858	1.692484
7	1	0	-2.345885	-0.785332	2.375426
8	6	0	-2.965516	-1.170022	-0.931316
9	6	0	-3.055157	-0.760294	-2.267155
10	6	0	-3.270723	-2.489061	-0.583071
11	6	0	-3.457850	-1.665983	-3.243728
12	1	0	-2.811185	0.263286	-2.537619
13	6	0	-3.673424	-3.390584	-1.566253
14	1	0	-3.203751	-2.801252	0.454385
15	6	0	-3.766530	-2.980661	-2.894300
16	1	0	-3.533301	-1.345306	-4.277430
17	1	0	-3.916775	-4.412015	-1.293030
18	1	0	-4.081276	-3.684267	-3.657997
19	6	0	-3.227957	1.524430	0.063726
20	6	0	-4.570059	1.531356	-0.334201
21	6	0	-2.574638	2.731756	0.330300
22	6	0	-5.249795	2.738507	-0.462503
23	1	0	-5.079274	0.595772	-0.548053
24	6	0	-3.258356	3.938190	0.201117
25	1	0	-1.528984	2.724095	0.627724
26	6	0	-4.593776	3.940204	-0.195761
27	1	0	-6.289436	2.742916	-0.772279
28	1	0	-2.748419	4.873706	0.404951
29	1	0	-5.125433	4.880406	-0.300341
30	6	0	3.212922	1.595689	0.055618
31	6	0	4.529730	1.621723	-0.418639
32	6	0	2.552640	2.796344	0.339688
33	6	0	5.177517	2.839759	-0.603878
34	1	0	5.046337	0.694460	-0.651138
35	6	0	3.204369	4.012196	0.155281
36	1	0	1.524348	2.773786	0.692215
37	6	0	4.515455	4.032669	-0.317106
38	1	0	6.196926	2.857907	-0.974309
39	1	0	2.688712	4.941256	0.373538
40	1	0	5.022010	4.980726	-0.465519
41	6	0	3.029970	-1.166824	-0.765664
42	6	0	3.403648	-2.438791	-0.321224
43	6	0	3.085650	-0.859370	-2.131241

44	6	0	3.844939	-3.392752	-1.236574
45	1	0	3.352000	-2.680219	0.736267
46	6	0	3.526445	-1.816707	-3.039047
47	1	0	2.785460	0.126053	-2.476991
48	6	0	3.906645	-3.082808	-2.592722
49	1	0	4.140540	-4.376713	-0.888029
50	1	0	3.575976	-1.574455	-4.095401
51	1	0	4.250868	-3.826631	-3.303684
52	6	0	-0.029420	-0.973495	2.000534
53	6	0	0.101635	-2.366185	1.924469
54	6	0	-0.241114	-0.385478	3.254913
55	6	0	0.025551	-3.151374	3.075457
56	1	0	0.264916	-2.844767	0.961322
57	6	0	-0.320196	-1.175533	4.406445
58	1	0	-0.345954	0.693599	3.340136
59	6	0	-0.187237	-2.559185	4.319633
60	1	0	0.128913	-4.229618	2.997317
61	1	0	-0.486874	-0.703157	5.369928
62	1	0	-0.251214	-3.171455	5.213071
63	17	0	0.032448	1.332924	-1.852540

002gea_Pd_PHPh22_Ph_Cl_b3lyp631dp_PCMmecn_TS_CCl.log

Standard orientation:

Center Number	Atomic Number	Atomic Type	Coordinates (Angstroms) X	Y	Z
1	46	0	0.124883	0.534467	-1.324792
2	15	0	-2.316153	0.495822	-1.122944
3	15	0	0.729274	0.314189	1.039544
4	8	0	-0.291585	0.975795	2.168042
5	1	0	-1.012191	1.462730	1.739465
6	8	0	-3.369294	0.735884	-2.380844
7	1	0	-2.885756	1.013130	-3.169651
8	6	0	-2.937623	1.670770	0.137191
9	6	0	-2.218001	2.860448	0.322856
10	6	0	-4.096800	1.446387	0.889827
11	6	0	-2.644357	3.805965	1.255263
12	1	0	-1.315378	3.037788	-0.260529
13	6	0	-4.523253	2.393459	1.816151
14	1	0	-4.658477	0.526305	0.756145
15	6	0	-3.796308	3.569726	2.001904
16	1	0	-2.077023	4.719464	1.398395
17	1	0	-5.420325	2.212593	2.398965
18	1	0	-4.127696	4.300806	2.731890
19	6	0	-2.919468	-1.109175	-0.500192
20	6	0	-2.552838	-1.526239	0.786206

21	6	0	-3.631146	-1.979689	-1.328678
22	6	0	-2.884459	-2.801052	1.232274
23	1	0	-2.007898	-0.855040	1.446494
24	6	0	-3.972141	-3.253283	-0.874213
25	1	0	-3.919534	-1.656340	-2.323600
26	6	0	-3.594913	-3.667579	0.400855
27	1	0	-2.583194	-3.116112	2.226535
28	1	0	-4.530487	-3.922778	-1.520549
29	1	0	-3.854376	-4.662428	0.747836
30	6	0	2.330587	0.982576	1.614513
31	6	0	3.427051	0.175090	1.926559
32	6	0	2.482439	2.375270	1.606894
33	6	0	4.658812	0.753837	2.230356
34	1	0	3.324926	-0.906256	1.923074
35	6	0	3.708823	2.951433	1.921054
36	1	0	1.636910	3.009882	1.349371
37	6	0	4.801268	2.139131	2.228367
38	1	0	5.506802	0.119988	2.469040
39	1	0	3.816012	4.031245	1.918980
40	1	0	5.761079	2.587201	2.463730
41	6	0	0.789580	-1.420898	1.597997
42	6	0	0.376987	-1.793755	2.880924
43	6	0	1.232405	-2.396107	0.698694
44	6	0	0.403689	-3.134461	3.257022
45	1	0	0.019821	-1.035656	3.571419
46	6	0	1.272016	-3.734689	1.084407
47	1	0	1.539993	-2.110998	-0.305314
48	6	0	0.852450	-4.104807	2.360641
49	1	0	0.074495	-3.423316	4.250118
50	1	0	1.620409	-4.486692	0.383440
51	1	0	0.871234	-5.148637	2.656743
52	6	0	1.991001	0.100021	-2.155469
53	6	0	3.197313	0.158238	-1.439653
54	6	0	1.659461	-1.027172	-2.921290
55	6	0	3.992415	-0.976611	-1.385273
56	1	0	3.470622	1.058839	-0.899268
57	6	0	2.479114	-2.159275	-2.844059
58	1	0	0.791114	-1.011408	-3.573534
59	6	0	3.637074	-2.144549	-2.075098
60	1	0	4.898501	-0.951065	-0.786677
61	1	0	2.211332	-3.043258	-3.414692
62	1	0	4.276418	-3.019192	-2.030939
63	17	0	1.458305	1.877768	-3.094660

003aba_Pd_PHPh22-H+_Ph_Cl_b3lyp631dp_PCM062X_.log

Standard orientation:

Center Number	Atomic Number	Atomic Type	Coordinates (Angstroms) X	Y	Z
1	46	0	-0.247060	-0.505886	-0.121145
2	15	0	-2.305695	-0.073343	0.863645
3	15	0	2.038342	-0.728925	-0.933919
4	8	0	2.109843	-1.528510	-2.365546
5	1	0	1.215567	-1.898765	-2.525899
6	8	0	-2.451227	-0.138924	2.383912
7	6	0	-3.625913	-1.154154	0.155157
8	6	0	-4.015610	-1.110636	-1.187599
9	6	0	-4.250821	-2.061460	1.013634
10	6	0	-5.016408	-1.953843	-1.659956
11	1	0	-3.533481	-0.415988	-1.869893
12	6	0	-5.249283	-2.913676	0.540603
13	1	0	-3.953683	-2.078227	2.057995
14	6	0	-5.633873	-2.860336	-0.796486
15	1	0	-5.314697	-1.907979	-2.702878
16	1	0	-5.729556	-3.613833	1.217474
17	1	0	-6.412659	-3.519640	-1.166876
18	6	0	-2.871348	1.574158	0.265238
19	6	0	-3.598716	2.388428	1.135235
20	6	0	-2.574187	2.040284	-1.019755
21	6	0	-4.039174	3.645367	0.722852
22	1	0	-3.805185	2.025842	2.138683
23	6	0	-3.019420	3.292618	-1.436770
24	1	0	-1.971617	1.427564	-1.689579
25	6	0	-3.753393	4.096684	-0.564438
26	1	0	-4.602611	4.274113	1.405539
27	1	0	-2.784490	3.647207	-2.435588
28	1	0	-4.093352	5.076194	-0.885558
29	6	0	3.145573	-1.677308	0.165269
30	6	0	4.536208	-1.523713	0.159292
31	6	0	2.562971	-2.635954	1.003182
32	6	0	5.330886	-2.318357	0.981299
33	1	0	4.997080	-0.780173	-0.484453
34	6	0	3.358998	-3.431705	1.823135
35	1	0	1.480830	-2.754730	1.015136
36	6	0	4.743195	-3.270722	1.812921
37	1	0	6.408839	-2.194330	0.973583
38	1	0	2.900064	-4.170600	2.471638
39	1	0	5.364914	-3.886243	2.455029
40	6	0	2.993549	0.772428	-1.310042
41	6	0	3.270799	1.125411	-2.633366
42	6	0	3.396779	1.617217	-0.267066
43	6	0	3.951759	2.309926	-2.911640

44	1	0	2.959250	0.467833	-3.438121
45	6	0	4.073822	2.798606	-0.551462
46	1	0	3.178787	1.355918	0.766319
47	6	0	4.352402	3.146900	-1.873233
48	1	0	4.169252	2.576357	-3.940907
49	1	0	4.379596	3.449534	0.261520
50	1	0	4.880046	4.069655	-2.091967
51	6	0	0.430412	0.839111	1.190562
52	6	0	1.127725	0.411372	2.330362
53	6	0	0.368461	2.215139	0.937895
54	6	0	1.756634	1.326144	3.176836
55	1	0	1.197769	-0.649543	2.558394
56	6	0	0.997316	3.132111	1.782770
57	1	0	-0.170626	2.583599	0.068656
58	6	0	1.699056	2.692499	2.902957
59	1	0	2.297257	0.967816	4.048571
60	1	0	0.941280	4.193884	1.558551
61	1	0	2.192122	3.404527	3.557368
62	17	0	-0.869116	-2.240057	-1.892189

005caa_Pd_PHPh22_-H+_Ph_Cl_b3lyp631dp_PCM062X_TS_CCl_jo.log

Standard orientation:

Center Number	Atomic Number	Atomic Type	Coordinates (Angstroms) X	Y	Z
1	46	0	0.263922	-0.823757	-0.961461
2	15	0	-2.134638	-0.475096	-1.226457
3	15	0	1.280991	1.326392	-0.624289
4	8	0	0.918164	2.474401	-1.590808
5	8	0	-2.981848	-0.958844	-2.566891
6	1	0	-2.405195	-1.456322	-3.160416
7	6	0	-2.843025	1.201748	-1.025561
8	6	0	-2.601677	1.899058	0.164326
9	6	0	-3.575705	1.810725	-2.046686
10	6	0	-3.096051	3.188923	0.330244
11	1	0	-2.014770	1.443754	0.960770
12	6	0	-4.071674	3.102754	-1.874230
13	1	0	-3.760632	1.270040	-2.968750
14	6	0	-3.833705	3.792568	-0.688135
15	1	0	-2.894828	3.723340	1.253463
16	1	0	-4.643690	3.569629	-2.669863
17	1	0	-4.217598	4.799263	-0.557864
18	6	0	-3.030224	-1.358870	0.110246
19	6	0	-2.442395	-1.466428	1.375640
20	6	0	-4.294338	-1.912877	-0.112746
21	6	0	-3.119627	-2.109107	2.410034

22	1	0	-1.448367	-1.057863	1.550433
23	6	0	-4.962620	-2.566275	0.919952
24	1	0	-4.744976	-1.836896	-1.097719
25	6	0	-4.378603	-2.661908	2.182480
26	1	0	-2.658146	-2.186934	3.389583
27	1	0	-5.941077	-3.000263	0.739891
28	1	0	-4.901595	-3.171286	2.985435
29	6	0	3.125189	1.322862	-0.430516
30	6	0	3.810012	1.399442	0.786088
31	6	0	3.870836	1.112656	-1.599144
32	6	0	5.197211	1.254842	0.836132
33	1	0	3.257631	1.555286	1.708418
34	6	0	5.254667	0.971214	-1.556426
35	1	0	3.352361	1.040459	-2.553700
36	6	0	5.922700	1.034935	-0.332270
37	1	0	5.711255	1.310438	1.791338
38	1	0	5.812819	0.804050	-2.472768
39	1	0	7.000878	0.915859	-0.292134
40	6	0	0.762188	1.828616	1.079945
41	6	0	0.337701	3.137280	1.315601
42	6	0	0.654126	0.884779	2.110747
43	6	0	-0.186847	3.502696	2.555812
44	1	0	0.395106	3.855161	0.501417
45	6	0	0.132034	1.246210	3.350756
46	1	0	0.967246	-0.144896	1.933056
47	6	0	-0.295863	2.556799	3.573694
48	1	0	-0.522071	4.522142	2.725217
49	1	0	0.048907	0.504592	4.140371
50	1	0	-0.715284	2.836241	4.535184
51	6	0	1.727932	-2.067664	-0.182267
52	6	0	2.955212	-1.733368	0.421986
53	6	0	0.798889	-2.874341	0.504441
54	6	0	3.137953	-2.026068	1.762667
55	1	0	3.713748	-1.199131	-0.138951
56	6	0	0.998185	-3.129636	1.867364
57	1	0	-0.055983	-3.297374	-0.017528
58	6	0	2.153661	-2.698376	2.506405
59	1	0	4.059544	-1.708908	2.243315
60	1	0	0.252365	-3.705142	2.408761
61	1	0	2.317637	-2.919991	3.555307
62	17	0	1.915978	-2.666204	-2.184810

006aaa_Pd_PHPh22_-H+_Ph_Cl_b3lyp631dp_PCM062X_SCAN_CCl_vege_f.log

Standard orientation:

Center Number	Atomic Number	Atomic Type	Coordinates (Angstroms) X	Y	Z
1	46	0	0.374450	-1.195209	-0.477313
2	15	0	2.405444	-0.181874	-0.712241
3	15	0	-1.792372	-2.117773	-0.044132
4	8	0	-2.150975	-3.620687	-0.097212
5	8	0	2.888756	0.367864	-2.208871
6	1	0	2.101838	0.578181	-2.730795
7	6	0	3.930393	-1.095735	-0.276664
8	6	0	3.830838	-2.442020	0.081854
9	6	0	5.188553	-0.482194	-0.310632
10	6	0	4.975683	-3.170347	0.406335
11	1	0	2.850665	-2.914030	0.104202
12	6	0	6.330496	-1.210706	0.003138
13	1	0	5.270865	0.567722	-0.580294
14	6	0	6.223543	-2.555357	0.364710
15	1	0	4.891617	-4.215166	0.687168
16	1	0	7.304184	-0.732364	-0.027088
17	1	0	7.115642	-3.120538	0.615004
18	6	0	2.598919	1.342506	0.293311
19	6	0	2.102815	1.348457	1.601843
20	6	0	3.176159	2.503985	-0.227560
21	6	0	2.197058	2.495138	2.385594
22	1	0	1.620309	0.455814	1.996981
23	6	0	3.261606	3.655627	0.554913
24	1	0	3.540480	2.505416	-1.250487
25	6	0	2.773203	3.652666	1.860835
26	1	0	1.805906	2.492554	3.398152
27	1	0	3.704256	4.556877	0.142616
28	1	0	2.834778	4.551347	2.466101
29	6	0	-3.030310	-1.205689	-1.100834
30	6	0	-4.270651	-0.746177	-0.642938
31	6	0	-2.717337	-1.024023	-2.454074
32	6	0	-5.164617	-0.114537	-1.507255
33	1	0	-4.535424	-0.868855	0.404509
34	6	0	-3.609692	-0.404746	-3.327932
35	1	0	-1.749007	-1.362921	-2.821222
36	6	0	-4.836793	0.057241	-2.852316
37	1	0	-6.118246	0.245760	-1.131980
38	1	0	-3.345658	-0.272033	-4.372908
39	1	0	-5.532068	0.550630	-3.524383
40	6	0	-2.333374	-1.497906	1.616026
41	6	0	-3.006811	-2.352065	2.490946
42	6	0	-2.043112	-0.192912	2.031762
43	6	0	-3.392155	-1.912219	3.758571

44	1	0	-3.216957	-3.365979	2.159813
45	6	0	-2.431878	0.252323	3.292192
46	1	0	-1.508703	0.478607	1.358542
47	6	0	-3.105915	-0.609247	4.160675
48	1	0	-3.913840	-2.585243	4.432979
49	1	0	-2.205032	1.269640	3.599171
50	1	0	-3.401514	-0.265877	5.147349
51	6	0	-1.107742	2.470644	-0.942559
52	6	0	-2.466803	2.177456	-0.912452
53	6	0	-0.458833	3.077514	0.126805
54	6	0	-3.196069	2.525459	0.222527
55	1	0	-2.944609	1.683205	-1.752163
56	6	0	-1.204966	3.411796	1.256074
57	1	0	0.603207	3.291861	0.074580
58	6	0	-2.572575	3.148013	1.302086
59	1	0	-4.255821	2.293739	0.257075
60	1	0	-0.705836	3.884388	2.096328
61	1	0	-3.148787	3.415865	2.181557
62	17	0	-0.189592	2.103568	-2.394706

010aca_Pd_PHPh22_Phcat_b3lyp631dp_PCM062X_.log

Standard orientation:

Center Number	Atomic Number	Atomic Type	Coordinates (Angstroms) X	Y	Z
1	46	0	-0.001951	0.120020	-1.315980
2	15	0	2.370148	0.164025	-1.095904
3	15	0	-2.374835	0.154368	-1.098233
4	8	0	-3.028246	0.274953	-2.598112
5	1	0	-3.988529	0.414955	-2.599156
6	8	0	3.022511	0.300041	-2.594876
7	6	0	3.210643	-1.272014	-0.368748
8	6	0	3.182509	-1.478623	1.016549
9	6	0	3.823967	-2.212656	-1.201054
10	6	0	3.773902	-2.613694	1.559709
11	1	0	2.694630	-0.758317	1.670044
12	6	0	4.418072	-3.346361	-0.649605
13	1	0	3.841268	-2.058452	-2.275489
14	6	0	4.393106	-3.546889	0.728080
15	1	0	3.748482	-2.770355	2.632976
16	1	0	4.899302	-4.070476	-1.298289
17	1	0	4.854589	-4.430911	1.155583
18	6	0	2.985976	1.584705	-0.144404
19	6	0	4.255422	1.589002	0.446908
20	6	0	2.179240	2.725519	-0.059697
21	6	0	4.705738	2.722722	1.116673

22	1	0	4.887808	0.707881	0.391123
23	6	0	2.631442	3.856957	0.612430
24	1	0	1.195035	2.725452	-0.522179
25	6	0	3.894168	3.853177	1.201937
26	1	0	5.688755	2.722622	1.575111
27	1	0	1.999465	4.736061	0.678426
28	1	0	4.246564	4.732714	1.730550
29	6	0	-2.983684	1.589001	-0.163132
30	6	0	-4.232691	1.596110	0.469498
31	6	0	-2.186650	2.739793	-0.134707
32	6	0	-4.671770	2.742164	1.126108
33	1	0	-4.857936	0.708418	0.455496
34	6	0	-2.628025	3.883665	0.523038
35	1	0	-1.219028	2.737328	-0.631498
36	6	0	-3.869908	3.882222	1.155550
37	1	0	-5.638855	2.744218	1.617266
38	1	0	-2.003803	4.770484	0.545017
39	1	0	-4.213535	4.771344	1.673803
40	6	0	-3.214278	-1.274273	-0.355585
41	6	0	-3.836270	-2.217632	-1.178454
42	6	0	-3.177043	-1.474015	1.030591
43	6	0	-4.429988	-3.346568	-0.616937
44	1	0	-3.860028	-2.069811	-2.253626
45	6	0	-3.768471	-2.604213	1.583796
46	1	0	-2.682412	-0.752220	1.677208
47	6	0	-4.396351	-3.539878	0.761562
48	1	0	-4.917564	-4.072721	-1.258562
49	1	0	-3.735924	-2.755163	2.657696
50	1	0	-4.857536	-4.420216	1.196916
51	6	0	-0.003192	-0.515839	0.544475
52	6	0	-0.013698	0.378624	1.614205
53	6	0	0.005103	-1.892621	0.767978
54	6	0	-0.014956	-0.118106	2.921179
55	1	0	-0.022389	1.451602	1.444767
56	6	0	0.003124	-2.376462	2.075451
57	1	0	0.013407	-2.587701	-0.067217
58	6	0	-0.006653	-1.492245	3.152852
59	1	0	-0.023033	0.576896	3.755004
60	1	0	0.009678	-3.448228	2.247968
61	1	0	-0.007920	-1.872035	4.169106
62	1	0	3.982704	0.440449	-2.594499

011aca_Pd_PHPh22_Phcat_-H+_b3lyp631dp_PCM062X_.log

Standard orientation:

Center Number	Atomic Number	Atomic Type	Coordinates (Angstroms) X	Y	Z
1	46	0	0.123785	-0.059956	-1.177818
2	15	0	-2.366732	-0.028339	-1.207814
3	15	0	2.372578	-0.193343	-1.150529
4	8	0	2.705792	-0.358729	-2.636679
5	8	0	-3.057030	-0.009680	-2.704891
6	6	0	-3.180853	1.358359	-0.349506
7	6	0	-3.086501	1.467302	1.044395
8	6	0	-3.830616	2.358036	-1.078883
9	6	0	-3.647787	2.560470	1.695863
10	1	0	-2.572436	0.701456	1.621690
11	6	0	-4.396212	3.448597	-0.419893
12	1	0	-3.896480	2.284262	-2.160088
13	6	0	-4.305104	3.550815	0.965807
14	1	0	-3.567913	2.639083	2.775366
15	1	0	-4.906154	4.217140	-0.991351
16	1	0	-4.743293	4.401592	1.477291
17	6	0	-3.070465	-1.495094	-0.380457
18	6	0	-4.349871	-1.501629	0.188757
19	6	0	-2.305245	-2.667364	-0.365847
20	6	0	-4.852827	-2.665566	0.762826
21	1	0	-4.949364	-0.595570	0.189815
22	6	0	-2.808517	-3.830374	0.210940
23	1	0	-1.309984	-2.665785	-0.806285
24	6	0	-4.082137	-3.827625	0.776213
25	1	0	-5.844235	-2.665418	1.203398
26	1	0	-2.207511	-4.733496	0.222367
27	1	0	-4.474785	-4.731649	1.230147
28	6	0	3.013770	-1.591188	-0.154153
29	6	0	4.329943	-1.616572	0.320955
30	6	0	2.194815	-2.706242	0.052870
31	6	0	4.811794	-2.733304	0.999633
32	1	0	4.978538	-0.758829	0.165957
33	6	0	2.675159	-3.824469	0.729682
34	1	0	1.172488	-2.693335	-0.319775
35	6	0	3.984624	-3.836203	1.206533
36	1	0	5.832516	-2.742754	1.368095
37	1	0	2.029238	-4.682126	0.887875
38	1	0	4.360552	-4.703889	1.739035
39	6	0	3.255846	1.255525	-0.475987
40	6	0	3.769641	2.188152	-1.380149
41	6	0	3.378339	1.487625	0.899301
42	6	0	4.399942	3.342523	-0.917361
43	1	0	3.678888	1.991889	-2.444604

44	6	0	4.009309	2.639024	1.360013
45	1	0	2.980460	0.765586	1.608281
46	6	0	4.518198	3.568687	0.451983
47	1	0	4.800940	4.061585	-1.624565
48	1	0	4.103922	2.812903	2.427159
49	1	0	5.008781	4.466677	0.813772
50	6	0	0.138373	0.364143	0.756402
51	6	0	-0.100363	-0.617979	1.723378
52	6	0	0.208405	1.704148	1.153354
53	6	0	-0.284291	-0.260312	3.061820
54	1	0	-0.155678	-1.666358	1.443316
55	6	0	0.022742	2.057829	2.489811
56	1	0	0.411509	2.480762	0.420322
57	6	0	-0.227879	1.077725	3.448295
58	1	0	-0.473343	-1.033558	3.800848
59	1	0	0.073279	3.103595	2.779302
60	1	0	-0.373134	1.352764	4.488075
61	1	0	-4.022073	-0.106266	-2.687704

030aaa_Pd_PHPh22-H+_PPH2OH_Ph_b3lyp631dp_PCM062X_.log

Standard orientation:

Center Number	Atomic Number	Atomic Type	Coordinates (Angstroms) X	Y	Z
1	46	0	0.177462	-0.293370	0.063477
2	15	0	-1.260115	-2.183891	-0.183919
3	15	0	2.095817	-1.539584	-0.380008
4	8	0	1.820005	-3.020230	-0.803472
5	1	0	0.532099	-3.374750	-0.677456
6	8	0	-0.530127	-3.558205	-0.565891
7	6	0	-2.346482	-1.850084	-1.611855
8	6	0	-3.633532	-1.318232	-1.492023
9	6	0	-1.772319	-1.988389	-2.882226
10	6	0	-4.334515	-0.925165	-2.631098
11	1	0	-4.078137	-1.196831	-0.509367
12	6	0	-2.473597	-1.591731	-4.017413
13	1	0	-0.771019	-2.403023	-2.975321
14	6	0	-3.754912	-1.054462	-3.891364
15	1	0	-5.334154	-0.514110	-2.532778
16	1	0	-2.021957	-1.700259	-4.998249
17	1	0	-4.300519	-0.739789	-4.775150
18	6	0	-2.374028	-2.606486	1.189280
19	6	0	-3.401273	-3.542898	1.025232
20	6	0	-2.157451	-2.032300	2.444628
21	6	0	-4.203512	-3.895511	2.106217
22	1	0	-3.576172	-3.989682	0.049835

23	6	0	-2.960152	-2.385867	3.527054
24	1	0	-1.364780	-1.297250	2.569008
25	6	0	-3.982679	-3.316475	3.356378
26	1	0	-5.000813	-4.619765	1.975558
27	1	0	-2.789141	-1.935068	4.499185
28	1	0	-4.610231	-3.591762	4.197885
29	6	0	3.149095	-1.587189	1.119264
30	6	0	4.543206	-1.676988	1.061266
31	6	0	2.516094	-1.634542	2.367598
32	6	0	5.290576	-1.794262	2.231781
33	1	0	5.049736	-1.650724	0.101060
34	6	0	3.261437	-1.756183	3.537073
35	1	0	1.430610	-1.566887	2.424165
36	6	0	4.651856	-1.831360	3.469416
37	1	0	6.372513	-1.857853	2.176190
38	1	0	2.759465	-1.787551	4.498688
39	1	0	5.235938	-1.920168	4.379710
40	6	0	3.183017	-0.869296	-1.689450
41	6	0	3.164418	-1.508232	-2.932799
42	6	0	3.986874	0.264544	-1.512766
43	6	0	3.932848	-1.019993	-3.988840
44	1	0	2.554084	-2.397155	-3.058823
45	6	0	4.757974	0.745498	-2.566751
46	1	0	4.009677	0.774404	-0.553036
47	6	0	4.729600	0.107559	-3.806953
48	1	0	3.912254	-1.523873	-4.949910
49	1	0	5.378681	1.623747	-2.419410
50	1	0	5.329749	0.488017	-4.627392
51	6	0	1.415306	1.343037	0.110037
52	6	0	2.161872	1.683978	1.244717
53	6	0	1.496066	2.189931	-1.003966
54	6	0	2.968876	2.823656	1.264596
55	1	0	2.123109	1.053752	2.130591
56	6	0	2.296244	3.333832	-0.989394
57	1	0	0.931931	1.957229	-1.905226
58	6	0	3.038638	3.653606	0.146690
59	1	0	3.540995	3.064348	2.156472
60	1	0	2.342093	3.972491	-1.867251
61	1	0	3.664444	4.540576	0.160787
62	15	0	-1.561419	1.278071	0.509790
63	8	0	-2.984901	0.582714	0.960865
64	6	0	-1.999071	2.281713	-0.947530
65	6	0	-2.530592	3.572340	-0.843755
66	6	0	-1.875560	1.685598	-2.209334
67	6	0	-2.922686	4.259622	-1.989745
68	1	0	-2.634692	4.043833	0.128934
69	6	0	-2.274831	2.372854	-3.351753
70	1	0	-1.471856	0.679265	-2.300758
71	6	0	-2.794114	3.661626	-3.242214
72	1	0	-3.329318	5.261754	-1.903941

73	1	0	-2.180363	1.899201	-4.323552
74	1	0	-3.100698	4.200307	-4.132958
75	6	0	-1.276007	2.479905	1.850661
76	6	0	-1.855464	2.247480	3.102707
77	6	0	-0.425678	3.579474	1.675410
78	6	0	-1.597777	3.112510	4.164874
79	1	0	-2.510230	1.392939	3.245919
80	6	0	-0.174405	4.440369	2.738828
81	1	0	0.046200	3.759750	0.712603
82	6	0	-0.759136	4.209138	3.983671
83	1	0	-2.054456	2.928163	5.131640
84	1	0	0.484691	5.290305	2.594339
85	1	0	-0.559169	4.882339	4.810896
86	1	0	-3.713206	1.220183	1.030323

032baa_Pd_PHPh22-H+_PPH2OH_Ph_b3lyp631dp_PCM062X_TS_CP_f.log

Standard orientation:

Center Number	Atomic Number	Atomic Type	Coordinates (Angstroms) X	Y	Z
1	46	0	-0.044959	-0.034830	-0.100955
2	15	0	0.599359	2.186630	-0.560438
3	15	0	-2.256377	0.142504	-0.928901
4	8	0	-1.917022	0.950720	-2.213397
5	1	0	-0.948775	2.072544	-2.045774
6	8	0	-0.240209	2.787247	-1.809989
7	6	0	2.334070	2.508294	-1.039967
8	6	0	3.363393	2.248326	-0.127001
9	6	0	2.650906	2.890445	-2.344889
10	6	0	4.692656	2.384618	-0.512919
11	1	0	3.128415	1.910386	0.880383
12	6	0	3.985190	3.019494	-2.730554
13	1	0	1.849920	3.082344	-3.051729
14	6	0	5.005895	2.767918	-1.817183
15	1	0	5.485524	2.176095	0.198663
16	1	0	4.225718	3.316428	-3.746675
17	1	0	6.043503	2.863261	-2.120985
18	6	0	0.306407	3.370332	0.801185
19	6	0	-0.385019	4.562436	0.578650
20	6	0	0.695323	3.026527	2.101659
21	6	0	-0.675795	5.410087	1.646384
22	1	0	-0.705361	4.811064	-0.428544
23	6	0	0.416610	3.882153	3.163948
24	1	0	1.195426	2.076246	2.286023
25	6	0	-0.272760	5.073737	2.937122
26	1	0	-1.219442	6.333025	1.470558

27	1	0	0.721391	3.612645	4.170294
28	1	0	-0.502390	5.733846	3.767425
29	6	0	-3.039179	1.187922	0.366778
30	6	0	-4.274022	1.783151	0.074693
31	6	0	-2.388057	1.527060	1.556944
32	6	0	-4.837284	2.704315	0.952391
33	1	0	-4.792292	1.528885	-0.845844
34	6	0	-2.955740	2.442674	2.440667
35	1	0	-1.418524	1.092856	1.790134
36	6	0	-4.178897	3.034495	2.136984
37	1	0	-5.789472	3.165705	0.711811
38	1	0	-2.428437	2.705062	3.352366
39	1	0	-4.618457	3.755119	2.819131
40	6	0	-3.721641	-0.827955	-1.523197
41	6	0	-3.961321	-0.908188	-2.895520
42	6	0	-4.591306	-1.477153	-0.639095
43	6	0	-5.047646	-1.638880	-3.380390
44	1	0	-3.297428	-0.386192	-3.575790
45	6	0	-5.680681	-2.193985	-1.121123
46	1	0	-4.418855	-1.419440	0.432761
47	6	0	-5.907589	-2.281719	-2.495956
48	1	0	-5.222102	-1.698690	-4.450097
49	1	0	-6.353671	-2.685911	-0.426069
50	1	0	-6.754547	-2.846735	-2.871856
51	6	0	-1.482427	-1.638714	-0.097242
52	6	0	-1.877409	-1.985640	1.200229
53	6	0	-1.166611	-2.655936	-1.013314
54	6	0	-1.927661	-3.327303	1.583951
55	1	0	-2.142807	-1.212666	1.916914
56	6	0	-1.207432	-3.988557	-0.622989
57	1	0	-0.889032	-2.397310	-2.033293
58	6	0	-1.587187	-4.326554	0.678495
59	1	0	-2.219716	-3.586614	2.596997
60	1	0	-0.937637	-4.767501	-1.329677
61	1	0	-1.611493	-5.367777	0.983125
62	15	0	1.960287	-1.165422	0.810408
63	8	0	2.533732	-0.360110	2.144279
64	6	0	3.393600	-1.263239	-0.318438
65	6	0	4.629782	-1.784794	0.082380
66	6	0	3.252514	-0.743751	-1.608783
67	6	0	5.712830	-1.767192	-0.790216
68	1	0	4.747110	-2.214389	1.074951
69	6	0	4.333990	-0.737298	-2.488003
70	1	0	2.295622	-0.326852	-1.916509
71	6	0	5.564048	-1.242510	-2.075466
72	1	0	6.670683	-2.166670	-0.473419
73	1	0	4.218458	-0.324146	-3.484632
74	1	0	6.410711	-1.230071	-2.754401
75	6	0	1.829599	-2.879959	1.431880
76	6	0	1.387074	-3.101941	2.741267

77	6	0	2.039935	-3.974767	0.585624
78	6	0	1.174620	-4.398690	3.201390
79	1	0	1.213385	-2.256816	3.402202
80	6	0	1.818916	-5.269846	1.045526
81	1	0	2.382876	-3.815216	-0.433476
82	6	0	1.386944	-5.483809	2.353314
83	1	0	0.839634	-4.561108	4.220902
84	1	0	1.987548	-6.112916	0.383040
85	1	0	1.218124	-6.494418	2.711172
86	1	0	3.291627	-0.794044	2.565408

033aaa_Pd_PHPh22-H+_PPH2OH_Ph_b3lyp631dp_PCM062X_SCAN_CP_vege.log

Standard orientation:

Center Number	Atomic Number	Atomic Type	Coordinates (Angstroms) X	Y	Z
1	46	0	0.598197	0.367868	-0.703443
2	15	0	2.329058	-1.238266	-0.551438
3	15	0	-1.623262	-2.401045	0.048843
4	8	0	-0.525670	-3.396790	-0.283844
5	1	0	1.069910	-3.051821	-0.546247
6	8	0	2.036581	-2.854112	-0.672503
7	6	0	3.249349	-1.163347	1.037689
8	6	0	3.637633	0.085157	1.537310
9	6	0	3.498659	-2.310080	1.794816
10	6	0	4.260313	0.186878	2.777689
11	1	0	3.437144	0.982713	0.954627
12	6	0	4.120242	-2.208291	3.040348
13	1	0	3.192209	-3.277771	1.408347
14	6	0	4.497593	-0.961697	3.534920
15	1	0	4.553292	1.160635	3.157841
16	1	0	4.305622	-3.103698	3.625371
17	1	0	4.974061	-0.883325	4.506778
18	6	0	3.693561	-1.074414	-1.762999
19	6	0	4.859651	-1.842163	-1.657303
20	6	0	3.551699	-0.184287	-2.830243
21	6	0	5.866085	-1.721728	-2.609482
22	1	0	4.977628	-2.530803	-0.824178
23	6	0	4.563673	-0.058874	-3.782681
24	1	0	2.643492	0.411034	-2.907695
25	6	0	5.718714	-0.827625	-3.672018
26	1	0	6.768098	-2.319155	-2.523791
27	1	0	4.448446	0.636738	-4.607642
28	1	0	6.507568	-0.731449	-4.411206
29	6	0	-1.945912	-1.304597	-1.356072
30	6	0	-1.057334	-1.354449	-2.436521

31	6	0	-3.030578	-0.419397	-1.383474
32	6	0	-1.238141	-0.499099	-3.524030
33	1	0	-0.239779	-2.069284	-2.423806
34	6	0	-3.204810	0.431675	-2.469039
35	1	0	-3.747497	-0.395649	-0.566953
36	6	0	-2.304914	0.397319	-3.534277
37	1	0	-0.545088	-0.536191	-4.358354
38	1	0	-4.031811	1.134362	-2.475132
39	1	0	-2.440218	1.070220	-4.375232
40	6	0	-3.209325	-3.173178	0.453568
41	6	0	-3.523905	-3.487983	1.780163
42	6	0	-4.089740	-3.531565	-0.574286
43	6	0	-4.711082	-4.152584	2.074625
44	1	0	-2.846209	-3.210018	2.582118
45	6	0	-5.274951	-4.196106	-0.275276
46	1	0	-3.852909	-3.285240	-1.605526
47	6	0	-5.585536	-4.504433	1.048378
48	1	0	-4.954576	-4.391612	3.104236
49	1	0	-5.957205	-4.468886	-1.073143
50	1	0	-6.512650	-5.018178	1.280497
51	6	0	-1.163100	-1.404777	1.492667
52	6	0	-1.987099	-0.399767	2.015075
53	6	0	0.072801	-1.667722	2.086895
54	6	0	-1.562650	0.341084	3.113221
55	1	0	-2.960849	-0.198301	1.576367
56	6	0	0.500669	-0.918061	3.181723
57	1	0	0.701399	-2.456107	1.683628
58	6	0	-0.319093	0.083398	3.694077
59	1	0	-2.200930	1.120047	3.518269
60	1	0	1.475639	-1.116132	3.619236
61	1	0	0.008542	0.671659	4.545274
62	15	0	-0.087471	2.573487	-0.558246
63	8	0	0.727441	3.569789	-1.609889
64	6	0	0.209352	3.367416	1.070284
65	6	0	-0.257229	4.656232	1.363494
66	6	0	0.965238	2.681625	2.024556
67	6	0	0.041698	5.250700	2.584701
68	1	0	-0.865837	5.192967	0.638821
69	6	0	1.258930	3.274016	3.252622
70	1	0	1.310075	1.672981	1.806186
71	6	0	0.801151	4.558748	3.530511
72	1	0	-0.320712	6.249730	2.804045
73	1	0	1.843588	2.729061	3.987784
74	1	0	1.029793	5.022953	4.484498
75	6	0	-1.823184	3.086141	-0.862735
76	6	0	-2.200439	3.690294	-2.063838
77	6	0	-2.806334	2.774972	0.084821
78	6	0	-3.539899	3.996608	-2.307558
79	1	0	-1.447983	3.920787	-2.812426
80	6	0	-4.142316	3.072226	-0.161567

81	1	0	-2.520170	2.302672	1.022757
82	6	0	-4.511551	3.688860	-1.359028
83	1	0	-3.821835	4.473990	-3.240682
84	1	0	-4.895945	2.830296	0.581192
85	1	0	-5.553222	3.925909	-1.549666
86	1	0	0.502830	4.506463	-1.495686

102gca_Pd_PHPh22_Ph_Br_b3lyp631dp_PCM062X_SCAN_CBr_f_vege.log

Standard orientation:

Center Number	Atomic Number	Atomic Type	Coordinates (Angstroms) X	Y	Z
1	46	0	0.011850	-0.621537	-0.897174
2	15	0	2.150658	-1.429756	-0.884339
3	15	0	-0.545330	0.842526	0.839604
4	8	0	0.054255	0.306406	2.297408
5	1	0	0.592069	-0.486721	2.152837
6	8	0	3.031389	-2.420057	-1.904963
7	1	0	2.430231	-2.846122	-2.527999
8	6	0	2.512451	-2.206417	0.744302
9	6	0	1.455107	-2.725677	1.500390
10	6	0	3.817665	-2.305700	1.240759
11	6	0	1.693587	-3.318167	2.741365
12	1	0	0.440227	-2.664838	1.107744
13	6	0	4.057798	-2.905408	2.473062
14	1	0	4.645162	-1.902987	0.662226
15	6	0	2.995378	-3.406426	3.227135
16	1	0	0.865446	-3.710960	3.322335
17	1	0	5.072383	-2.976550	2.851670
18	1	0	3.184731	-3.865189	4.192131
19	6	0	3.315485	-0.012436	-0.838253
20	6	0	3.230984	0.909440	0.213504
21	6	0	4.218933	0.220217	-1.877325
22	6	0	4.027110	2.048912	0.220874
23	1	0	2.530490	0.741895	1.029068
24	6	0	5.022399	1.361395	-1.866376
25	1	0	4.295025	-0.496486	-2.688506
26	6	0	4.924596	2.278403	-0.823320
27	1	0	3.936980	2.761695	1.034808
28	1	0	5.723902	1.532681	-2.676860
29	1	0	5.545059	3.168852	-0.821454
30	6	0	-2.229378	1.300455	1.390287
31	6	0	-2.857614	2.465596	0.939459
32	6	0	-2.966310	0.369435	2.131145
33	6	0	-4.202481	2.694601	1.222673
34	1	0	-2.297919	3.193186	0.357412

35	6	0	-4.308129	0.602483	2.420832
36	1	0	-2.486648	-0.541782	2.477841
37	6	0	-4.930813	1.763286	1.961574
38	1	0	-4.681187	3.601808	0.867729
39	1	0	-4.869707	-0.124486	2.999071
40	1	0	-5.978452	1.942059	2.180840
41	6	0	0.263833	2.476518	0.684651
42	6	0	0.730898	3.170706	1.804940
43	6	0	0.454260	3.015331	-0.590861
44	6	0	1.381496	4.391706	1.647560
45	1	0	0.597009	2.742930	2.794383
46	6	0	1.090843	4.245515	-0.746327
47	1	0	0.117147	2.457549	-1.463416
48	6	0	1.559101	4.930644	0.372655
49	1	0	1.750480	4.924532	2.518301
50	1	0	1.235156	4.659855	-1.738949
51	1	0	2.067510	5.882003	0.252864
52	6	0	-2.968643	-1.140787	-1.409349
53	6	0	-3.642614	0.067144	-1.276195
54	6	0	-2.036989	-1.359203	-2.425986
55	6	0	-3.349105	1.097175	-2.167896
56	1	0	-4.355674	0.214037	-0.471221
57	6	0	-1.744409	-0.308913	-3.300444
58	1	0	-1.567028	-2.330118	-2.543291
59	6	0	-2.393930	0.919634	-3.166956
60	1	0	-3.860951	2.048302	-2.062207
61	1	0	-1.017543	-0.464245	-4.091277
62	1	0	-2.163843	1.729959	-3.850390
63	35	0	-3.308266	-2.534836	-0.164328

102gea_Pd_PHPh22_Ph_Br_b3lyp631dp_PCM062X_TS_CBr_f2.log

Standard orientation:

Center Number	Atomic Number	Atomic Type	Coordinates (Angstroms) X	Y	Z
1	46	0	-0.144476	-0.796899	-0.765717
2	15	0	-2.475729	-0.122783	-0.743201
3	15	0	0.879539	0.844446	0.770340
4	8	0	-0.074604	1.508776	1.959482
5	1	0	-0.984378	1.615955	1.638937
6	8	0	-3.386666	-0.007654	-2.125870
7	1	0	-3.058647	-0.632376	-2.786767
8	6	0	-2.544587	1.626794	-0.199533
9	6	0	-1.721144	2.529140	-0.890086
10	6	0	-3.246312	2.069853	0.927630
11	6	0	-1.595406	3.845848	-0.461855

12	1	0	-1.146094	2.184477	-1.747270
13	6	0	-3.112547	3.391135	1.360752
14	1	0	-3.884426	1.384591	1.477345
15	6	0	-2.287072	4.276020	0.671288
16	1	0	-0.934689	4.524387	-0.992648
17	1	0	-3.651542	3.723799	2.241623
18	1	0	-2.175535	5.297140	1.020528
19	6	0	-3.609317	-0.944276	0.423534
20	6	0	-3.060334	-1.607060	1.526033
21	6	0	-4.997052	-0.912719	0.252042
22	6	0	-3.893303	-2.218569	2.460862
23	1	0	-1.978354	-1.646561	1.645508
24	6	0	-5.826356	-1.535182	1.181065
25	1	0	-5.418892	-0.405675	-0.610905
26	6	0	-5.275452	-2.183334	2.286882
27	1	0	-3.464302	-2.730010	3.316254
28	1	0	-6.902756	-1.513713	1.045025
29	1	0	-5.924880	-2.666264	3.009755
30	6	0	1.585501	2.330743	-0.028602
31	6	0	1.772783	3.517466	0.688601
32	6	0	1.890106	2.290849	-1.391776
33	6	0	2.262547	4.650722	0.047090
34	1	0	1.515566	3.550366	1.743640
35	6	0	2.389877	3.424622	-2.031879
36	1	0	1.718510	1.374945	-1.956174
37	6	0	2.572760	4.603930	-1.313260
38	1	0	2.400972	5.571899	0.604036
39	1	0	2.624433	3.389912	-3.090843
40	1	0	2.952018	5.489622	-1.812745
41	6	0	2.238091	0.272426	1.849389
42	6	0	1.931969	-0.720108	2.788680
43	6	0	3.562555	0.691357	1.704190
44	6	0	2.930668	-1.269734	3.584392
45	1	0	0.906844	-1.070960	2.887944
46	6	0	4.565481	0.132771	2.496498
47	1	0	3.815508	1.454203	0.973677
48	6	0	4.251522	-0.844761	3.437043
49	1	0	2.682833	-2.036635	4.311287
50	1	0	5.592088	0.464611	2.378278
51	1	0	5.033489	-1.278449	4.052067
52	6	0	1.497457	-2.120823	-1.042867
53	6	0	2.805065	-1.786047	-0.656870
54	6	0	0.887822	-3.297790	-0.588322
55	6	0	3.444277	-2.575750	0.284249
56	1	0	3.278684	-0.892629	-1.052255
57	6	0	1.553575	-4.070795	0.372396
58	1	0	-0.070418	-3.612389	-0.989849
59	6	0	2.822672	-3.716845	0.810047
60	1	0	4.431324	-2.284869	0.631232
61	1	0	1.075982	-4.971753	0.745435

62	1	0	3.339051	-4.326927	1.543175
63	35	0	0.963755	-1.578072	-3.067571

104aca_Pd_PHPh22_Ph_Br_-H+_b3lyp631dp_PCMmecn.log

Standard orientation:

Center Number	Atomic Number	Atomic Type	Coordinates (Angstroms) X	Y	Z
1	46	0	0.109623	-0.265535	0.934481
2	15	0	2.434165	-0.434067	0.819175
3	15	0	-2.313016	-0.145048	0.668536
4	8	0	-3.162009	-0.640576	1.984446
5	1	0	-2.528853	-1.088337	2.583317
6	8	0	3.119746	-1.039009	2.047667
7	6	0	3.335308	1.130888	0.439389
8	6	0	3.295101	1.804044	-0.789149
9	6	0	4.084038	1.682064	1.484086
10	6	0	3.990292	2.997624	-0.962808
11	1	0	2.719793	1.395848	-1.615884
12	6	0	4.773379	2.882391	1.313886
13	1	0	4.127796	1.147001	2.428044
14	6	0	4.727459	3.542581	0.088735
15	1	0	3.951522	3.505931	-1.921624
16	1	0	5.349722	3.297107	2.135241
17	1	0	5.264895	4.475657	-0.049251
18	6	0	2.779270	-1.486543	-0.661684
19	6	0	3.865528	-1.294669	-1.520512
20	6	0	1.941136	-2.588191	-0.880066
21	6	0	4.088005	-2.162243	-2.589804
22	1	0	4.540347	-0.458740	-1.362476
23	6	0	2.160601	-3.458746	-1.944155
24	1	0	1.105342	-2.762108	-0.203850
25	6	0	3.233675	-3.240629	-2.807775
26	1	0	4.930857	-1.994947	-3.253290
27	1	0	1.495935	-4.302928	-2.100746
28	1	0	3.405437	-3.911597	-3.643438
29	6	0	-2.919178	-1.209408	-0.687369
30	6	0	-4.218448	-1.089715	-1.195638
31	6	0	-2.074739	-2.204397	-1.189941
32	6	0	-4.662904	-1.954635	-2.190537
33	1	0	-4.878922	-0.315075	-0.815608
34	6	0	-2.518415	-3.067608	-2.189904
35	1	0	-1.064028	-2.298997	-0.799816
36	6	0	-3.812205	-2.941802	-2.689510
37	1	0	-5.670966	-1.858174	-2.580263
38	1	0	-1.854250	-3.833332	-2.577570

39	1	0	-4.159635	-3.611383	-3.469686
40	6	0	-3.130621	1.442332	0.319322
41	6	0	-3.853380	2.104226	1.315330
42	6	0	-2.979700	2.037430	-0.939798
43	6	0	-4.428081	3.347037	1.051563
44	1	0	-3.973262	1.638630	2.288051
45	6	0	-3.552459	3.278521	-1.196533
46	1	0	-2.406538	1.535271	-1.716775
47	6	0	-4.278036	3.934724	-0.202007
48	1	0	-4.994353	3.853467	1.826501
49	1	0	-3.427470	3.735457	-2.173061
50	1	0	-4.724656	4.902734	-0.405129
51	6	0	0.230067	0.914578	-0.667449
52	6	0	0.247509	0.403628	-1.971305
53	6	0	0.202182	2.305449	-0.502504
54	6	0	0.242172	1.259408	-3.076454
55	1	0	0.281872	-0.670981	-2.135471
56	6	0	0.201114	3.161308	-1.602677
57	1	0	0.187602	2.731098	0.498680
58	6	0	0.221481	2.641743	-2.896245
59	1	0	0.258826	0.841898	-4.079546
60	1	0	0.182367	4.236764	-1.448452
61	1	0	0.220404	3.306018	-3.754884
62	35	0	-0.342928	-1.833903	3.052496

105caa_Pd_PHPh22_-H+_Ph_Br_b3lyp631dp_PCMmecn_TS_CBr_jo_f7.log

Standard orientation:

Center Number	Atomic Number	Atomic Type	Coordinates (Angstroms) X	Y	Z
1	46	0	0.308826	-0.750222	-0.862341
2	15	0	-2.101096	-1.038591	-1.001955
3	15	0	0.920846	1.583568	-0.798845
4	8	0	0.447089	2.514100	-1.942775
5	8	0	-2.905576	-2.351102	-1.644323
6	1	0	-2.339620	-2.786040	-2.294103
7	6	0	-3.245658	0.321261	-1.445416
8	6	0	-2.732115	1.566493	-1.813587
9	6	0	-4.632373	0.127691	-1.390240
10	6	0	-3.605585	2.615084	-2.113239
11	1	0	-1.655853	1.731847	-1.868799
12	6	0	-5.496634	1.169767	-1.704223
13	1	0	-5.028326	-0.843388	-1.104027
14	6	0	-4.981833	2.418553	-2.061892
15	1	0	-3.203548	3.584292	-2.393081
16	1	0	-6.570338	1.015320	-1.665236

17	1	0	-5.657682	3.233766	-2.300673
18	6	0	-2.553372	-1.270070	0.760512
19	6	0	-2.489587	-0.179819	1.637642
20	6	0	-2.845566	-2.541102	1.262426
21	6	0	-2.720165	-0.363963	2.997928
22	1	0	-2.250152	0.814644	1.261862
23	6	0	-3.077297	-2.720729	2.625669
24	1	0	-2.894252	-3.385574	0.581969
25	6	0	-3.013545	-1.633921	3.495347
26	1	0	-2.664511	0.488309	3.668037
27	1	0	-3.308479	-3.710414	3.007306
28	1	0	-3.192520	-1.774652	4.556542
29	6	0	2.740563	1.875584	-0.562616
30	6	0	3.365821	2.296355	0.614344
31	6	0	3.553069	1.524501	-1.651623
32	6	0	4.758083	2.343903	0.708815
33	1	0	2.767512	2.578943	1.475402
34	6	0	4.940681	1.577597	-1.567362
35	1	0	3.084542	1.185281	-2.574254
36	6	0	5.549740	1.979598	-0.377031
37	1	0	5.224160	2.665154	1.635923
38	1	0	5.548533	1.297333	-2.422496
39	1	0	6.632034	2.011074	-0.300342
40	6	0	0.218093	2.249977	0.774652
41	6	0	-0.714321	3.288398	0.712199
42	6	0	0.448235	1.621091	2.006253
43	6	0	-1.397399	3.700792	1.858386
44	1	0	-0.906245	3.752634	-0.251872
45	6	0	-0.214089	2.044326	3.153751
46	1	0	1.141157	0.782525	2.059377
47	6	0	-1.145368	3.083502	3.081967
48	1	0	-2.127032	4.503187	1.796444
49	1	0	-0.022025	1.549757	4.102101
50	1	0	-1.676032	3.402279	3.974078
51	6	0	1.845007	-1.674044	0.330707
52	6	0	3.002821	-1.024644	0.791092
53	6	0	0.979440	-2.313292	1.234555
54	6	0	3.196508	-0.893565	2.158298
55	1	0	3.703362	-0.586952	0.087659
56	6	0	1.180931	-2.137113	2.606987
57	1	0	0.160379	-2.926574	0.871523
58	6	0	2.283568	-1.431058	3.076332
59	1	0	4.066290	-0.346226	2.510419
60	1	0	0.480293	-2.590973	3.302203
61	1	0	2.451079	-1.319770	4.142173
62	35	0	2.063343	-2.714889	-1.533870

106aaa_Pd_PHPh22_-H+_Ph_Br_b3lyp631dp_PCMmecn_SCAN_CBr_vege_uj.log

Standard orientation:

Center Number	Atomic Number	Atomic Type	Coordinates (Angstroms) X	Y	Z
1	46	0	0.413570	-1.153420	-0.719144
2	15	0	2.431863	-0.091250	-0.659600
3	15	0	-1.742969	-2.183607	-0.544172
4	8	0	-2.089333	-3.613696	-1.019656
5	8	0	2.915115	0.855411	-1.942230
6	1	0	2.127798	1.227885	-2.364279
7	6	0	3.973580	-1.060551	-0.478315
8	6	0	3.902642	-2.455073	-0.506830
9	6	0	5.217421	-0.435484	-0.328093
10	6	0	5.062389	-3.220901	-0.384515
11	1	0	2.933104	-2.934776	-0.624995
12	6	0	6.374186	-1.198589	-0.215600
13	1	0	5.276263	0.649612	-0.296688
14	6	0	6.296083	-2.592707	-0.241230
15	1	0	5.001018	-4.304133	-0.404334
16	1	0	7.336873	-0.710600	-0.101989
17	1	0	7.199691	-3.186557	-0.147592
18	6	0	2.590457	1.099283	0.729674
19	6	0	2.112008	0.722781	1.989764
20	6	0	3.123742	2.378806	0.551594
21	6	0	2.181081	1.607162	3.062519
22	1	0	1.664082	-0.260798	2.122063
23	6	0	3.182791	3.268610	1.624547
24	1	0	3.476320	2.677297	-0.431127
25	6	0	2.712857	2.884451	2.879657
26	1	0	1.804642	1.308327	4.035834
27	1	0	3.590894	4.263769	1.478660
28	1	0	2.754196	3.580020	3.711683
29	6	0	-3.011259	-1.025120	-1.272641
30	6	0	-4.265961	-0.780296	-0.701565
31	6	0	-2.712597	-0.415441	-2.497321
32	6	0	-5.188936	0.054847	-1.329672
33	1	0	-4.519396	-1.237317	0.252077
34	6	0	-3.633848	0.411904	-3.138530
35	1	0	-1.734052	-0.583988	-2.945575
36	6	0	-4.875486	0.652227	-2.551220
37	1	0	-6.153852	0.241605	-0.867112
38	1	0	-3.380262	0.877484	-4.086334
39	1	0	-5.593551	1.303825	-3.039448
40	6	0	-2.258505	-2.073309	1.232671
41	6	0	-2.896983	-3.156720	1.838534
42	6	0	-1.979647	-0.935571	1.999699
43	6	0	-3.257660	-3.107288	3.186359

44	1	0	-3.100537	-4.037267	1.234434
45	6	0	-2.342990	-0.879469	3.342318
46	1	0	-1.473721	-0.086897	1.537456
47	6	0	-2.981180	-1.968691	3.940181
48	1	0	-3.752323	-3.956605	3.648910
49	1	0	-2.124118	0.011759	3.923993
50	1	0	-3.256621	-1.929239	4.989654
51	6	0	-1.199528	2.462016	-0.077561
52	6	0	-2.559302	2.172631	-0.084985
53	6	0	-0.506009	2.729253	1.096960
54	6	0	-3.244644	2.170773	1.127965
55	1	0	-3.072230	1.947725	-1.013747
56	6	0	-1.209100	2.717765	2.301303
57	1	0	0.555457	2.949770	1.074857
58	6	0	-2.576790	2.451194	2.318369
59	1	0	-4.304930	1.938645	1.132146
60	1	0	-0.675342	2.924339	3.223809
61	1	0	-3.118513	2.449606	3.258486
62	35	0	-0.274219	2.550058	-1.741507
